# Development of two loop-mediated isothermal amplification (LAMP) genomics-informed diagnostic protocols for rapid detection of *Pantoea* species on rice

**DOI:** 10.1016/j.mex.2021.101216

**Published:** 2021-01-06

**Authors:** Kossi Kini, Issa Wonni, Drissa Silué, Ralf Koebnik

**Affiliations:** a Plant Pathology Unit, AfricaRice, Cotonou, Benin; b IPME, Univ Montpellier, CIRAD, IRD, Montpellier, France; c Plant Pathology, Institut de l'Environnement et de Recherches Agricoles (INERA), Bobo Dioulasso, Burkina Faso; d Plant Pathology Unit, AfricaRice, Bouaké, Côte d'Ivoire

**Keywords:** Diagnostic protocol, Loop-mediated isothermal amplification (LAMP), Pantoea spp., Rice

## Abstract

At least three species of *Pantoea* are responsible for bacterial blight disease and grain discoloration of rice in Sub-Saharan Africa. Thus, measures need to be taken to limit the pathogens' dispersion and robust diagnostic tools are required for rapid and cheap diagnosis in the field as well as for routine seed certification or control. Therefore, several diagnostic tools such as simplex and multiplex PCR schemes and a semi-selective medium have been developed and are being used. However, the use of these tools is time-consuming, expensive and therefore limited to laboratories that can afford the chemicals. We have therefore developed two isothermal loop amplification (LAMP) protocols, one of which detects all *Pantoea* species in the genus and another one that is specific for *P. ananatis*.

• The novel LAMP assays allow rapid and sensitive detection of these bacteria.
• They will help plant protection services in routine field and laboratory tests especially for monitoring the phytosanitary status of rice seeds.

## Graphical abstract

**Figure:**
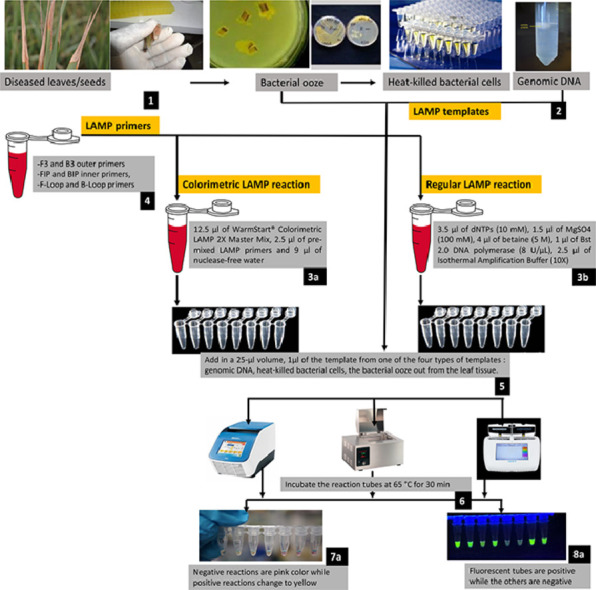

Specifications table

**Table:** 

Subject Area:	Agricultural and Biological Sciences
More specific subject area:	Plant Pathology, Molecular plant disease diagnosis tools
Protocol name:	LAMP assays for the diagnosis of Pantoea spp. blights of rice
Reagents/tools:	Materials and Reagents 1. Axygen^Ⓡ^ MicroVolume Extended-Length Filtered Pipet Tips (Axygen, catalog number: TXLF-10RS) 2. Axygen^Ⓡ^ Universal Fit 100 µl Filtered Pipet Tips (Axygen, catalog number: 383-TF-100-LRS) 3. Axygen^Ⓡ^ Universal Fit 200 µl Filtered Pipet Tips (Axygen, catalog number: 383-TF-200-RS) 4. Axygen^Ⓡ^ Universal Fit 1,000 µl Filtered Pipet Tips (Axygen, catalog number: 383-TF-1000-LRS) 5. Axygen 1.5 ml Snaplock Microtubes (Axygen, catalog number: MCT-150-CS) 6. Axygen 0.2 ml PCR^Ⓡ^ Tubes (Axygen, catalog number: PCR-02-C) 7. LightCycler^Ⓡ^ 480 Multiwell Plate 96, white (LifeScience Roche, catalog number: 4729692001) 8. LightCycler^Ⓡ^ 8-Tube Strips, white (LifeScience Roche, catalog number: 6612601001) Wizard^Ⓡ^ Genomic DNA Purification Kit (Promega, catalog numbers: A1120, A1123, A1125 and A1620) 9. WarmStart^Ⓡ^ Colorimetric LAMP 2x Master Mix (DNA & RNA) (with cresol red) (NEB, catalog number: M1800L) 10. Isothermal Amplification Buffer Pack (NEB, catalog number: B0537S) 11. Magnesium sulfate (MgSO_4_) solution (NEB, catalog number: B1003S) 12. Betaine (Sigma Aldrich; 5 M) 13. dNTP Set, 100 mM solutions (Thermo Fisher, catalog number: R0186) 14. Green Fluorescent Nucleic Acid Stain (Invitrogen, catalog number: S34854) 15. Nuclease-Free water (not DEPC-treated) (Ambion^TM^, catalog number: AM9938) 16. Agarose (Biowest, catalog number: BY-R0100) 17. Ultra-pure water (Genview, catalog number: GU3313-500) 18. GelRed^Ⓡ^ Nucleic Acid Stain 10000X DMSO (Merck, Catalogue Number: SCT122) 19. Agar (Shangxiang, catalog number: 120420) 20. Peptone (Shangxiang, catalog number: 120420) 21. Sucrose (Shangxiang, catalog number: 120420) 22. 50x TBE buffer (Meilunbio, catalog number: MA0004) 23. 2000 DNA Marker (Yeasen, catalog number: 10501ES60) 24. 5000 DNA Marker (Yeasen, catalog number: 10504ES60) 25. Solid PSA and PGSA medium (see Recipes) 26. 1.5 % agarose gel (see Recipes) Equipment 1. 0.5-10 µl Eppendorf Research^Ⓡ^ plus Adjustable Volume Pipettes (Eppendorf, Catalog #: EP-START) 2. 10-100 µl Eppendorf Research^Ⓡ^ plus Adjustable Volume Pipettes (Eppendorf, Catalog #: EP-START) 3. 20-200 µl Eppendorf Research^Ⓡ^ plus Adjustable Volume Pipettes (Eppendorf, Catalog #: EP-START) 4. 100-1,000 µl Eppendorf Research^Ⓡ^ plus Adjustable Volume Pipettes (Eppendorf, Catalog #: EP-START) 5. NanoDropTM 2000 Spectrophotometer (Thermo Fisher, model: NanoDropTM 2000, catalog number: ND-2000) 6. LightCycler^Ⓡ^ 96 System (Roche, catalog number: 05815916001) 7. Eppendorf Centrifuge 5417R (Catalog #:EP-LABKIT) 8. Eppendorf Mastercycler nexus (Eppendorf, Reference catalogue: 6336000015) 9. Tanon Gel Image System (Tanon, model: 2500) 10. Tanon EPS 300 (Tanon, model: 300) 11. Electrophoresis system (Product code: EL1820) 12. Horizontal laminar airflow cabinet (Cod: 14595 Description: HELIOS 36) 13. Deep freezer 14. Incubator (WEEE-Reg.-No. DE 66812464, ECCN Number: EAR99) 15. Spectrophotometer (Model 6305 UV-Visible)
	16. Vortex Mixer (Model Number: K-MI0101002D) 17. pH meter (PHS-3D-01) 18. Eppendorf MiniSpin Plus Microcentrifuge (CAT# 5453) 19. Precision balance (Cat # W3200-320) Software Bioinformatic softwares used for the development of specific primers. 1. Genome sequences were retrieved from NCBI GenBank (http://www.ncbi.nlm.nih.gov/genome/) [1] . 2. Coding sequences for housekeeping genes were manually extracted with the Artemis genome browser (http://www.sanger.ac.uk/science/tools/artemis) [2] 3. Sequence alignments were performed using the MUSCLE algorithm (https://www.ebi.ac.uk/Tools/msa/muscle/) [3]. 4. LAMP primers were designed using Primer Explorer V5 (http://primerexplorer.jp/lampv5e/index.html, Eiken Chemical Co., Japan). 5. Primer specificities were tested using Primer-BLAST at the NCBI website (https://www.ncbi.nlm.nih.gov/tools/primer-blast/) [4].
Experimental design:	Two diagnostic tools developed: one that detects all known species of *Pantoea* and the other one that is specific for *P. ananatis*. These tools will help monitor the phytosanitary status of rice seeds to avoid the dissemination of *Pantoea* spp.
Trial registration:	N/A
Ethics:	N/A
Value of the Protocol:	• Allow rapid and sensitive detection of these bacteria. • Help plant protection services for monitoring the phytosanitary status of rice seed in routine field and laboratory tests.

**Description of protocol**

## Background

The *Pantoea* genus is composed of several species characterized by their ubiquity and versatility in their interactions with living organisms and the environment [5], [6], [7]. Five species, namely *P. ananatis, P. agglomerans, P. stewartii, P. allii* and *P. wallisii*, have been widely reported as plant pathogens [8], [9], [10], [11] with *P. ananatis, P. agglomerans, P. stewartii* on rice plants [12,13] and seeds [14] in Sub-Saharan Africa. Collectively, they cause various diseases and symptoms, such as leaf blight and dieback, red streak, black spot, necrosis, bacterial spot, tumors, rot center, stem necrosis, leaf rot, decay of seed and Stewart's wilt [11].

*Pantoea* spp. are seed-transmitted and thus pose a significant threat to interstate and continental seed exchanges [7,^15^,16]. Thus, measures need to be taken to limit the pathogens' dispersion and robust diagnostic tools are required for rapid and cheap diagnosis that can be used in the field as well as in routine screening of seed lots.

New diagnostic tools have been developed, such as simplex and multiplex PCR schemes able of identifying the three major *Pantoea* species affecting rice plants [12]. A semi-selective medium for their isolation and identification has also been developed [13]. However, their use is complex, time consuming, expensive and cannot be deployed outside the laboratory.

Isothermal loop-mediated amplification (LAMP) is a relatively simple technique that facilitates rapid amplification of DNA and has a high level of sensitivity and specificity at low cost [17,18]. The technique uses the thermostable *Bst* DNA polymerase for reliable and fast polymerization and operates under isothermal conditions at relatively high temperature [19,20]. The results of LAMP reactions can be detected without electrophoretic separation but by visualization of turbidity, fluorescence or a color change thanks to a metal ion indicator [17]. LAMP has several advantages, such as portability, simplicity and cost-efficiency because the reaction can be carried out in a water bath or heating block. It has been used for highly specific and sensitive DNA amplification to detect various pathogens, including viruses, bacteria, protozoa and fungi [21]. However, LAMP has so far not been applied to rice/*Pantoea* spp., neglected pathosystems that are wide-spread and important in Africa (12, 14).

The goal of this work was to overcome the above difficulties and facilitate a quick and cost-effective diagnosis of the bacteria in rice leaves and paddy fields. Two diagnostic tools have been therefore developed: one that detects all known *Pantoea* species and another one that is specific for *P.* *ananatis*. These tools will help monitor the phytosanitary status of rice seeds to avoid the dissemination of *Pantoea* spp.

## Procedure

### Preparation of LAMP template (bacterial ooze, bacterial lyse and DNA) from diseased leaves and apparently healthy or symptomatic rice seeds

A. Collection of diseased leaves and grains

1. Collect diseased rice leaves at heading to maturity approaching stages and showing or not typical bacterial leaf blight symptoms or apparently healthy/symptomatic rice seed and put them into paper envelopes. Stored seeds can also be used.
2. Label the envelope by explaining variety, location, sampling date, rice ecosystem, take the samples to the laboratory and keep them in the refrigerator.

B. Preparation of bacterial ooze, bacterial lysate and genomic DNA

B-1. Bacterial ooze

1. Clean and air dry the diseased leaves and grains (NB: cut the leaves into small pieces) and sterilize them with a 1% sodium hypochlorite solution, then wash them with sterilized distilled water.
2. Put them into conical 15-ml polypropylene falcon tubes containing sterilized distilled water (5–10 ml) for about 10 to 15 min to allow the bacteria to ooze out from the leaf tissue.
3. Seal the tubes with as shown on the envelopes.
  pparafilm. parafilm Tubes containing leaf pieces/seeds in the acqueous solution are labelled with the same information Bacterial ooze that came out from the tissue is ready for use in LAMP tests, or

B-2. Bacterial lysate

4. Bring the tubes (in a cooler box containing ice) to the lab and streak the aqueous solution of each test tube on freshly prepared PSA or PGSA agar plates, label and incubate them at 28 °C for 1–3 days.
5. Then, scrape off the cells from the agar plates and suspend them in nuclease-free water previously distributed in LightCycler^Ⓡ^ 480 Multiwell Plate 96 or LightCycler^Ⓡ^ 8-Tube Strips.
6. Incubate the LightCycler^Ⓡ^ 480 Multiwell Plate 96 or the LightCycler^Ⓡ^ 8-Tube Strips at 95 °C for 45 min for bacterial lysis.
7. Use strains of *Pantoea* (CFBP 466, CFBP 3612^T^, CFBP 3171^PT^, CFBP 3615^PT^, CFBP3845^T^, CFBP 6627^T^) and *Erwinia* (CFBP 6632) as reference strains. Also include other rice pathogenic bacteria such as *Burkholderia glumae, Pseudomonas* spp., *Sphingomonas* spp., *Xanthomonas oryzae* as negative controls.

B-3. Genomic DNA

8. Extract the genomic DNA using the Wizard Genomic DNA Purification Kit according to the manufacturer's instructions. Then, evaluate the DNA quality and quantity by agarose gel electrophoresis and spectrophotometry.

### Loop-mediated isothermal amplification (LAMP) reaction

NB: Use either colorimetric LAMP or regular LAMP reactions

C. **Colorimetric LAMP reaction**

1. To simplify the assays, you can prepare in nuclease-free water all the six LAMP primers in Table 1 stored at –20 °C stocks (F3 and B3 outer primers, 2 µM; FIP and BIP inner primers, 16 µM; F-Loop and B-Loop primers, 4 µM).

**Table 1:** Primer sets used for LAMP assays in the present study.

Target gene	Species	Name	Sequence (5′ - 3′)	Amplicon size with F3+B3 (bp)	Length
*atpD*	*Pantoea* sp.	F3	GCAGTAGAGATCGCCTCTA	291	19
		B3	GATAACGTTGGAGTCGGTC		19
		FIP (F1c+F2)	CCTTGGCGAACGGACACA GTCTAACTCGCAGGAACTG		37
		BIP (B1c+B2)	GTGGTGCGGGTGTAGGTAAA CGAGTAACCTGAGTGTTCAG		40
		F-Loop	AGGTCGATAACCTTGATGCC		20
		B-Loop	AACATGATGGAACTGATCCGT		21
*gyrB*	*P. ananatis*	F3	GAGATACCGATGCAACCG	280	18
		B3	CCAATGCCGTCTTTCTCG		18
		FIP (F1c+F2)	CAGACGAATCGATACGCCAGA TCGAATACGACATTCTGGC		41
		BIP (B1c+B2)	GCGTGATGCAAGAAACGACC TTCAGGTACTCAACAAAGGC		40
		F-Loop	TTCAGGAAGGACAGTTCGC		19
		B-Loop	CACTACGAAGGTGGTATCCG		20
2. Prepare the reaction mix from 12.5 µl of WarmStart^Ⓡ^ Colorimetric LAMP 2X Master Mix, 2.5 µl of pre-mixed LAMP primers and 9 µl of nuclease-free water (Table 2), vortex briefly and centrifuge the reaction mix.

**Table 2:** Components of the colorimetric LAMP reaction

LAMP Component	Volume per reaction (µL)
WarmStart^Ⓡ^ Colorimetric LAMP 2X Master Mix	12.5 µl
LAMP Primer Mix (10X)	2.5 µl
Template (genomic DNA, heat-killed bacterial cells, seed extract or plant tissue exudate)	1 µl
Nuclease-free water	9 µl
Total volume	25 µl
3. Then, add 1 µl of one of the target templates (20 ng/µl genomic DNA, heat-killed bacterial cells or bacterial ooze from leaf tissue or rice seeds) to the 24-µl reagent mixes. Seal, vortex and centrifuge the reaction tubes that will provide a solution of bright pink color, which indicates the initial high pH required for successful LAMP reaction.
4. Incubate the reaction tubes at 65 °C for 30 min and examine by eye, with positive reactions turning into yellow while negative controls remaining pink.
5. In case of an orange color suggestive of a positive reaction, return the tubes to 65 °C for an additional 10 min. In order to intensify the color of positive reactions, cool the reaction tubes to room temperature.
6. Finally, take a photography of the tubes to record the colorimetric result.

D. **Regular LAMP reaction**

1. Carry out the LAMP reaction in a 25 µl volume containing 0.768 µl of each of the outer primers (F3 and B3; 1 µM) and the inner primers (FIP and BIP; 10 µM), 0.384 µl of each of the loop primers (FLoop and B-Loop; 10 µM), 3.5 µl of dNTPs (10 mM), 1.5 µl of MgSO_4_ (100 mM), 4 µl of betaine (5 M), 1 µl of *Bst* 2.0 DNA polymerase (8 U/µl), 2.5 µl of Isothermal Amplification Buffer (10X), 7.66 µl of nuclease-free water (Table 3).

**Table 3:** Composition of the regular LAMP reaction.

LAMP component	Volume per reaction (µL)
*Bst* 2.0 (New England Biolabs) 8 U/µL	1
Isothermal Amplification Buffer (New England Biolabs) 10X	2.5
Betaine (Sigma Aldrich) 5 M	4
dNTPs (10 mM)	3.5
MgSO_4_ (100 mM)	1.5
Outer primers (F3 and B3; 1 µM)	0.768 (each)
Inner primers (FIP and BIP; 10 µM)	0.768 (each)
Loop primers (Loop F and Loop B; 10 µM)	0.384 (each)
Nuclease-free water	7.66
Template (genomic DNA, heat-killed bacterial cells, seed extract or plant tissue exudate)	1
**Total**	25
Mineral oil	20
Quant-IT™ Pico Green^Ⓡ^ Reagent (Invitrogen, Carlsbad, CA, USA)	0.5
2. Add 1 µl of the template from one of the four types of templates: genomic DNA (20 ng/µl), heat-killed bacterial cells, the bacterial ooze from the leaf tissue or rice seeds.
3. Use 20 µl of mineral oil to cover the surface of the LAMP mixture in each reaction.

### Data analysis

**Fig. 1:**
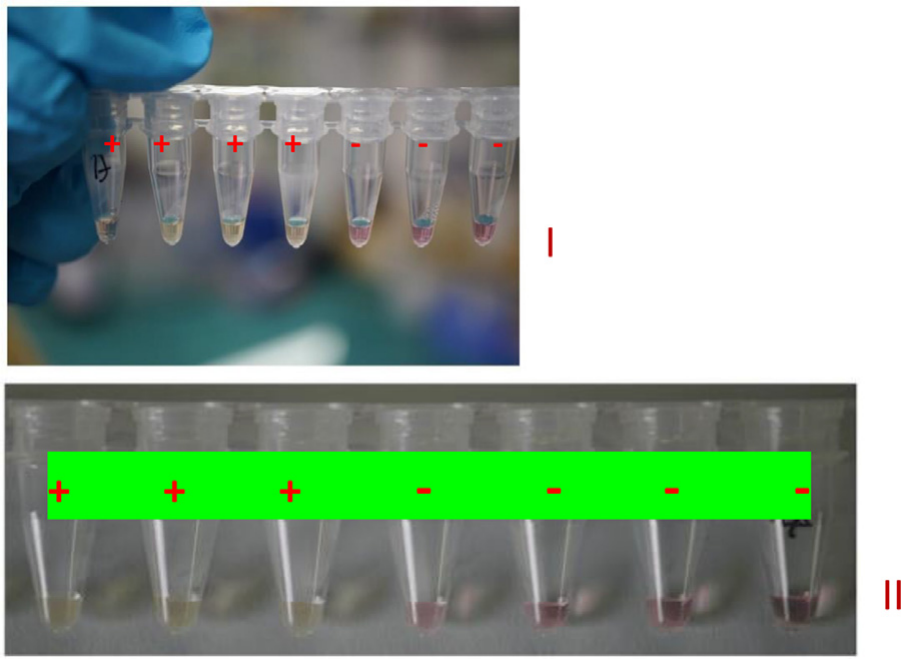
Colorimetric LAMP assay results using bacterial lysates, with *Pantoea* spp. (I) and *P. ananatis* (II) diagnostics tools. Negative (-) reactions are indicated in pink and positive (+) reactions are indicated by a change to yellow.

For the colorimetric LAMP assay, the results can be judged by naked eye. Negative (-) reactions remain pink while positive (+) reactions change to yellow. (Fig. 1).

**Fig. 2:**
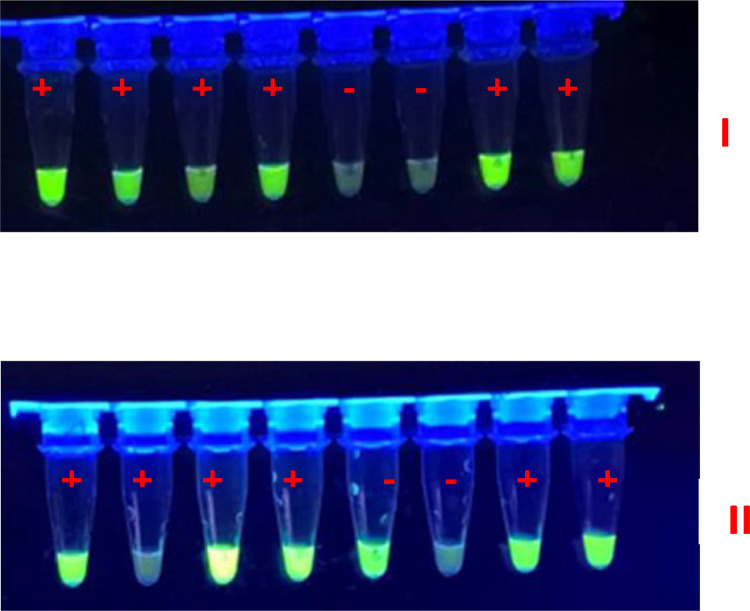
Regular LAMP assay results using bacterial lysates, with *Pantoea* spp. (I) and *P. ananatis* (II) diagnostics tools. Green fluorescent tubes are positive (+) while the others are negative (-).

For the regular detection of LAMP products, reaction tubes were placed under UV light. Positive reactions, i.e. DNA amplification, were identified by the bright green fluorescent color and could be seen by naked eye (Fig. 2). Green fluorescent tubes are positive (+) while the others are negative (-).

### Notes

1. The LAMP reaction is very sensitive. Attention should be paid to avoid contamination during the operation, and a stringent laboratory compartmentalization is strongly recommended for LAMP and other amplification assays.
2. To prevent cross-contamination, different sets of pipettes and different work areas must be used for DNA template preparation, PCR mixture preparation and DNA amplification. Gloves must be changed regularly and sterile pipetting techniques must be applied during the entire LAMP experiment.
3. To avoid potential contamination, agarose gel electrophoresis of LAMP products is not encouraged.
4. For bacterial colonies (lysates), the detection limit of the two LAMP assays was 10^4^ colony-forming units/ml. Using purified DNA, the thresholds were 0.5 fg for the *Pantoea*-genus-specific tool and 50 fg for the *P. ananatis*-specific tool.

### Recipes

• Peptone sucrose agar (PSA):

To 1 l of sterile distilled water, add 10 g peptone, 10 g sucrose, 16 g agar, and 1 g glutamic acid. Adjust pH to 7.1  ±  0.2 using 1 M KOH or NaOH solution. Autoclave at 121 °C for 20 min. Cool the bottle but let not solidify the medium, then pour into Petri dishes.

• *Pantoea* genus-specific agar (PGSA).

To 1 l of sterile distilled water (pH 7.1 ± 0.2); add 65 g NaCl; 0.001 g crystal violet; 8.5 g sodium thiosulfate; 13.5 g agar; 10 g peptone; and 10 g sucrose. Autoclave at 121 °C for 20 min. Cool the bottle but don't let to solidify the medium, then pour into Petri dishes.

• 1.5% agarose gel: 1.5 g of agarose in 100 ml of 1x TBE and 10 µl 10000x GelRed.
• Preparation of 1 M HCl

To prepare a solution of 1 M HCl, add 83 ml of concentrated HCl (37% w/w) to 1l of water.

• Preparation of 70% ethanol

Following the Gay-Lussac dilution table, add 40.85 ml of 96° ethanol to 100 ml of distilled sterile water.

## Declaration of Competing Interests

The authors declare that they have no known competing financial interests or personal relationships that could have appeared to influence the work reported in this paper.

## Appendix Supplementary materials

Image, application 1

Image, application 2

Image, application 3

Image, application 4

## References

[1] Benson D.A., Cavanaugh M., Clark K., Karsch-Mizrachi I., Ostell J., Pruitt K.D., Sayers E.W. (2018). GenBank. Nucleic Acids Res..

[2] Rutherford K., Parkhill J., Crook J., Horsnell T., Rice P., Rajandream M.-A., Barrell B. (2000). Artemis: sequence visualization and annotation. Bioinformatics.

[3] Edgar R.C. (2004). MUSCLE: multiple sequence alignment with high accuracy and high throughput. Nucleic Acids Res..

[4] Ye J., Coulouris G., Zaretskaya I., Cutcutache I., Rozen S., Madden T.L. (2012). Primer-BLAST: a tool to design target-specific primers for polymerase chain reaction. BMC Bioinform..

[5] Walterson A.M., Stavrinides J. (2015). Pantoea: insights into a highly versatile and diverse genus within the *Enterobacteriaceae*. FEMS Microbiol. Rev..

[6] Weller-Stuart T., De Maayer P., Coutinho T. (2017). *Pantoea ananatis*: genomic insights into a versatile pathogen. Molecular Plant Pathol..

[7] Völksch B., Thon S., Jacobsen I.D., Gube M. (2009). Polyphasic study of plant-and clinic-associated *Pantoea agglomerans* strains reveals indistinguishable virulence potential, Infection. Genet. Evol..

[8] Brady C.L., Venter S.N., Cleenwerck I., Engelbeen K., Vancanneyt M., Swings J., Coutinho T.A. (2009). *Pantoea vagans* sp. nov., *Pantoea eucalypti* sp. nov., *Pantoea deleyi* sp. nov. and *Pantoea anthophila* sp. nov.. Int. J. Syst. Evol. Microbiol..

[9] Brady C.L., Goszczynska T., Venter S.N., Cleenwerck I., De Vos P., Gitaitis R.D., Coutinho T.A. (2011). *Pantoea allii* sp. nov., isolated from onion plants and seed. Int. J. Syst. Evol. Microbiol..

[10] Delétoile A., Decré D., Courant S., Passet V., Audo J., Grimont P., Arlet G., Brisse S. (2009). Phylogeny and identification of *Pantoea* species and typing of *Pantoea agglomerans* strains by multilocus gene sequencing. J. Clin. Microbiol..

[11] Doni F., Suhaimi N.S.M., Mohamed Z., Ishak N., Mispan M.S. (2019). *Pantoea*: a newly identified causative agent for leaf blight disease in rice. J. Plant Dis. Protection.

[12] Kini K., Agnimonhan R., Dossa R., Silué D., Koebnik R. (2018). A diagnostic multiplex PCR scheme for identification of plant-associated bacteria of the genus *Pantoea*. bioRxiv.

[13] Kini K., Dossa R., Dossou B., Mariko M., Koebnik R., Silué D. (2019). A semi-selective medium to isolate and identify bacteria of the genus *Pantoea*. J. Gen. Plant Pathol..

[14] Dossou B., Silue D. (2018). Rice pathogens intercepted on seeds originating from 11 African countries and from the USA. Seed Sci. Technol..

[15] Walcott R.R., Gitaitis R.D., Castro A.C., Sanders F.H., Diaz-Perez J.C. (2002). Natural infestation of onion seed by *Pantoea ananatis*, causal agent of center rot. Plant Dis..

[16] Goszczynska T., Moloto V.M., Venter S.N., Coutinho T.A. (2006). Isolation and identification of *Pantoea ananatis* from onion seed in South Africa. Seed Sci. Technol..

[17] Mori Y., Notomi T. (2009). Loop-mediated isothermal amplification (LAMP): a rapid, accurate, and cost-effective diagnostic method for infectious diseases. J. Infect. Chemother..

[18] Notomi T., Okayama H., Masubuchi H., Yonekawa T., Watanabe K., Amino N., Hase T. (2000). Loop-mediated isothermal amplification of DNA. Nucleic Acids Res..

[19] Hafner G.J., Yang I.C., Wolter L.C., Stafford M.R., Giffard P.M. (2001). Isothermal amplification and multimerization of DNA by Bst DNA polymerase. BioTechniques.

[20] Mead D.A., McClary J.A., Luckey J.A., Kostichka A.J., Witney F.R., Smith L.M. (1991). Bst DNA polymerase permits rapid sequence analysis from nanogram amounts of template. BioTechniques.

[21] Niessen L. (2015). Current state and future perspectives of loop-mediated isothermal amplification (LAMP)-based diagnosis of filamentous fungi and yeasts. Appl. Microbiol. Biotechnol..

