# Supplementary material for: Development of two loop-mediated isothermal amplification (LAMP) genomics-informed diagnostic protocols for rapid detection of Pantoea species on rice
Source: MethodsX. 2021 Jan 6;8:101216. doi: 10.1016/j.mex.2021.101216 (PMC8374213; doi:10.1016/j.mex.2021.101216)
Supplement: Supplementary file 1 [file mmc1.docx]

PANST_CCUG26359{T} ATGTCGAATTCTTATGACTCTTCAAGTATCAAAGTTCTGAAAGGGCTTGATGCGGTACGC

PANST_LMG2632{PT} ATGTCGAATTCTTATGACTCTTCAAGTATCAAAGTTCTGAAAGGACTTGATGCGGTACGC

PANAL_LMG24248{T} ATGTCGAATTCTTATGACTCTTCAAGTATCAAAGTCCTGAAAGGGCTTGATGCGGTACGC

PANAN_LMG2665{T} ATGTCGAATTCTTATGACTCTTCAAGTATCAAAGTCCTGAAAGGGCTTGATGCGGTACGC

PANAN_97-1 ATGTCGAATTCTTATGACTCTTCAAGTATCAAAGTCCTGAAAGGGCTTGATGCGGTACGC

PANAN_LMG5342 ATGTCGAATTCTTATGACTCTTCAAGTATCAAAGTCCTGAAAGGGCTTGATGCGGTACGC

PANAN_LMG20103 ATGTCGAATTCTTATGACTCTTCAAGTATCAAAGTCCTGAAAGGGCTTGATGCGGTACGC

PANAN_NN08200 ATGTCGAATTCTTATGACTCTTCAAGTATCAAAGTCCTGAAAGGGCTTGATGCGGTACGC

PANAN_ARC311 ATGTCGAATTCTTATGACTCTTCAAGTATCAAAGTCCTGAAAGGGCTTGATGCGGTACGC

PANAN_RSA47 ATGTCGAATTCTTATGACTCTTCAAGTATCAAAGTCCTGAAAGGGCTTGATGCGGTACGC

PANAN_SGAir0210 ATGTCGAATTCTTATGACTCTTCAAGTATCAAAGTCCTGAAAGGGCTTGATGCGGTACGC

MIXCA_DSM22759{T} ATGTCGAATTCTTATGACTCCTCAAGTATCAAAGTCCTGAAAGGGCTTGATGCGGTACGC

MIXGA_DSM22758{T} ATGTCGAATTCTTATGACTCCTCAAGTATCAAAGTCCTGAAGGGGCTTGATGCGGTACGC

MIXAL_LTYR-11Z{T} ATGTCGAATTCTTATGACTCCTCAAGTATCAAAGTCCTGAAAGGACTTGATGCGGTACGC

MIXTH_QC88-366{T} ATGTCGAATTCTTATGACTCCTCAAGTATCAAAGTCCTGAAAGGGCTTGATGCGGTACGC

PANRO_LMG26273{T} ATGTCGAATTCTTATGACTCCTCAAGTATCAAAGTTCTTAAAGGGCTTGATGCGGTACGC

PANRW_LMG26275{T} ATGTCGAATTCTTATGACTCCTCAAGTATCAAAGTTCTTAAAGGGCTTGATGCGGTACGC

PANCY_LMG2657{T} ATGTCGAATTCTTATGACTCCTCAAGTATCAAAGTTCTGAAAGGGCTTGATGCGGTACGC

PANEU_LMG5346{T} ATGTCGAATTCTTATGACTCCTCAAGTATCAAAGTCCTGAAAGGGCTTGATGCGGTACGC

PANWA_LMG26277{T} ATGTCGAATTCTTATGACTCCTCAAGTATCAAAGTCCTGAAAGGACTTGATGCGGTACGC

PANDI_CCUG25232{T} ATGTCGAATTCTTATGACTCCTCAAGTATCAAAGTCCTGAAAGGGCTTGATGCGGTACGC

PANSE_LMG5345{T} ATGTCGAATTCTTATGACTCCTCAAGTATCAAAGTTCTGAAAGGGCTGGATGCGGTACGC

PANBR_LMG5343{T} ATGTCGAATTCTTATGACTCCTCAAGTATCAAAGTCCTGAAGGGGCTTGATGCGGTACGC

PANCO_LMG24534{T} ATGTCGAATTCTTATGACTCCTCAAGTATCAAAGTCCTGAAGGGGCTTGATGCGGTACGC

PANAN_LMG2558{T} ATGTCGAATTCTTATGACTCCTCCAGTATTAAAGTCCTGAAGGGGCTTGATGCGGTACGT

PANDE_LMG24200{T} ATGTCGAATTCTTATGACTCCTCAAGTATTAAAGTCCTGAAAGGACTTGATGCGGTACGC

PANVA_LMG24199{T} ATGTCGAATTCTTATGACTCCTCAAGTATTAAAGTCCTGAAAGGACTTGATGCGGTACGC

PANEU_LMG24197{T} ATGTCGAATTCTTATGACTCCTCAAGTATTAAAGTCCTGAAAGGACTTGATGCGGTACGC

PANAG_DSM3493{T} ATGTCGAATTCTTATGACTCCTCAAGTATTAAAGTCCTGAAAGGACTTGATGCGGTACGC

PANAG_CFBP13505 ATGTCGAATTCTTATGACTCCTCAAGTATTAAAGTCCTGAAAGGACTTGATGCGGTACGC

TATCI_DSM13699{T} ATGTCGAATTCTTATGACTCCTCAAGTATCAAAGTTCTGAAAGGACTTGATGCGGTACGC

TATMO_LMG23360{T} ATGTCGAATTCTTATGACTCCTCCAGTATCAAAGTTCTGAAAGGACTTGATGCGGTACGT

TATPT_ATCC33301{T} ATGTCGAATTCTTATGACTCCTCCAGTATCAAAGTCCTGAAAGGACTTGATGCGGTACGC

TATSA_NML06-3099{T} ATGTCGAATTCTTATGACTCCTCCAGTATCAAAGTCCTGAAAGGGCTTGATGCGGTACGC

******************** ** ***** ***** ** ** ** ** ***********

PANST_CCUG26359{T} AAACGCCCGGGCATGTACATCGGCGATACCGATGACGGAACCGGTCTGCATCACATGGTA

PANST_LMG2632{PT} AAACGCCCGGGCATGTACATCGGCGATACCGATGACGGAACCGGTCTGCATCACATGGTA

PANAL_LMG24248{T} AAACGCCCGGGCATGTACATCGGCGATACCGATGACGGAACCGGTCTGCATCACATGGTA

PANAN_LMG2665{T} AAACGCCCGGGCATGTACATCGGCGATACCGATGACGGAACCGGTCTGCATCACATGGTA

PANAN_97-1 AAACGCCCGGGCATGTACATCGGCGATACCGATGACGGAACCGGTCTGCATCACATGGTA

PANAN_LMG5342 AAACGCCCGGGCATGTACATCGGCGATACCGATGACGGAACCGGTCTGCATCACATGGTA

PANAN_LMG20103 AAACGCCCGGGCATGTACATCGGCGATACCGATGACGGAACCGGTCTGCATCACATGGTA

PANAN_NN08200 AAACGCCCGGGCATGTACATCGGCGATACCGATGACGGAACCGGTCTGCATCACATGGTA

PANAN_ARC311 AAACGCCCGGGCATGTACATCGGCGATACCGATGACGGAACCGGTCTGCATCACATGGTA

PANAN_RSA47 AAACGCCCGGGCATGTACATCGGCGATACCGATGACGGAACCGGTCTGCATCACATGGTA

PANAN_SGAir0210 AAACGCCCGGGCATGTACATCGGCGATACCGATGACGGAACCGGTCTGCATCACATGGTA

MIXCA_DSM22759{T} AAACGCCCGGGAATGTATATCGGCGATACGGATGACGGCACCGGTCTGCATCACATGGTA

MIXGA_DSM22758{T} AAACGCCCGGA-ATGTATATCGGCGATACGGATGACGGCACCGGTCTGCATCACATGGTA

MIXAL_LTYR-11Z{T} AAACGCCCGGGAATGTATATCGGCGATACGGATGACGGCACCGGTCTGCATCACATGGTA

MIXTH_QC88-366{T} AAACGCCCGGGAATGTATATCGGCGATACGGATGACGGCACCGGTCTGCATCACATGGTA

PANRO_LMG26273{T} AAACGCCCGGGTATGTATATCGGCGATACGGATGACGGCACCGGTCTGCATCACATGGTA

PANRW_LMG26275{T} AAACGCCCGGGCATGTACATCGGCGATACGGATGACGGCACCGGTCTGCATCACATGGTA

PANCY_LMG2657{T} AAACGCCCTGGCATGTATATCGGCGATACGGATGACGGCACCGGTCTGCATCACATGGTA

PANEU_LMG5346{T} AAACGCCCGGGTATGTATATCGGCGATACGGATGACGGCACCGGTCTGCATCACATGGTA

PANWA_LMG26277{T} AAACGCCCGGGTATGTACATCGGCGATACGGATGACGGCACCGGTCTGCATCACATGGTA

PANDI_CCUG25232{T} AAACGCCCGGGTATGTACATCGGCGATACGGATGACGGCACCGGTCTGCATCACATGGTA

PANSE_LMG5345{T} AAACGTCCGGGTATGTATATCGGCGATACGGATGACGGCACCGGTCTGCACCACATGGTA

PANBR_LMG5343{T} AAACGCCCGGGCATGTATATCGGCGATACCGATGACGGTACCGGTCTGCATCACATGGTA

PANCO_LMG24534{T} AAACGCCCGGGCATGTATATCGGCGATACCGATGACGGTACCGGTCTGCATCACATGGTA

PANAN_LMG2558{T} AAACGCCCGGGCATGTATATCGGCGATACCGATGACGGTACCGGTCTGCATCACATGGTA

PANDE_LMG24200{T} AAACGCCCGGGCATGTATATCGGCGATACCGATGACGGTACCGGTCTGCATCACATGGTA

PANVA_LMG24199{T} AAACGCCCGGGCATGTATATCGGCGATACCGATGACGGTACCGGTCTGCATCACATGGTA

PANEU_LMG24197{T} AAACGCCCGGGCATGTATATCGGCGATACCGATGACGGTACCGGTCTGCATCACATGGTA

PANAG_DSM3493{T} AAACGCCCGGGCATGTATATCGGCGATACCGATGACGGTACCGGTCTGCATCACATGGTA

PANAG_CFBP13505 AAACGCCCGGGCATGTATATCGGCGATACCGATGACGGTACCGGTCTGCATCACATGGTA

TATCI_DSM13699{T} AAACGCCCTGGTATGTACATCGGTGATACGGATGACGGCACAGGGTTGCATCACATGGTA

TATMO_LMG23360{T} AAACGCCCCGGTATGTACATCGGTGACACGGATGACGGCACCGGGTTGCATCACATGGTA

TATPT_ATCC33301{T} AAACGCCCCGGTATGTACATCGGTGACACGGATGACGGTACCGGTCTGCATCACATGGTA

TATSA_NML06-3099{T} AAACGCCCCGGTATGTACATCGGTGATACGGATGATGGTACCGGATTGCATCACATGGTA

***** ** * ***** ***** ** ** ***** ** ** ** **** *********

PANST_CCUG26359{T} TTCGAGGTCGTGGATAACGCAATCGACGAAGCGCTCGCCGGTCACTGTAAAGATATCGTT

PANST_LMG2632{PT} TTCGAGGTCGTGGATAACGCAATCGACGAAGCGCTCGCCGGTCACTGTAAAGATATCGTT

PANAL_LMG24248{T} TTCGAGGTCGTGGATAACGCAATCGACGAAGCGCTCGCCGGTCACTGTAAAGATATCGTC

PANAN_LMG2665{T} TTCGAGGTCGTGGATAACGCAATCGACGAAGCGCTCGCCGGTCACTGTAAAGATATTGTC

PANAN_97-1 TTCGAGGTCGTGGATAACGCAATCGACGAAGCGCTCGCCGGTCACTGTAAAGATATTGTC

PANAN_LMG5342 TTCGAGGTCGTGGATAACGCAATCGACGAAGCGCTCGCCGGTCACTGTAAAGATATTGTC

PANAN_LMG20103 TTCGAGGTCGTGGATAACGCAATCGACGAAGCGCTCGCCGGTCACTGTAAAGATATTGTC

PANAN_NN08200 TTCGAGGTCGTGGATAACGCAATCGACGAAGCGCTCGCCGGTCACTGTAAAGATATTGTC

PANAN_ARC311 TTCGAGGTCGTGGATAACGCAATCGACGAAGCGCTCGCCGGTCACTGTAAAGATATTGTC

PANAN_RSA47 TTCGAGGTCGTGGATAACGCAATCGACGAAGCGCTCGCCGGTCACTGTAAAGATATTGTC

PANAN_SGAir0210 TTCGAGGTCGTGGATAACGCAATCGACGAAGCGCTCGCCGGTCACTGTAAAGATATTGTC

MIXCA_DSM22759{T} TTCGAGGTCGTGGACAACGCCATCGACGAAGCGCTCGCAGGCCACTGTAAAGAGATTGTC

MIXGA_DSM22758{T} TTCGAGGTCGTGGACAACGCCATCGACGAAGCGCTCGCAGGCCACTGTAAAGAGATTGTC

MIXAL_LTYR-11Z{T} TTCGAGGTCGTGGATAACGCCATCGACGAAGCGCTCGCAGGCCACTGTAAAGAGATTACC

MIXTH_QC88-366{T} TTCGAGGTCGTGGATAACGCCATCGACGAAGCGCTCGCCGGCCACTGTAAAGAGATCCTG

PANRO_LMG26273{T} TTCGAGGTCGTGGATAACGCAATCGACGAAGCGCTCGCGGGTCACTGTAGTGACATTATG

PANRW_LMG26275{T} TTCGAGGTCGTGGATAACGCAATCGACGAAGCGCTCGCGGGTCACTGTAGTGACATTGTA

PANCY_LMG2657{T} TTCGAGGTCGTGGATAACGCCATTGACGAAGCGCTCGCCGGTTACTGTAGTGACATTATG

PANEU_LMG5346{T} TTCGAGGTCGTGGATAACGCCATCGACGAAGCGCTCGCCGGACACTGCAGTGACATCGTG

PANWA_LMG26277{T} TTCGAGGTCGTGGATAACGCCATCGACGAAGCGCTCGCGGGTCACTGCAGTGACATCATG

PANDI_CCUG25232{T} TTCGAGGTCGTGGATAACGCCATCGACGAAGCGCTCGCCGGCCACTGTAGTGATATCGTG

PANSE_LMG5345{T} TTCGAGGTCGTGGATAACGCAATCGACGAAGCGCTCGCCGGTCACTGTAGTGATATCGTC

PANBR_LMG5343{T} TTCGAGGTCGTGGATAACGCAATCGACGAAGCGCTCGCTGGTCACTGTAGTGACATCGTG

PANCO_LMG24534{T} TTCGAGGTCGTGGATAACGCAATCGACGAAGCGCTCGCTGGTCACTGTAGTGACATCGTG

PANAN_LMG2558{T} TTCGAGGTCGTGGATAACGCAATCGACGAAGCGCTCGCTGGTCACTGTAGTGACATTGTG

PANDE_LMG24200{T} TTCGAGGTCGTGGATAACGCAATCGACGAAGCGCTCGCTGGTCACTGTAGTGACATTGTG

PANVA_LMG24199{T} TTCGAGGTCGTGGATAACGCAATCGACGAAGCGCTCGCTGGTCACTGTAGTGACATCGTA

PANEU_LMG24197{T} TTCGAGGTCGTGGATAACGCAATCGACGAAGCGCTCGCTGGTCACTGTAGTGACATTGTG

PANAG_DSM3493{T} TTCGAGGTCGTGGATAACGCAATCGACGAAGCGCTCGCTGGTCACTGTAGTGACATTGTG

PANAG_CFBP13505 TTCGAGGTCGTGGATAACGCAATCGACGAAGCGCTCGCTGGTCACTGTAGTGACATTGTG

TATCI_DSM13699{T} TTCGAGGTTGTGGACAACGCCATCGACGAAGCACTCGCGGGCTACTGTAAAGAAATTATT

TATMO_LMG23360{T} TTCGAGGTTGTGGATAACGCCATCGACGAAGCACTTGCGGGCCACTGCAAGGAAATTATT

TATPT_ATCC33301{T} TTCGAGGTAGTGGACAACGCCATCGACGAAGCACTCGCAGGCCACTGTAAAGAAATCACC

TATSA_NML06-3099{T} TTCGAGGTTGTGGACAACGCCATCGACGAAGCACTCGCAGGGCATTGTAAGGAAATCACC

******** ***** ***** ** ******** ** ** ** * ** * ** **

PANST_CCUG26359{T} GTCACCATCCATGCGGACAACTCGGTTTCCGTACAGGATGACGGACGCGGTATTCCAACC

PANST_LMG2632{PT} GTCACCATCCATGCGGACAACTCGGTTTCCGTACAGGATGACGGACGCGGTATTCCAACC

PANAL_LMG24248{T} GTCACCATCCATGCGGATAACTCTGTCTCCGTACAGGATGACGGACGTGGTATCCCAACC

PANAN_LMG2665{T} GTGACAATCCATGCCGATAATTCGGTTTCCGTTCAGGATGACGGACGCGGAATTCCAACC

PANAN_97-1 GTGACAATCCATGCCGATAATTCGGTTTCCGTTCAGGATGACGGACGCGGAATTCCTACC

PANAN_LMG5342 GTGACAATCCATGCCGATAATTCGGTTTCCGTTCAGGATGACGGACGCGGAATTCCTACC

PANAN_LMG20103 GTGACAATCCATGCCGATAATTCGGTTTCCGTTCAGGATGACGGACGCGGAATTCCTACC

PANAN_NN08200 GTGACAATCCATGCCGATAATTCGGTTTCCGTTCAGGATGACGGACGCGGAATTCCTACC

PANAN_ARC311 GTGACAATCCATGCCGATAATTCGGTTTCCGTTCAGGATGATGGACGCGGAATTCCAACC

PANAN_RSA47 GTGACAATCCATGCCGATAATTCGGTTTCAGTTCAGGATGATGGACGCGGAATTCCAACC

PANAN_SGAir0210 GTGACAATCCATGCCGATAATTCGGTTTCCGTTCAGGATGATGGACGCGGAATTCCAACC

MIXCA_DSM22759{T} GTGACTATCCATGCGGATAACTCCGTATCGGTGCAGGATGACGGCCGCGGCATTCCTACC

MIXGA_DSM22758{T} GTTACTATCCATGCCGATAACTCGGTCTCGGTGCAGGATGACGGACGCGGCATTCCGACC

MIXAL_LTYR-11Z{T} GTTACTATCCACGCTGATAATTCCGTATCGGTGCAGGATGATGGCCGTGGCATTCCTACC

MIXTH_QC88-366{T} GTTACCATTCATGCGGACAATTCCGTATCGGTGCAGGATGATGGCCGTGGCATTCCTACC

PANRO_LMG26273{T} GTCACTATTCATGCCGATAATTCTGTTTCAGTTCAAGATGATGGCCGTGGTATTCCGACC

PANRW_LMG26275{T} GTGACCATCCATGCGGACAATTCTGTGTCTGTTCAAGATGATGGCCGTGGTATTCCGACC

PANCY_LMG2657{T} GTGACCATCCATGCCGATAACTCGGTATCGGTGCAGGATGATGGCCGTGGTATCCCAACG

PANEU_LMG5346{T} GTCACTATCCACGCGGATAACTCCGTGTCGGTGCAGGATGACGGGCGCGGTATTCCAACC

PANWA_LMG26277{T} GTGACCATTCACGCCGACAATTCCGTTTCCGTACAGGATGATGGACGTGGTATTCCGACC

PANDI_CCUG25232{T} GTCACCATCCATGCCGACAACTCTGTCTCCGTACAGGATGACGGCCGTGGTATTCCGACC

PANSE_LMG5345{T} GTGACCATTCATGCCGATAATTCCGTTTCCGTTCAGGATGATGGCCGTGGCATTCCCACC

PANBR_LMG5343{T} GTCACCATTCATGCGGATAACTCCGTTTCGGTGCAGGATGATGGACGCGGCATTCCAACC

PANCO_LMG24534{T} GTCACCATTCATGCGGATAACTCCGTTTCGGTGCAGGATGATGGACGCGGCATTCCAACC

PANAN_LMG2558{T} GTCACCATCCATGCGGATAACTCCGTCTCGGTGCAGGATGATGGACGCGGCATTCCGACC

PANDE_LMG24200{T} GTCACCATCCATGCGGACAACTCCGTTTCGGTGCAGGATGATGGACGCGGCATTCCTACC

PANVA_LMG24199{T} GTCACTATCCATGCGGATAACTCCGTCTCGGTACAGGATGATGGACGCGGCATTCCTACC

PANEU_LMG24197{T} GTCACCATCCACGCGGATAACTCCGTTTCGGTGCAGGATGATGGACGCGGCATTCCAACC

PANAG_DSM3493{T} GTCACCATCCATGCCGATAACTCCGTATCGGTGCAGGATGATGGACGCGGCATTCCTACC

PANAG_CFBP13505 GTCACCATCCATGCCGATAACTCCGTATCGGTGCAGGATGATGGACGCGGCATTCCTACC

TATCI_DSM13699{T} GTTACCATTCATGCAGATAACTCTGTTTCTGTTCAGGATGATGGCCGTGGTATTCCTACC

TATMO_LMG23360{T} GTTACTATTCACGCAGATAACTCTGTGTCTGTTCAGGATGATGGCCGTGGAATTCCTACC

TATPT_ATCC33301{T} GTCACTATCCACTCCGATAACTCCGTCTCAGTTCAGGATGATGGTCGTGGTATTCCGACC

TATSA_NML06-3099{T} GTGACCATCCATTCTGACAACTCAGTGTCAGTACAGGATGATGGCCGTGGTATTCCGACC

** ** ** ** * ** ** ** ** ** ** ** ***** ** ** ** ** ** **

PANST_CCUG26359{T} GGTATCCACGAAGAAGAAGGCGTGTCCGCGGCAGAAGTGATCATGACCGTGCTGCACGCT

PANST_LMG2632{PT} GGTATCCACGAAGAAGAAGGCGTGTCCGCGGCAGAAGTGATCATGACGGTGCTGCACGCT

PANAL_LMG24248{T} GGCATCCACGAAGAAGAAGGTGTGTCGGCAGCGGAAGTCATCATGACCGTCCTGCACGCC

PANAN_LMG2665{T} GGTATCCACGAAGAAGAAGGTGTGTCGGCGGCTGAAGTTATCATGACCGTGCTGCACGCT

PANAN_97-1 GGTATCCACGAAGAAGAAGGTGTGTCGGCGGCTGAAGTTATCATGACCGTGCTGCACGCT

PANAN_LMG5342 GGTATCCACGAAGAAGAAGGTGTGTCGGCGGCTGAAGTTATCATGACCGTGCTGCACGCT

PANAN_LMG20103 GGTATCCACGAAGAAGAAGGTGTGTCGGCGGCTGAAGTTATCATGACCGTGCTGCACGCT

PANAN_NN08200 GGTATCCACGAAGAAGAAGGTGTGTCGGCGGCTGAAGTTATCATGACCGTGCTGCACGCT

PANAN_ARC311 GGTATCCACGAAGAAGAAGGTGTATCGGCGGCTGAAGTTATCATGACCGTACTGCACGCT

PANAN_RSA47 GGTATCCACGAAGAAGAAGGTGTATCGGCGGCTGAAGTTATCATGACCGTATTGCACGCT

PANAN_SGAir0210 GGTATCCACGAAGAAGAAGGTGTATCGGCGGCTGAAGTTATCATGACCGTACTGCACGCT

MIXCA_DSM22759{T} GGGATTCACCCGGAAGAAGGCGTGTCGGCGGCGGAAGTGATCATGACCGTGCTGCACGCC

MIXGA_DSM22758{T} GGCATCCACCCGGAAGAGGGCGTCTCGGCGGCGGAAGTGATCATGACCGTGCTGCACGCC

MIXAL_LTYR-11Z{T} GGGATCCACCCGGAGGAAGGCGTTTCGGCAGCGGAAGTGATCATGACCGTACTGCACGCG

MIXTH_QC88-366{T} GGCATCCACCCGGAAGAAGGCGTTTCGGCGGCGGAAGTGATCATGACCGTACTGCACGCC

PANRO_LMG26273{T} GGCATTCACCCGGAAGAGGGTGTTTCTGCAGCGGAAGTGATCATGACCGTGCTGCACGCT

PANRW_LMG26275{T} GGCATTCACCCGGAAGAGGGTGTGTCTGCGGCGGAAGTGATCATGACCGTCCTGCACGCC

PANCY_LMG2657{T} GGTATTCACCCGGAAGAGGGCGTATCGGCGGCTGAAGTCATCATGACGGTACTGCATGCG

PANEU_LMG5346{T} GGTATTCACCCGGAAGAGGGTGTCTCGGCGGCGGAAGTGATCATGACCGTTCTGCACGCC

PANWA_LMG26277{T} GGCATTCACCCGGAAGAGGGCGTCTCCGCGGCGGAAGTGATCATGACCGTCCTGCACGCC

PANDI_CCUG25232{T} GGTATCCACCCGGAAGAGGGCGTCTCTGCTGCCGAAGTGATCATGACCGTACTGCATGCC

PANSE_LMG5345{T} GGCATTCACGAAGAAGAGGGCGTTTCCGCTGCGGAAGTGATCATGACCGTGCTGCACGCA

PANBR_LMG5343{T} GGCATTCACGAAGAAGAAGGCATTTCCGCCGCTGAAGTGATCATGACGGTGCTGCATGCG

PANCO_LMG24534{T} GGCATTCACGAAGAAGAGGGCATTTCCGCCGCTGAAGTGATCATGACCGTGCTGCATGCC

PANAN_LMG2558{T} GGTATTCACGAAGAAGAGGGCGTGTCCGCCGCTGAAGTGATCATGACCGTTCTGCATGCG

PANDE_LMG24200{T} GGCATTCACGAAGAAGAGGGCGTTTCCGCCGCGGAAGTGATCATGACCGTGCTGCATGCG

PANVA_LMG24199{T} GGTATTCACGAAGAAGAGGGCGTTTCCGCCGCTGAAGTGATCATGACCGTGCTGCATGCG

PANEU_LMG24197{T} GGTATTCACGAAGAAGAGGGCATCTCTGCCGCTGAAGTGATCATGACCGTGCTGCATGCT

PANAG_DSM3493{T} GGTATTCACGAAGAAGAGGGCATTTCTGCCGCTGAAGTGATCATGACCGTGCTGCATGCT

PANAG_CFBP13505 GGTATTCACGAAGAAGAGGGCATCTCTGCCGCTGAAGTGATCATGACCGTGCTGCATGCT

TATCI_DSM13699{T} GGCATTCACCCTGAGGAAGGTGTTTCTGCTGCCGAAGTTATCATGACGGTACTGCATGCC

TATMO_LMG23360{T} GGTATTCACCCTGAGGAAGGTGTTTCTGCGGCAGAAGTTATCATGACGGTACTGCATGCC

TATPT_ATCC33301{T} GGAATTCACCCGGAAGAGGGCGTATCGGCGGCAGAAGTGATTATGACTGTGCTGCATGCC

TATSA_NML06-3099{T} GGTATCCACCCTGAAGAGGGAGTCTCGGCAGCCGAGGTCATCATGACCGTGCTGCATGCA

** ** *** ** ** ** * ** ** ** ** ** ** ***** ** **** **

PANST_CCUG26359{T} GGCGGTAAGTTCGACGATAACTCGTATAAAGTCTCGGGCGGCCTGCACGGCGTGGGCGTG

PANST_LMG2632{PT} GGCGGTAAGTTCGACGATAACTCGTATAAAGTCTCGGGCGGCCTGCACGGCGTGGGCGTG

PANAL_LMG24248{T} GGTGGTAAGTTTGATGACAACTCTTATAAAGTGTCTGGCGGCCTGCACGGCGTGGGTGTT

PANAN_LMG2665{T} GGCGGTAAGTTTGACGACAACTCCTACAAAGTGTCTGGCGGCCTGCACGGCGTAGGTGTT

PANAN_97-1 GGCGGTAAGTTTGACGACAACTCCTATAAAGTGTCTGGCGGCCTTCACGGCGTAGGTGTT

PANAN_LMG5342 GGCGGTAAGTTTGACGACAACTCCTATAAAGTGTCTGGCGGCCTGCACGGCGTAGGTGTT

PANAN_LMG20103 GGCGGTAAGTTTGACGACAACTCCTATAAAGTGTCTGGCGGCCTGCACGGCGTAGGTGTT

PANAN_NN08200 GGCGGTAAGTTTGACGACAACTCCTATAAAGTGTCTGGCGGCCTGCACGGCGTAGGTGTT

PANAN_ARC311 GGCGGTAAGTTTGACGACAACTCCTATAAAGTGTCTGGCGGCCTGCACGGCGTAGGTGTT

PANAN_RSA47 GGCGGTAAGTTTGACGACAACTCCTATAAAGTGTCTGGCGGCCTGCACGGCGTAGGTGTT

PANAN_SGAir0210 GGCGGTAAGTTTGACGACAACTCCTATAAAGTGTCTGGCGGCCTGCACGGCGTAGGTGTT

MIXCA_DSM22759{T} GGCGGTAAGTTCGACGACAACTCCTATAAAGTTTCCGGCGGCCTGCACGGTGTGGGCGTT

MIXGA_DSM22758{T} GGCGGCAAGTTCGACGACAACTCCTATAAAGTTTCCGGCGGCCTGCACGGCGTGGGCGTT

MIXAL_LTYR-11Z{T} GGCGGTAAGTTCGATGACAACTCCTACAAAGTCTCCGGCGGCCTGCATGGCGTGGGCGTT

MIXTH_QC88-366{T} GGCGGTAAGTTCGATGACAACTCCTATAAAGTTTCCGGCGGCCTGCACGGCGTAGGCGTC

PANRO_LMG26273{T} GGCGGTAAGTTCGACGATAACTCGTATAAAGTTTCTGGTGGTCTGCACGGCGTTGGCGTT

PANRW_LMG26275{T} GGCGGTAAATTCGACGATAACTCGTATAAAGTGTCCGGTGGTCTGCACGGCGTGGGTGTT

PANCY_LMG2657{T} GGCGGTAAGTTCGACGACAACTCCTATAAAGTTTCCGGTGGTCTGCACGGTGTGGGTGTC

PANEU_LMG5346{T} GGCGGTAAGTTTGATGACAACTCTTACAAAGTGTCGGGCGGCCTGCACGGCGTTGGCGTT

PANWA_LMG26277{T} GGCGGCAAGTTCGATGACAACTCCTATAAAGTTTCCGGCGGTCTGCACGGCGTGGGCGTC

PANDI_CCUG25232{T} GGCGGTAAGTTTGATGACAACTCTTATAAAGTTTCCGGTGGTCTGCACGGCGTCGGCGTA

PANSE_LMG5345{T} GGCGGTAAGTTCGACGACAACTCCTATAAAGTATCGGGCGGTCTGCACGGCGTAGGCGTT

PANBR_LMG5343{T} GGCGGTAAGTTCGACGATAACTCCTATAAAGTCTCCGGCGGTCTGCATGGCGTCGGTGTC

PANCO_LMG24534{T} GGCGGTAAGTTCGACGATAACTCCTATAAAGTCTCCGGCGGTCTGCATGGCGTCGGTGTC

PANAN_LMG2558{T} GGCGGTAAATTCGATGATAACTCCTACAAGGTCTCCGGCGGCCTGCACGGCGTGGGTGTC

PANDE_LMG24200{T} GGCGGTAAGTTCGACGATAACTCCTATAAAGTGTCCGGCGGCCTGCATGGCGTGGGTGTC

PANVA_LMG24199{T} GGCGGTAAGTTCGACGATAACTCCTATAAAGTCTCCGGCGGCCTGCATGGCGTGGGCGTC

PANEU_LMG24197{T} GGCGGTAAGTTCGATGATAACTCCTATAAAGTCTCCGGCGGCCTGCATGGCGTGGGCGTC

PANAG_DSM3493{T} GGCGGTAAGTTCGATGATAACTCCTATAAAGTCTCCGGCGGCCTGCACGGCGTGGGCGTC

PANAG_CFBP13505 GGCGGTAAGTTCGATGATAACTCCTATAAAGTCTCCGGCGGCCTGCACGGCGTGGGCGTC

TATCI_DSM13699{T} GGCGGTAAGTTTGATGATAACTCGTATAAAGTGTCAGGCGGCCTGCATGGTGTCGGTGTT

TATMO_LMG23360{T} GGCGGTAAGTTTGATGATAACTCGTACAAAGTGTCAGGCGGCCTGCACGGTGTTGGTGTT

TATPT_ATCC33301{T} GGGGGTAAGTTTGACGATAACTCTTATAAAGTGTCCGGTGGTTTACATGGCGTGGGTGTC

TATSA_NML06-3099{T} GGCGGTAAATTTGACGATAACTCCTATAAAGTGTCTGGTGGTCTGCACGGTGTCGGTGTC

** ** ** ** ** ** ***** ** ** ** ** ** ** * ** ** ** ** **

PANST_CCUG26359{T} TCTGTGGTTAACGCCCTGTCACAGAAGCTGGAGCTGACCATTCGTCGCGAAGGCAAAGTG

PANST_LMG2632{PT} TCTGTGGTTAACGCCCTGTCACAGAAGCTGGAGCTGACCATTCGTCGCGAAGGCAAAGTG

PANAL_LMG24248{T} TCCGTTGTTAACGCCCTGTCGCAGAAGCTGGAGCTGACCATTCGTCGCGAAGGCAAAGTG

PANAN_LMG2665{T} TCCGTTGTGAACGCCCTGTCCCAGAAACTGGAACTGACCATTCGTCGTGAAGGCAAAGTG

PANAN_97-1 TCCGTTGTGAACGCCCTGTCCCAGAAACTGGAACTGACCATTCGTCGTGAAGGCAAAGTA

PANAN_LMG5342 TCCGTTGTGAACGCCCTGTCCCAGAAACTGGAACTGACCATTCGTCGTGAAGGCAAAGTG

PANAN_LMG20103 TCCGTTGTGAACGCCCTGTCCCAGAAACTGGAACTGACCATTCGTCGTGAAGGCAAAGTG

PANAN_NN08200 TCCGTTGTGAACGCCCTGTCCCAGAAACTGGAACTGACCATTCGTCGTGAAGGCAAAGTG

PANAN_ARC311 TCCGTTGTTAACGCCCTGTCCCAGAAACTGGAACTGACCATTCGTCGTGAAGGCAAAGTG

PANAN_RSA47 TCCGTTGTTAACGCCCTGTCCCAGAAACTGGAACTGACCATTCGTCGTGAAGGCAAAGTG

PANAN_SGAir0210 TCCGTTGTTAACGCCCTGTCCCAGAAACTGGAACTGACCATTCGTCGTGAAGGCAAAGTG

MIXCA_DSM22759{T} TCTGTAGTTAACGCGCTGTCTGAAAAGCTGGAGCTGACTATTCGCCGCGAAGGCAAAGTG

MIXGA_DSM22758{T} TCCGTGGTGAATGCGCTCTCTGAAAAGCTGGAGCTGACCATTCGTCGCGAAGGCAAAGTG

MIXAL_LTYR-11Z{T} TCGGTAGTTAACGCGCTTTCTGAAAAGCTGGAACTGACCATTCGTCGCGAAGGTAAAGTG

MIXTH_QC88-366{T} TCGGTGGTTAACGCACTTTCTGAAAAGCTGGAGCTGACCATCCGTCGTGAAGGCAAAGTG

PANRO_LMG26273{T} TCAGTGGTAAACGCCCTGTCGCAGAAACTGGAGCTGACGATTCGTCGTGAAGGCAAAGTG

PANRW_LMG26275{T} TCAGTGGTTAACGCCTTGTCGCAGAAACTGGAGCTGACGATTCGCCGCGAAGGTAAAGTG

PANCY_LMG2657{T} TCCGTAGTAAACGCCCTGTCGCAGAAACTGGAACTGACCATTCGTCGCGATGGCAAAGTG

PANEU_LMG5346{T} TCCGTGGTTAATGCCCTGTCGCAGAAGCTGGAGCTGACCATTCGTCGCGACGGCAAAGTG

PANWA_LMG26277{T} TCCGTGGTTAACGCCCTGTCCCAGAAGCTGGAACTGACCATTCGTCGTGACGGTAAAGTG

PANDI_CCUG25232{T} TCGGTGGTGAACGCCCTGTCGCAGAAGCTGGAACTGACCATTCGTCGCGACGGCAAAGTG

PANSE_LMG5345{T} TCGGTCGTCAACGCCCTGTCGCAGAAGCTGGAGCTGACCATTCGTCGCGAAGGCAAAGTG

PANBR_LMG5343{T} TCGGTGGTTAACGCCCTGTCACAGAAGCTGGAGCTGACCATTCGTCGCGAAGGCAAAGTA

PANCO_LMG24534{T} TCGGTGGTAAACGCCCTGTCACAGAAGCTGGAGCTGACCATTCGTCGCGAAGGCAAAGTA

PANAN_LMG2558{T} TCGGTCGTTAACGCCCTGTCGCAGAAGCTGGAGCTGACCATTCGTCGCGAAGGCAAAGTG

PANDE_LMG24200{T} TCCGTTGTTAACGCCCTGTCACAGAAGCTGGAGCTGACCATCCGTCGCGAAGGCAAAGTG

PANVA_LMG24199{T} TCTGTCGTTAACGCCCTGTCGCAGAAGCTGGAGCTGACCATTCGTCGCGAAGGCAAAGTG

PANEU_LMG24197{T} TCCGTGGTTAACGCCCTGTCGCAGAAGCTGGAGCTGACCATTCGTCGCGAAGGCAAAGTG

PANAG_DSM3493{T} TCCGTGGTTAACGCCCTGTCGCAGAAGCTGGAGCTGACCATTCGTCGCGAAGGCAAAGTG

PANAG_CFBP13505 TCCGTGGTTAACGCCCTGTCGCAGAAGCTGGAGCTGACCATTCGTCGCGAAGGCAAAGTG

TATCI_DSM13699{T} TCGGTGGTGAATGCCTTATCCGAAAAACTGGAACTGACGATTCGTCGTGATGGTAAAGTC

TATMO_LMG23360{T} TCGGTGGTGAATGCCTTATCCGAAAAACTGGAACTGACGATTCGTCGTGATGGAAAAGTC

TATPT_ATCC33301{T} TCTGTCGTCAACGCATTGTCTGAAAAGCTGGAACTGACTATCCGTCGTGACGGTAAAGTT

TATSA_NML06-3099{T} TCTGTGGTGAATGCTCTGTCTGAAAAACTGGAGCTGACTATTCGCCGTGATGGTAAAGTC

** ** ** ** ** * ** * ** ***** ***** ** ** ** ** ** *****

**F3**

**GAGATACC**

PANST_CCUG26359{T} CACCAGCAGATTTATCTGCATGGCGTGCCCGAGGCGCCGCTGGCGGTGACAGGCGATACC

PANST_LMG2632{PT} CACCAGCAGATTTATCTGCATGGCGTGCCCGAGGCGCCGCTGGCGGTGACAGGCGATACC

PANAL_LMG24248{T} CATCAGCAGATCTATGTGCACGGGGTGCCTCAAGCGCCACTGGCGGCCACTGGCGATACC

PANAN_LMG2665{T} CATCAGCAGATTTATCTGCACGGCGTACCTGAAGCGCCGCTGGCGGTTACGGGAGATACC

PANAN_97-1 CACCAGCAGATTTATCTGCACGGCGTACCTGAAGCGCCGCTGGCGGTTACGGGAGATACC

PANAN_LMG5342 CACCAGCAGATTTATCTGCACGGCGTACCTGAAGCGCCGCTGGCGGTTACGGGAGATACC

PANAN_LMG20103 CATCAGCAGATTTATCTGCACGGCGTACCTGAAGCGCCGCTGGCGGTTACGGGAGATACC

PANAN_NN08200 CATCAGCAGATTTATCTGCATGGCGTACCTGAAGCGCCGCTGGCGGTTACGGGAGATACC

PANAN_ARC311 CATCAGCAGATTTATCTGCACGGCGTACCTGAAGCGCCGCTGGCGGTTACGGGAGATACC

PANAN_RSA47 CATCAGCAGATTTATCTGCACGGCGTGCCTGAAGCACCGCTGGCGATTACGGGAGATACC

PANAN_SGAir0210 CATCAGCAGATTTATCTGCACGGCGTGCCTGAAGCGCCGCTGGCGGTTACGGGAGATACC

MIXCA_DSM22759{T} CACCAGCAGACCTATGTTCACGGCGTGCCGCAGGCGCCGCTGGCGGTCACCGGCGAAACC

MIXGA_DSM22758{T} CATCAGCAGACCTATGTACACGGCGTGCCGCAGGCACCGCTGGCCGTGACCGGCGAAACC

MIXAL_LTYR-11Z{T} CATCAGCAGACCTATGTGCACGGCGTGCCGCAGGCACCGCTGGCGGTCACAGGCGAAACC

MIXTH_QC88-366{T} CATCAGCAGACCTATGTTCACGGCGTGCCGCAGGCGCCGCTGGCGGTTACGGGCGAAACC

PANRO_LMG26273{T} CACCAGCAGGTTTATCAGCACGGCGTGCCACAGGCACCGTTGGCGGTCTCCGGCGATACC

PANRW_LMG26275{T} CATCAGCAGATTTACCAGCACGGTGTGCCGCAGGCACCGCTTGCCGTATCCGGTGATACC

PANCY_LMG2657{T} CACCAGCAAACCTACATTCACGGCGTGCCGCAGGCACCGCTGGCCGTGGTCGGTGACACT

PANEU_LMG5346{T} CATCAGCAGACCTACGTGCACGGCGTGCCGCAGGCGCCGCTGGCGGTTACCGGCGATACC

PANWA_LMG26277{T} CATCAGCAGACTTACGTGCACGGCGTGCCGCAGGCTCCGCTGGCTGTGACCGGTGATACC

PANDI_CCUG25232{T} CATCAGCAAACCTACGTGCACGGTGTGCCGCAGGCGCCACTGGCGGTGACCGGCGATACC

PANSE_LMG5345{T} CACCAGCAGACTTACGTGCATGGCGTGCCTGAGGCACCGCTGAACGTCACCGGCGAAACC

PANBR_LMG5343{T} CACCAGCAGGTCTACGTCCATGGCGTGCCGCAGGCACCGCTGTCGGTGACCGGCGAGACC

PANCO_LMG24534{T} CACCAGCAGATCTACGTCCATGGCGTGCCGCAGGCACCGTTGTCGGTGACCGGCGAGACC

PANAN_LMG2558{T} CATCAGCAGATTTACGTCCACGGCGTTCCCGAGTCGCCGCTGACCGTGACCGGTGATACC

PANDE_LMG24200{T} CACCAGCAGATCTACGTCCACGGCGTCCCTGAGGCCCCGCTGGCGATCACGGGCGATACC

PANVA_LMG24199{T} CATCAGCAGATTTACGTCCACGGCGTACCTGAGGCACCGCTGGCGGTCACTGGTGATACC

PANEU_LMG24197{T} CATCAGCAGGTTTACGTCCATGGCGTACCGCAGGCACCGCTGGCGGTAACGGGTGAAACC

PANAG_DSM3493{T} CATCAGCAGATTTACGTCCATGGCGTACCGCAGGCACCACTGGCGGTGACGGGTGAGACC

PANAG_CFBP13505 CATCAGCAGATTTACGTCCATGGCGTACCGCAGGCACCGCTGGCGGTGACGGGTGAGACC

TATCI_DSM13699{T} CATCAGCAAACCTATCTGCACGGCGTACCAAGTGCACCACTGAATGTGGTAGGCGAAACT

TATMO_LMG23360{T} CATCAGCAAACCTATCTGCACGGTGTGCCAAGTGCGCCGCTGAATGTGGTGGGCGAAACT

TATPT_ATCC33301{T} CATCAGCAAATTTATCAGCACGGTGTACCGACAGCACCGCTGGCGGTGATCGGCGAAACA

TATSA_NML06-3099{T} CATCAGCAGATCTATCAACACGGTGTGCCGACGGCACCGCTGGCAGTGATTGGTGAAACT

** ***** ** ** ** ** ** * ** * ** ** **

**F3** (cont’d)

**GATGCAACCG**

PANST_CCUG26359{T} GACGCGACCGGTACCCGTATCCGTTTCTGGCCAAGCCATGACACGTTCACCAACGTGACC

PANST_LMG2632{PT} GACGCGACCGGTACCCGTATCCGTTTCTGGCCAAGCCATGACACGTTCACCAACGTGACC

PANAL_LMG24248{T} GACGCAACCGGTACCCGCATCCGTTTCTGGCCAAGTCATGACACCTTTACCAACGTTACC

PANAN_LMG2665{T} GATGCAACCGGTACCCGCATTCGTTTCTGGCCAAGCCATGACACCTTCACCAACGTTACC

PANAN_97-1 GATGCAACCGGTACCCGCATTCGTTTCTGGCCAAGCCATGACACCTTCACCAACGTTACC

PANAN_LMG5342 GATGCAACCGGTACCCGCATTCGTTTCTGGCCAAGCCATGACACCTTCACCAACGTTACC

PANAN_LMG20103 GATGCAACCGGTACCCGCATTCGTTTCTGGCCAAGCCATGACACCTTCACCAACGTTACC

PANAN_NN08200 GATGCAACCGGTACCCGCATTCGTTTCTGGCCAAGCCATGACACCTTCACCAACGTTACC

PANAN_ARC311 GATGCAACCGGTACCCGCATTCGTTTCTGGCCAAGCCACGACACCTTCACCAACGTTACC

PANAN_RSA47 GATGCAACCGGTACCCGCATTCGTTTTTGGCCAAGCCACGACACCTTCACCAACGTTACC

PANAN_SGAir0210 GATGCAACCGGTACCCGCATTCGTTTTTGGCCAAGCCACGACACCTTCACCAACGTTACC

MIXCA_DSM22759{T} GATCTGACCGGTACCCGCGTGCGCTTCTGGCCAAGCCATCAGACCTTTACCAACGTCACC

MIXGA_DSM22758{T} GATCTGACCGGCACCCGCGTGCGTTTCTGGCCGAGCCACCAGACCTTTACCAACGTTACC

MIXAL_LTYR-11Z{T} GATTTGACCGGTACGCGCGTGCGTTTCTGGCCAAGCCATCAGACCTTTACCAACGTTGTG

MIXTH_QC88-366{T} GATCTGACCGGGACGCGCGTGCGTTTCTGGCCAAGCCATCGGACGTTTACCAACGTGGTC

PANRO_LMG26273{T} GAAGCCACCGGGACTCGCGTGCGCTTCTGGCCGAGCTACGAAACCTTCACTAACGTGATT

PANRW_LMG26275{T} GAAGCCACCGGTACGCGTGTCCGCTTCTGGCCGAGCTATGAAACCTTCACCAACGTCATC

PANCY_LMG2657{T} GAAGCCACCGGTACGCGCGTGCGTTTCTGGCCGAGCCATGAAACCTTCACCAATGTGACG

PANEU_LMG5346{T} GAGACGACCGGTACGCGCGTACGCTTCTGGCCAAGCCACGAGACCTTTACCAACGTCACC

PANWA_LMG26277{T} GATGCGACCGGTACGCGCGTACGTTTCTGGCCAAGCCATGAAACCTTCACCAACGTAGTC

PANDI_CCUG25232{T} GACGCTACCGGTACGCGCGTGCGTTTCTGGCCGAGCCACGATACGTTCACCAACGTCACC

PANSE_LMG5345{T} GATCTGACCGGCACCCGCGTGCGTTTCTGGCCGAGCCACGAAACCTTCACCAACGTTGTC

PANBR_LMG5343{T} GATTTGACCGGTACCCGTGTGCGCTTCTGGCCAAGCCACGAAACCTTTACCAACGTGCGC

PANCO_LMG24534{T} GATTTGACCGGTACCCGTGTGCGCTTCTGGCCAAGCCACGAAACCTTTACCAACGTGCGC

PANAN_LMG2558{T} GATTTAACCGGTACCCGCGTCCGTTTCTGGCCGAGCTACGAAACCTTCACCAATGTCCGC

PANDE_LMG24200{T} GATTTAACCGGTACCCGCGTGCGTTTCTGGCCAAGCTATGAAACCTTCACCAACGTCCGC

PANVA_LMG24199{T} GACATCACCGGTACCCGCGTGCGTTTCTGGCCAAGCTATGAAACCTTTACCAACGTGCGC

PANEU_LMG24197{T} GACATTACCGGGACCCGCGTGCGTTTCTGGCCGAGCCACGAAACCTTTACCAACGTGCGC

PANAG_DSM3493{T} GACATTACCGGGACCCGTGTGCGTTTCTGGCCAAGCCACGAAACCTTTACCAACGTGCGC

PANAG_CFBP13505 GACATTACCGGGACCCGAGTGCGTTTCTGGCCGAGCCACGAAACCTTTACCAACGTGCGC

TATCI_DSM13699{T} GATTCTACCGGGACTTCAGTTCGTTTCTGGGCCAGCCATCAGACGTTTACCAATGTCACT

TATMO_LMG23360{T} GACTCCACGGGAACATCGGTTCGTTTCTGGGCCAGTCATCAGACGTTTACCAATGTCACC

TATPT_ATCC33301{T} GATGTGAGCGGGACTGCGGTACGTTTCTGGGCCAGTCATCAGACATTTACGAATGTGACC

TATSA_NML06-3099{T} GACGTCAGTGGGACCTCCGTCCGCTTCTGGGCCAGTCACGAGACCTTCACCAATGTGACC

** * ** ** * ** ** *** * ** * ** ** ** ** **

**F2**  **F-Loop**  **F1c**

**TCGAATACGACATTCTGGC GCGAACTGTCCTTCCTGAA TCTGGC**

PANST_CCUG26359{T} GACTTCGAATACGACATTCTGGCGAAGCGCCTGCGTGAGCTCTCTTTCCTGAACTCCGGT

PANST_LMG2632{PT} GACTTCGAATACGACATTCTGGCGAAGCGCCTGCGTGAGCTCTCTTTCCTGAACTCCGGT

PANAL_LMG24248{T} GATTTCGAATACGACATTCTGGCGAAACGCCTGCGTGAACTGTCCTTCCTGAACTCTGGC

PANAN_LMG2665{T} GATTTCGAATATGACATTCTGGCGAAACGCCTGCGCGAACTGTCCTTCCTGAACTCTGGC

PANAN_97-1 GATTTCGAATATGACATTCTGGCGAAACGCCTGCGCGAACTGTCCTTCCTGAACTCTGGC

PANAN_LMG5342 GATTTCGAATACGACATTCTGGCGAAACGCCTGCGCGAACTGTCCTTCCTGAACTCTGGC

PANAN_LMG20103 GATTTCGAATACGACATTCTGGCGAAACGCCTGCGCGAACTGTCCTTCCTGAACTCTGGC

PANAN_NN08200 GATTTCGAATACGACATTCTGGCGAAACGCCTGCGCGAACTGTCCTTCCTGAACTCTGGC

PANAN_ARC311 GATTTCGAATACGACATTCTGGCGAAACGCCTGCGCGAACTGTCCTTCCTGAACTCTGGC

PANAN_RSA47 GATTTCGAATACGACATTCTGGCGAAACGCCTGCGCGAACTGTCCTTCCTGAACTCTGGC

PANAN_SGAir0210 GATTTCGAATACGACATTCTGGCGAAACGCCTGCGCGAACTGTCCTTCCTGAACTCTGGC

MIXCA_DSM22759{T} GATTTCGAATATGAAATTCTGGCGAAGCGTCTGCGCGAGCTGTCGTTCCTGAACTCCGGC

MIXGA_DSM22758{T} GATTTCGAATATGAAATTCTGGCGAAGCGTCTGCGCGAACTGTCGTTCCTGAACTCCGGC

MIXAL_LTYR-11Z{T} GAATTCGAATATGAAATCCTGGCCAAGCGTCTGCGTGAGCTGTCGTTTCTGAACTCCGGC

MIXTH_QC88-366{T} GATTTCGAATATGAAATCCTGGCCAAGCGCCTGCGCGAGCTGTCGTTCCTGAACTCCGGC

PANRO_LMG26273{T} GAGTTTGAGTACGATATCCTGGCAAAACGCCTGCGTGAACTGTCGTTCCTGAACTCTGGC

PANRW_LMG26275{T} GAATTCGAATACGACATCCTGGCAAAACGCCTGCGTGAACTGTCGTTCCTGAACTCGGGC

PANCY_LMG2657{T} GAATTTGAGTACGAGATCCTGGCAAAACGCCTGCGCGAACTGTCGTTCCTCAATTCTGGC

PANEU_LMG5346{T} GATTTTGAATATGAGATTCTTGCTAAACGCCTGCGTGAGCTGTCGTTCCTGAACTCCGGC

PANWA_LMG26277{T} GATTTCGAATACGAAATTCTGGCGAAGCGTCTGCGTGAGCTGTCGTTCCTGAACTCCGGC

PANDI_CCUG25232{T} GATTTTGAATATGAAATCCTGGCGAAGCGTCTGCGCGAACTGTCATTCCTTAACTCCGGC

PANSE_LMG5345{T} GAGTTCGAATACGACATCCTGGCGAAGCGCCTGCGCGAACTCTCGTTCCTGAACTCCGGC

PANBR_LMG5343{T} GATTTTGAGTATGACATCCTGGCGAAACGCCTGCGTGAACTGTCGTTCCTCAACTCCGGC

PANCO_LMG24534{T} GATTTTGAGTATGACATTCTGGCGAAACGCCTGCGTGAACTCTCATTCCTCAACTCAGGC

PANAN_LMG2558{T} GATTTTGAGTATGACATTCTGGCGAAACGCCTGCGTGAACTCTCCTTCCTGAACTCAGGC

PANDE_LMG24200{T} GACTTCGAGTATGACATTCTGGCAAAACGCCTGCGTGAACTCTCGTTCCTGAACTCGGGC

PANVA_LMG24199{T} GATTTCGAGTATGAGATTCTGGCAAAACGCCTGCGCGAACTGTCGTTCCTGAACTCGGGC

PANEU_LMG24197{T} GATTTTGAGTATGACATTCTGGCGAAACGCCTGCGTGAACTCTCATTCCTCAACTCGGGC

PANAG_DSM3493{T} GATTTTGAGTATGACATCCTGGCGAAACGCCTGCGTGAACTCTCATTCCTGAACTCCGGC

PANAG_CFBP13505 GATTTTGAGTATGACATCCTGGCGAAACGCCTGCGTGAACTCTCATTCCTGAACTCCGGC

TATCI_DSM13699{T} GACTTTGAGTATGACATCCTGGCGAAGCGCCTGCGTGAACTGTCGTTCCTGAATTCAGGG

TATMO_LMG23360{T} GATTTTGAATATGACATCCTGGCGAAACGGCTGCGCGAACTGTCGTTCCTGAATTCCGGA

TATPT_ATCC33301{T} GATTTCGAATACGACATTCTGGCAAAACGCCTGCGCGAACTCTCTTTCCTGAATTCCGGC

TATSA_NML06-3099{T} GATTTCGAATACGATATTCTGGCTAAACGCCTGCGTGAGCTGTCATTCCTCAACTCTGGG

** ** ** ** ** ** ** ** ** ** ***** ** ** ** ** ** ** ** **

**F1c** (cont’d) **B1c** **B-Loop**

**GTATCGATTCGTCTG GCGTGATGCAAGAAACGACC CACTACGAAGGT**

PANST_CCUG26359{T} GTTTCCATTCGTCTGGAAGACAAACGTGACGGCAAAAACGACCATTTCCACTATGAAGGT

PANST_LMG2632{PT} GTTTCCATTCGTCTGGAAGACAAACGTGACGGCAAAAACGACCATTTCCACTATGAAGGT

PANAL_LMG24248{T} GTGTCGATCCGTCTGGAAGACAAGCGTGATGCAAGAAATGACCACTTCCACTATGAAGGC

PANAN_LMG2665{T} GTATCGATTCGTCTGGAAGACAAGCGTGATGCAAGAAACGACCACTTTCACTACGAAGGT

PANAN_97-1 GTATCGATTCGTCTGGAAGACAAGCGTGATGCAAGAAACGACCACTTTCACTACGAAGGT

PANAN_LMG5342 GTATCGATTCGTCTGGAAGACAAGCGTGATGCAAGAAACGACCACTTTCACTACGAAGGT

PANAN_LMG20103 GTATCGATTCGTCTGGAAGACAAGCGTGATGCAAGAAACGACCACTTTCACTACGAAGGT

PANAN_NN08200 GTATCGATTCGTCTGGAAGACAAGCGTGATGCAAGAAACGACCACTTTCACTACGAAGGT

PANAN_ARC311 GTATCGATTCGTCTGGAAGACAAGCGTGATGCAAGAAACGACCACTTTCACTACGAAGGT

PANAN_RSA47 GTATCGATTCGTCTGGAAGACAAGCGTGATGCAAGAAACGACCACTTTCACTACGAAGGT

PANAN_SGAir0210 GTATCTATTCGTCTGGAAGACAAGCGTGATGCAAGAAACGACCACTTTCACTACGAAGGT

MIXCA_DSM22759{T} GTCTCTATCCGCCTGGAAGACAAACGTACTGACAAGAGCGACCACTATCACTACGAAGGC

MIXGA_DSM22758{T} GTCTCTATCCGCCTGGAAGACAAGCGCACCGACAAGAGCGACCATTATCACTACGAAGGC

MIXAL_LTYR-11Z{T} GTGTCTATCCGTCTGGAAGACAAACGCACGGATAAAAGCGATCACTACCATTACGAAGGC

MIXTH_QC88-366{T} GTTTCTATCCGCCTGGAAGACAAACGCACGGATAAAAGCGATCACTATCATTACGAAGGC

PANRO_LMG26273{T} GTCTCCATCCGTCTGGAAGACAAGCGTGATGGCAAGGCTGACCACTTCCATTATGAAGGT

PANRW_LMG26275{T} GTGTCGATTCGTCTGGAAGATAAACGTGATGGCAGGAGCGACCATTATCACTACGAAGGG

PANCY_LMG2657{T} GTGTCGATTCGCCTTGAAGACAAGCGCGATGACAAAA-CGACCATTACCACTACGAAGGT

PANEU_LMG5346{T} GTCTCTATTCGCCTGCAGGATAAGCGTGATGACAAGAGCGATCACTACCACTATGAAGGC

PANWA_LMG26277{T} GTATCGATTCGTCTGGAAGACAAACGTGACGACAAGAACGATCACTACCACTACGAAGGC

PANDI_CCUG25232{T} GTGTCGATTCGCCTGGAAGACAAGCGCGACGATAAAAACGACCATTACCACTACGAAGGT

PANSE_LMG5345{T} GTATCGATTCGCCTGGAAGACAAGCGTGACGGCAAAAGCGATCACTTCCATTACGAAGGC

PANBR_LMG5343{T} GTGTCGATTCGTCTGGAAGATAAGCGTGACGGCAAGTCTGATCACTTCCATTACGAAGGC

PANCO_LMG24534{T} GTGTCGATTCGTCTGGAAGATAAGCGTGACGGCAAGTCTGACCATTTCCATTATGAAGGC

PANAN_LMG2558{T} GTTTCGATCCGTCTGGAAGATAAGCGCGACGGCAAAACGGATCACTTCCACTACGAAGGT

PANDE_LMG24200{T} GTGTCGATCCGTCTGGAAGACAAGCGTGATGGCAAAAGCGATCACTTCCATTACGAAGGC

PANVA_LMG24199{T} GTGTCGATTCGCCTGGAAGACAAGCGTGATGGCAAAACCGATCACTTCCATTACGAAGGC

PANEU_LMG24197{T} GTGTCTATTCGTCTGGAAGATAAGCGCGATGGCAAAAACGATCACTTCCATTACGAAGGC

PANAG_DSM3493{T} GTGTCGATTCGTCTGGATGATAAGCGTGATGGTAAAAGTGATCACTTCCACTATGAAGGC

PANAG_CFBP13505 GTGTCGATTCGTCTGGATGATAAGCGTGATGGCAAAAGCGATCACTTCCACTATGAAGGC

TATCI_DSM13699{T} GTCTCTATCCGGCTGATCGACAAGCGTTCAGACAAGAGTGATCATTATCACTATGAAGGC

TATMO_LMG23360{T} GTCTCTATCCGGTTGATCGACAAACGTTCCGACAAGAGCGACCATTATCATTATGAAGGC

TATPT_ATCC33301{T} GTGTCTATCCGCCTGATCGACAAGCGTACAGATAAGAGTGATCACTACCACTACGAAGGT

TATSA_NML06-3099{T} GTCTCTATCCGCCTGATTGATAAGCGAGTGGATAAGAGCGATCACTACCACTATGAAGGT

** ** ** ** * ** ** ** * * ** ** * ** ** *****

**B-Loop** (cont’d) **B2**

**GGTATCCG GCCTTTGTTGAGTACCTGAA**

PANST_CCUG26359{T} GGGATTAAAGCGTTTGTCGAATACCTGAACAAGAACAAAACGCCTATCCATCCGACCGTA

PANST_LMG2632{PT} GGGATTAAAGCGTTTGTTGAGTACCTGAACAAGAACAAAACGCCTATCCATCCGACCGTA

PANAL_LMG24248{T} GGCATTCGTGCTTTTGTTGAGTATCTGAATAAGAACAAAACGCCTATTCACCCCACCGTA

PANAN_LMG2665{T} GGTATCCGCGCCTTTGTTGAGTACCTGAATAAAAACAAAACGCCTATTCACCCAACCGTA

PANAN_97-1 GGTATCCGCGCCTTTGTTGAGTACCTGAATAAAAACAAAACGCCTATTCACCCAACCGTA

PANAN_LMG5342 GGTATCCGCGCCTTTGTTGAGTACCTGAATAAAAACAAAACGCCTATTCACCCAACCGTA

PANAN_LMG20103 GGTATCCGCGCCTTTGTTGAGTACCTGAATAAAAACAAAACGCCTATTCACCCAACCGTA

PANAN_NN08200 GGTATCCGCGCCTTTGTTGAGTACCTGAATAAAAACAAAACGCCTATTCACCCAACCGTA

PANAN_ARC311 GGTATCCGCGCCTTTGTTGAGTACCTGAATAAAAACAAAACGCCTATTCACCCAACCGTA

PANAN_RSA47 GGTATCCGCGCCTTTGTTGAGTACCTGAATAAAAACAAAACGCCTATTCACCCAACCGTA

PANAN_SGAir0210 GGTATCCGCGCCTTTGTTGAGTACCTGAATAAAAACAAAACGCCTATTCACCCAACCGTA

MIXCA_DSM22759{T} GGCATCAAAGCTTTCGTTGAATACCTGAACAAAAACAAAAACCCGATCCATCCGAACGTC

MIXGA_DSM22758{T} GGTATCAAAGCCTTTGTTGAGTACCTGAACAAAAACAAAAACCCAATCCATCCGAACGTC

MIXAL_LTYR-11Z{T} GGCATCAAGGCCTTTGTTGAATACCTGAACAAAAACAAAACGCCTATCCATCCCAACGTG

MIXTH_QC88-366{T} GGCATCAAGGCGTTCGTTGAATACCTGAACAAAAATAAAACCCCGATCCATCCGAACGTT

PANRO_LMG26273{T} GGCATCAAGGCGTTTGTTGAGTACCTCAACAAAAACAAAACCCCCATTCACCCAACCGTA

PANRW_LMG26275{T} GGGATTCGTGCGTTTGTTGAGTATCTCAACAAGAACAAAACCCCGATTCACCCAACCGTG

PANCY_LMG2657{T} GGTATCAAGGCGTTTGTTGAATACCTCAACCGCAACAAAAATCCGATTCACCCCAATGTG

PANEU_LMG5346{T} GGCATCAGGGCGTTTGTGGAGTATCTGAACCGCAACAAAAACCCGATTCACCCTAACGTA

PANWA_LMG26277{T} GGTATCAAAGCCTTTGTTGAGTACCTGAACCGTAACAAAAACCCGATTCACCCGAACGTG

PANDI_CCUG25232{T} GGTATCAAAGCGTTTGTTGAATATCTCAACCGCAACAAGAACCCGATTCACCCGAATGTG

PANSE_LMG5345{T} GGCATCAAGGCGTTTGTCGAGTATCTGAATAAAAATAAAACCCCTATCCATCCGAATGTG

PANBR_LMG5343{T} GGCATCCGGGCATTCGTTGAATACCTCAACAAAAACAAAACCCCGATTCACCCAACCGTG

PANCO_LMG24534{T} GGCATCCGGGCGTTTGTTGAGTACCTCAACAAAAACAAAACCCCGATTCACCCAACCGTG

PANAN_LMG2558{T} GGTATCAAGGCGTTTGTTGAGTACCTGAACAAAAACAAAACCCCGATTCATCCGACCGTT

PANDE_LMG24200{T} GGCATCAAGGCGTTTGTTGAGTACCTCAACAAGAACAAAACCCCAATCCACCCGACCGTG

PANVA_LMG24199{T} GGCATCAAGGCGTTTGTTGAGTACCTCAACAAAAACAAAACTCCGATTCACCCTACCGTG

PANEU_LMG24197{T} GGCATCCGGGCGTTTGTTGAGTACCTGAACAAAAACAAAACCCCTATTCACCCTACCGTG

PANAG_DSM3493{T} GGCATCCGGGCGTTTGTTGAGTACCTCAACAAAAACAAAACCCCGATTCATCCTACCGTG

PANAG_CFBP13505 GGTATCCGGGCGTTTGTTGAGTACCTCAACAAAAACAAAACCCCGATTCACCCTACCGTA

TATCI_DSM13699{T} GGCATTAAAGCGTTCGTTGAATATCTGAATAAAAATAAAACTCCAATTCATCCGAACGTA

TATMO_LMG23360{T} GGAATTAAAGCGTTCGTTGAATATCTGAACAAAAATAAAACGCCAATTCACCCGAACGTG

TATPT_ATCC33301{T} GGTATCCGCGCGTTTGTGGAGTATCTGAATAAAAATAAAACGCCTATCCATCCGAACGTC

TATSA_NML06-3099{T} GGTATCCGCGCATTTGTTGAATATCTAAATAAAAATAAAACCCCGATCCATCCCAATGTC

** ** ** ** ** ** ** ** ** ** ** * ** ** ** ** * **

**B3**

**CGAGAAAGACGGCATTGG**

PANST_CCUG26359{T} TTCTATTTCTCAACCGAAAAAGACGGCATTGGCGTAGAAGTAGCCCTGCAGTGGAACGAC

PANST_LMG2632{PT} TTCTATTTCTCAACCGAAAAAGACGGCATTGGCGTGGAAGTAGCCCTGCAGTGGAACGAC

PANAL_LMG24248{T} TTCTATTTCTCGACCGAGAAAGATGGCATTGGCGTAGAAGTGGCACTGCAGTGGAATGAC

PANAN_LMG2665{T} TTCTATTTCTCAACCGAGAAAGACGGCATTGGCGTGGAAGTCGCACTGCAGTGGAATGAC

PANAN_97-1 TTCTATTTCTCAACCGAGAAAGACGGCATTGGCGTGGAAGTCGCACTGCAGTGGAATGAC

PANAN_LMG5342 TTCTATTTCTCAACCGAGAAAGACGGCATTGGCGTGGAAGTCGCACTGCAGTGGAATGAC

PANAN_LMG20103 TTCTATTTCTCAACCGAGAAAGACGGCATTGGCGTGGAAGTCGCACTGCAGTGGAATGAC

PANAN_NN08200 TTCTATTTCTCAACCGAGAAAGACGGCATTGGCGTGGAAGTCGCACTGCAGTGGAATGAC

PANAN_ARC311 TTCTATTTCTCAACCGAGAAAGACGGCATTGGCGTGGAAGTCGCACTGCAGTGGAATGAC

PANAN_RSA47 TTCTATTTCTCAACCGAGAAAGACGGCATTGGCGTGGAAGTCGCACTGCAGTGGAATGAC

PANAN_SGAir0210 TTCTATTTCTCAACCGAGAAAGACGGCATTGGCGTGGAAGTCGCACTGCAGTGGAATGAC

MIXCA_DSM22759{T} TTCTACTTCTCTACTGAGAAAGACGGCATCGGCGTGGAAGTGGCATTGCAGTGGAACGAC

MIXGA_DSM22758{T} TTCTATTTCTCGACCGAGAAAGATGGCATCGGTGTGGAAGTGGCGCTGCAGTGGAACGAC

MIXAL_LTYR-11Z{T} TTCTACTTCTCTACCGAGAAGGATGGCATCGGCGTGGAAGTGGCGCTGCAGTGGAACGAC

MIXTH_QC88-366{T} TTCTACTTCTCTACCGAGAAGGATGGCATCGGCGTGGAAGTGGCGCTGCAGTGGAACGAT

PANRO_LMG26273{T} TTCTATTTCTCTACTGAGAAAGACGGCATCGGCGTGGAAGTGGCGCTGCAGTGGAACGAT

PANRW_LMG26275{T} TTCTATTTCTCTACCGAGAAAGACGGTATCGGCGTAGAAGTGGCGCTGCAGTGGAACGAT

PANCY_LMG2657{T} TTCTATTTCTCCACTGAGAAGGACGGCATTGGTGTGGAAGTGGCGTTGCAGTGGAATGAT

PANEU_LMG5346{T} TTCTATTTCTCTACCGAGAAAGATGGCATCGGTGTGGAAGTGGCGCTGCAGTGGAACGAT

PANWA_LMG26277{T} TTCTATTTCTCAACCGAGAAAGATGGCATTGGCGTAGAAGTGGCGCTGCAGTGGAATGAT

PANDI_CCUG25232{T} TTCTACTTCTCCACCGAAAAAGACGGTATCGGCGTTGAAGTTGCGCTGCAGTGGAACGAC

PANSE_LMG5345{T} TTCTATTTCTCTACCGAGAAAGACGGCATTGGCGTGGAAGTGGCGCTGCAGTGGAATGAC

PANBR_LMG5343{T} TTCTATTTCTCTACCGAGAAAGATGGCATCGGTGTGGAAGTGGCGCTGCAGTGGAACGAT

PANCO_LMG24534{T} TTCTATTTCTCTACCGAGAAAGATGGCATCGGTGTGGAAGTGGCGCTGCAGTGGAACGAC

PANAN_LMG2558{T} TTCTACTTCTCAAACGAGAAAGATGGCATCGGCGTGGAAGTGGCGCTGCAGTGGAACGAC

PANDE_LMG24200{T} TTCTATTTCTCTAACGAGAAAGATGGCATCGGCGTAGAAGTGGCGCTGCAGTGGAACGAC

PANVA_LMG24199{T} TTCTATTTCTCTACCGAGAAAGATGGCATTGGCGTGGAAGTGGCGCTGCAGTGGAACGAC

PANEU_LMG24197{T} TTCTATTTCTCTACTGAGAAAGATGGCATCGGTGTGGAAGTGGCACTGCAGTGGAACGAC

PANAG_DSM3493{T} TTCTATTTCTCTACCGAGAAAGATGGCATTGGTGTGGAAGTGGCGCTGCAGTGGAACGAC

PANAG_CFBP13505 TTCTATTTCTCTACCGAGAAAGATGGCATTGGTGTGGAAGTGGCGCTGCAGTGGAACGAC

TATCI_DSM13699{T} TTCTATTTCAGTACCGAAAAAGACGGTATTGGTGTGGAAGTCGCTTTGCAGTGGAACGAC

TATMO_LMG23360{T} TTCTACTTCAGTACCGAAAAAGACGGTATTGGTGTGGAGGTTGCTCTGCAATGGAACGAT

TATPT_ATCC33301{T} TTCTATTTCAGTACGGAAAAAGACGGTATCGGCGTGGAAGTTGCGTTGCAGTGGAACGAT

TATSA_NML06-3099{T} TTCTATTTCAGTACCGAAAAAGATGGGATTGGCGTTGAAGTTGCGCTGCAATGGAACGAC

***** *** * ** ** ** ** ** ** ** ** ** ** **** ***** **

PANST_CCUG26359{T} GGCTTCCAGGAGAACATCTACTGCTTCACTAACAACATTCCTCAACGCGATGGCGGTACG

PANST_LMG2632{PT} GGCTTCCAGGAGAACATCTACTGCTTCACTAACAACATTCCTCAACGCGACGGCGGTACG

PANAL_LMG24248{T} GGCTTCCAGGAAAACATTTACTGCTTTACCAACAATATTCCACAGCGCGACGGCGGTACG

PANAN_LMG2665{T} GGCTTCCAGGAGAACATCTACTGCTTCACGAACAATATTCCACAGCGCGACGGCGGTACA

PANAN_97-1 GGCTTCCAGGAGAACATCTACTGCTTCACGAACAATATTCCACAGCGCGACGGCGGTACA

PANAN_LMG5342 GGCTTCCAGGAGAACATCTACTGCTTCACGAACAATATTCCACAGCGCGACGGCGGTACA

PANAN_LMG20103 GGCTTCCAGGAGAACATCTACTGCTTCACGAACAATATTCCACAGCGCGACGGCGGTACA

PANAN_NN08200 GGCTTCCAGGAGAACATCTACTGCTTCACGAACAATATTCCACAGCGCGACGGCGGTACA

PANAN_ARC311 GGCTTCCAGGAGAACATCTACTGCTTCACCAACAATATTCCACAGCGCGACGGCGGTACA

PANAN_RSA47 GGCTTCCAGGAGAACATCTACTGCTTCACCAACAATATTCCACAGCGCGACGGCGGTACA

PANAN_SGAir0210 GGCTTCCAGGAGAACATCTACTGCTTCACCAACAATATTCCACAGCGCGACGGCGGTACA

MIXCA_DSM22759{T} GGTTTCCAGGAAAACATCTACTGCTTCACCAACAACATTCCGCAGCGCGACGGCGGCACG

MIXGA_DSM22758{T} GGTTTCCAGGAAAACATCTACTGCTTCACCAACAACATTCCGCAGCGCGACGGCGGTACG

MIXAL_LTYR-11Z{T} GGTTTCCAGGAAAACATCTACTGCTTCACCAACAACATTCCGCAGCGCGACGGCGGTACG

MIXTH_QC88-366{T} GGTTTCCAGGAAAACATTTACTGTTTCACTAACAATATTCCGCAGCGCGACGGCGGTACG

PANRO_LMG26273{T} GGTTTCCAGGAAAACATCTACTGCTTCACCAACAACATTCCACAGCGCGATGGTGGTACC

PANRW_LMG26275{T} GGTTTCCAGGAAAACATTTACTGCTTCACCAACAATATTCCACAGCGCGATGGCGGTACG

PANCY_LMG2657{T} GGTTTCCAGGAAAACATCTACTGCTTCACCAACAACATTCCGCAGCGCGATGGCGGTACT

PANEU_LMG5346{T} GGTTTCCAGGAAAACATCTACTGCTTTACCAATAACATTCCGCAGCGCGACGGCGGGACG

PANWA_LMG26277{T} GGCTTCCAGGAGAACATCTACTGCTTTACCAACAACATCCCACAGCGTGATGGCGGTACG

PANDI_CCUG25232{T} GGTTTCCAGGAAAACATCTACTGCTTCACCAACAATATTCCGCAGCGCGACGGCGGAACG

PANSE_LMG5345{T} GGCTTCCAGGAGAACATCTACTGCTTCACCAACAACATTCCACAGCGCGACGGCGGTACG

PANBR_LMG5343{T} GGCTTCCAGGAAAACATCTACTGCTTCACCAACAACATCCCACAGCGTGACGGCGGTACC

PANCO_LMG24534{T} GGCTTCCAGGAAAACATCTACTGCTTTACCAACAACATCCCACAGCGTGATGGCGGTACC

PANAN_LMG2558{T} GGTTTCCAGGAAAATATCTACTGCTTTACCAACAACATCCCGCAGCGTGACGGCGGTACG

PANDE_LMG24200{T} GGCTTCCAGGAAAACATCTACTGCTTCACCAACAACATTCCACAGCGCGACGGCGGTACG

PANVA_LMG24199{T} GGCTTCCAGGAAAATATTTACTGCTTTACCAACAACATCCCGCAGCGCGATGGCGGTACG

PANEU_LMG24197{T} GGTTTCCAGGAAAACATCTACTGCTTTACCAACAATATCCCGCAGCGTGACGGTGGTACA

PANAG_DSM3493{T} GGCTTCCAGGAAAATATCTACTGCTTTACCAACAACATCCCACAGCGCGACGGCGGTACA

PANAG_CFBP13505 GGCTTCCAGGAAAATATCTACTGCTTTACCAACAACATCCCACAGCGCGATGGCGGTACA

TATCI_DSM13699{T} GGTTTCCAGGAAAATATTTACTGCTTTACCAACAATATCCCTCAGCGTGATGGCGGAACA

TATMO_LMG23360{T} GGTTTCCAGGAAAATATTTACTGCTTTACTAACAACATCCCTCAGCGTGATGGTGGAACA

TATPT_ATCC33301{T} AGCTTCCAGGAAAATATCTACTGCTTTACCAACAACATTCCGCAGAGGGATGGCGGGACG

TATSA_NML06-3099{T} AGTTTCCAGGAAAACATCTACTGCTTTACCAACAATATTCCGCAGCGTGACGGTGGTACC

* ******** ** ** ***** ** ** ** ** ** ** ** * ** ** ** **

PANST_CCUG26359{T} CACCTTGCCGGTTTCCGCGCCGCGATGACGCGTACCCTGAATGCCTACATGGATAAAGAG

PANST_LMG2632{PT} CACCTTGCCGGTTTCCGCGCCGCGATGACGCGTACCCTGAATGCCTACATGGATAAAGAG

PANAL_LMG24248{T} CACCTTGCCGGTTTCCGCGCGGCGATGACGCGCACCCTGAATGCCTATATGGATAAAGAG

PANAN_LMG2665{T} CACCTTGCGGGTTTCCGCGCCGCGATGACGCGTACGCTGAATGCCTACATGGATAAAGAA

PANAN_97-1 CACCTTGCGGGTTTCCGCGCCGCGATGACGCGTACGCTGAATGCCTACATGGATAAAGAA

PANAN_LMG5342 CACCTTGCGGGTTTCCGCGCCGCGATGACGCGTACGCTGAATGCCTACATGGATAAAGAA

PANAN_LMG20103 CACCTTGCGGGTTTCCGCGCCGCGATGACGCGTACGCTGAATGCCTACATGGATAAAGAA

PANAN_NN08200 CACCTTGCGGGTTTCCGCGCCGCGATGACGCGTACGCTGAATGCCTACATGGATAAAGAA

PANAN_ARC311 CACCTTGCGGGTTTCCGCGCCGCGATGACGCGTACGCTAAATGCCTACATGGATAAAGAA

PANAN_RSA47 CACCTTGCGGGTTTCCGCGCCGCGATGACGCGTACGCTGAATGCCTACATGGATAAAGAA

PANAN_SGAir0210 CATCTTGCGGGTTTCCGCGCCGCGATGACGCGTACGCTGAATGCCTACATGGATAAAGAA

MIXCA_DSM22759{T} CACCTGGCTGGCTTCCGCGCCGCCATGACGCGTACCCTGAACGCCTATATGGATAAAGAA

MIXGA_DSM22758{T} CACCTGGCGGGCTTCCGCGCCGCCATGACGCGTACCCTGAACGCCTATATGGATAAAGAA

MIXAL_LTYR-11Z{T} CACCTGGCGGGCTTCCGTGCGGCGATGACGCGTACGCTGAATGCCTATATGGATAAAGAA

MIXTH_QC88-366{T} CACCTGGCGGGCTTCCGCGCGGCGATGACGCGTACGCTGAACGCCTATATGGATAAAGAA

PANRO_LMG26273{T} CATCTGGCCGGTTTCCGTGCGGCAATGACGCGTACGATGAATGCCTACATGGATAAAGAA

PANRW_LMG26275{T} CACCTGGCCGGTTTCCGTGCGGCAATGACGCGTACGCTGAATGCTTACATGGATAAAGAA

PANCY_LMG2657{T} CACCTTGCGGGCTTCCGTGCGGCGATGACGCGTACCCTGAATGCCTATATGGACAAAGAA

PANEU_LMG5346{T} CATCTCGCCGGTTTCCGTGCGGCGATGACGCGCACGCTCAATGCCTATATGGATAAAGAG

PANWA_LMG26277{T} CACCTTGCCGGTTTCCGTGCGGCGATGACGCGTACGCTCAACGCGTACATGGATAAAGAG

PANDI_CCUG25232{T} CACCTTGCCGGTTTCCGCGCGGCGATGACGCGTACGCTCAACGCCTACATGGATAAAGAA

PANSE_LMG5345{T} CACCTCGCGGGCTTCCGCGCGGCGATGACGCGTACCCTGAACGCCTATATGGACAAAGAA

PANBR_LMG5343{T} CACCTTGCGGGCTTCCGTGCGGCGATGACCCGTACCCTGAATGCCTACATGGATAAAGAG

PANCO_LMG24534{T} CACCTTGCTGGTTTCCGTGCTGCGATGACCCGAACCCTGAATGCCTACATGGATAAAGAG

PANAN_LMG2558{T} CACCTTGCCGGTTTCCGTGCGGCGATGACCCGTACCCTGAACGCCTACATGGATAAAGAG

PANDE_LMG24200{T} CACCTGGCCGGTTTCCGTGCGGCAATGACCCGTACCCTGAATGCCTACATGGATAAAGAA

PANVA_LMG24199{T} CACCTTGCCGGTTTCCGTGCGGCGATGACCCGTACCCTGAATGCCTATATGGATAAAGAG

PANEU_LMG24197{T} CACCTCGCCGGTTTCCGTGCGGCGATGACCCGTACCCTGAATGCCTACATGGACAAAGAG

PANAG_DSM3493{T} CACCTCGCCGGTTTCCGTTCAGCGATGACCCGTACCCTGAATGCCTATATGGAAAAAGAG

PANAG_CFBP13505 CACCTCGCCGGTTTCCGTTCAGCGATGACCCGTACCCTCAATGCCTATATGGAAAAAGAG

TATCI_DSM13699{T} CACCTGGCCGGTTTCCGTGCCTCAATGACCCGTACTCTGAACGCCTACATGGATAAAGAA

TATMO_LMG23360{T} CACCTGGCCGGTTTTCGTGCTTCAATGACCCGTACGCTGAATGCCTATATGGATAAAGAG

TATPT_ATCC33301{T} CACCTTGCCGGTTTCCGTGCCTCAATGACCCGTACACTGAATGCGTATATGGATAAAGAA

TATSA_NML06-3099{T} CATCTGGCCGGTTTCCGAGCCTCGATGACCCGTACCCTGAACGCCTATATGGATAAAGAA

** ** ** ** ** ** * * ***** ** ** * ** ** ** ***** *****

PANST_CCUG26359{T} GGATACAGCAAGAAGGCCAAAGTCAGCGCCACTGGTGATGACGCGCGTGAGGGCCTGATT

PANST_LMG2632{PT} GGATACAGCAAGAAGGCCAAAGTCAGCGCCACCGGTGATGACGCGCGTGAGGGCCTGATT

PANAL_LMG24248{T} GGTTACAGCAAGAAAGCCAAAGTCAGCGCAACCGGTGACGATGCGCGTGAAGGGCTGATT

PANAN_LMG2665{T} GGCTACAGCAAAAAAGCCAAAGTCAGCGCGACCGGTGACGATGCCCGTGAAGGTCTGATT

PANAN_97-1 GGCTATAGCAAAAAAGCCAAAGTCAGCGCGACCGGTGACGATGCCCGTGAAGGTCTGATT

PANAN_LMG5342 GGCTACAGCAAAAAAGCCAAAGTCAGCGCGACCGGTGACGATGCCCGTGAAGGTCTGATT

PANAN_LMG20103 GGCTACAGCAAAAAAGCCAAAGTCAGCGCGACCGGTGACGATGCCCGTGAAGGTCTGATT

PANAN_NN08200 GGCTACAGCAAAAAAGCCAAAGTCAGCGCGACCGGTGACGATGCCCGTGAAGGTCTGATT

PANAN_ARC311 GGCTACAGCAAAAAAGCCAAAGTCAGCGCGACCGGTGACGATGCCCGTGAAGGTCTGATT

PANAN_RSA47 GGCTACAGCAAAAAAGCCAAAGTCAGCGCGACCGGTGACGATGCCCGTGAAGGTCTGATT

PANAN_SGAir0210 GGCTACAGCAAAAAAGCCAAAGTCAGCGCGACCGGTGACGATGCCCGTGAAGGTCTGATT

MIXCA_DSM22759{T} GGCTACAGCAAAAAAGCGAAGGTCAGCGCTACCGGCGACGACGCGCGCGAAGGGCTGATC

MIXGA_DSM22758{T} GGCTACAGCAAAAAAGCGAAGGTCAGCGCCACCGGCGACGACGCGCGCGAAGGCCTGATC

MIXAL_LTYR-11Z{T} GGCTACAGCAAGAAAGCGAAAGTCAGCGCCACCGGCGACGATGCGCGTGAAGGCCTGATT

MIXTH_QC88-366{T} GGCTACAGCAAAAAAGCGAAAGTCAGCGCCACCGGCGACGATGCGCGTGAAGGGCTAATC

PANRO_LMG26273{T} GGCTACAGCAAAAAAGCCAAAATCAGCGCAACGGGTGATGATGCGCGTGAAGGCCTGATT

PANRW_LMG26275{T} GGCTACAGCAAAAAAGCCAAAGTTAGCGCCACCGGTGATGATGCGCGTGAAGGCCTGATT

PANCY_LMG2657{T} GGTTACAGCAAAAAAGCCAAAGTCAGCGCCACCGGCGATGACGCGCGTGAAGGTCTGATT

PANEU_LMG5346{T} GGCTACAGCAAAAAAGCCAAAGTCAGCGCCACCGGTGACGATGCGCGTGAAGGCCTGATT

PANWA_LMG26277{T} GGCTACAGCAAAAAAGCCAAAGTCAGTGCGACCGGTGACGATGCACGTGAAGGTCTGGTC

PANDI_CCUG25232{T} GGTTACAGCAAAAAAGCCAAAGTCAGCGCTACCGGTGACGACGCGCGTGAAGGGCTGGTG

PANSE_LMG5345{T} GGCTACAGCAAGAAAGCGAAAGTCAGCGCCACCGGCGACGACGCCCGCGAAGGCCTGGTC

PANBR_LMG5343{T} GGTTACAGCAAGAAAGCCAAAGTCAGCGCCACCGGTGACGATGCGCGTGAAGGTCTGATC

PANCO_LMG24534{T} GGTTACAGCAAGAAAGCCAAAGTCAGCGCCACCGGTGATGATGCGCGTGAAGGCCTGATC

PANAN_LMG2558{T} GGTTACAGCAAGAAAGCCAAAGTCAGCGCCACCGGTGACGATGCCCGTGAAGGCCTGATT

PANDE_LMG24200{T} GGGTACAGCAAGAAAGCCAAAGTCAGCGCCACCGGCGACGACGCCCGTGAAGGCCTGATC

PANVA_LMG24199{T} GGTTACAGCAAGAAAGCCAAAGTCAGCGCCACCGGTGATGATGCGCGTGAAGGCCTGATC

PANEU_LMG24197{T} GGTTACAGCAAGAAAGCCAAAGTCAGCGCCACCGGTGATGATGCCCGTGAAGGTTTGATC

PANAG_DSM3493{T} GGTTACAGCAAGAAGGCCAAAGTCAGCGCCACCGGTGATGATGCCCGTGAAGGTCTGATC

PANAG_CFBP13505 GGTTACAGCAAGAAGGCTAAAGTCAGCGCCACCGGTGATGATGCCCGTGAAGGTCTGATC

TATCI_DSM13699{T} GGGTACAGCAAAAAAGCCAAAGTCAGTGCAACCGGTGATGATGCCCGTGAAGGACTGATT

TATMO_LMG23360{T} GGTTACAGCAAAAAAGCTAAAGTCAGTGCGACCGGTGATGATGCCCGTGAAGGACTGATT

TATPT_ATCC33301{T} GGCTACAGTAAAAAGGCGAAGGTCAGTGCCACCGGTGATGATGCCCGTGAAGGTCTGATC

TATSA_NML06-3099{T} GGCTACAGCAAGAAAGCCAAAGTCAGCGCTACCGGTGATGACGCCCGTGAAGGTCTGATT

** ** ** ** ** ** ** * ** ** ** ** ** ** ** ** ** ** * *

PANST_CCUG26359{T} GCCGTGGTGTCGGTGAAAGTGCCGGATCCAAAATTCTCTTCACAGACCAAAGACAAGCTG

PANST_LMG2632{PT} GCCGTGGTGTCGGTGAAAGTGCCGGATCCAAAATTCTCTTCACAGACCAAAGACAAGCTG

PANAL_LMG24248{T} GCAGTGGTTTCGGTAAAAGTACCGGATCCGAAATTCTCTTCACAGACCAAAGACAAACTG

PANAN_LMG2665{T} GCGGTCGTGTCGGTCAAAGTGCCGGACCCGAAATTCTCTTCACAGACCAAAGACAAACTG

PANAN_97-1 GCGGTCGTGTCGGTCAAAGTGCCGGACCCGAAATTCTCTTCACAGACCAAAGACAAACTG

PANAN_LMG5342 GCGGTCGTGTCGGTCAAAGTGCCGGACCCGAAATTCTCTTCACAGACCAAAGACAAACTG

PANAN_LMG20103 GCGGTCGTGTCGGTCAAAGTGCCGGACCCGAAATTCTCTTCACAGACCAAAGACAAACTG

PANAN_NN08200 GCGGTCGTGTCGGTCAAAGTGCCGGACCCGAAATTCTCTTCACAGACCAAAGACAAACTG

PANAN_ARC311 GCGGTCGTGTCGGTCAAAGTGCCGGACCCGAAATTCTCTTCACAGACCAAAGACAAACTG

PANAN_RSA47 GCGGTCGTGTCGGTCAAAGTGCCGGACCCGAAATTCTCTTCACAGACCAAAGACAAACTG

PANAN_SGAir0210 GCGGTCGTGTCGGTCAAAGTGCCGGACCCGAAATTCTCTTCACAGACCAAAGACAAACTG

MIXCA_DSM22759{T} GCTGTCGTTTCCGTGAAGGTGCCGGACCCGAAATTCTCCTCGCAGACCAAAGACAAACTG

MIXGA_DSM22758{T} GCCGTGGTATCGGTGAAGGTGCCGGATCCGAAATTCTCCTCGCAGACCAAAGATAAGCTG

MIXAL_LTYR-11Z{T} GCGGTAGTCTCCGTGAAGGTGCCGGATCCGAAATTCTCCTCTCAGACCAAAGATAAACTG

MIXTH_QC88-366{T} GCGGTGGTTTCCGTGAAGGTGCCGGATCCGAAGTTCTCCTCACAGACCAAAGATAAGCTG

PANRO_LMG26273{T} GCCGTGGTCTCGGTGAAAGTGCCGGATCCGAAGTTCTCCTCACAGACCAAAGACAAGCTG

PANRW_LMG26275{T} GCCGTGGTTTCGGTGAAAGTGCCGGATCCGAAGTTCTCTTCACAGACCAAAGACAAGCTG

PANCY_LMG2657{T} GCCGTGGTATCGGTAAAAGTGCCGGACCCGAAATTCTCCTCGCAGACCAAAGACAAGCTG

PANEU_LMG5346{T} GCCGTGGTTTCCGTGAAAGTGCCGGATCCGAAGTTCTCCTCGCAGACCAAAGATAAGCTG

PANWA_LMG26277{T} GCGGTTGTCTCGGTTAAAGTGCCCGATCCTAAGTTCTCCTCACAGACCAAAGACAAGCTG

PANDI_CCUG25232{T} GCAGTGGTTTCGGTCAAGGTGCCGGATCCGAAATTCTCTTCTCAGACCAAAGACAAGCTG

PANSE_LMG5345{T} GCGGTCGTGTCGGTTAAAGTGCCGGATCCGAAGTTCTCTTCGCAGACCAAAGACAAGCTG

PANBR_LMG5343{T} GCAGTGGTTTCGGTGAAAGTGCCGGATCCAAAATTCTCTTCACAGACCAAAGACAAACTG

PANCO_LMG24534{T} GCCGTGGTTTCGGTGAAAGTACCGGATCCAAAATTCTCTTCACAGACCAAAGATAAACTG

PANAN_LMG2558{T} GCCGTCGTGTCGGTGAAAGTACCGGACCCTAAATTCTCCTCACAGACCAAAGACAAACTG

PANDE_LMG24200{T} GCCGTGGTTTCGGTAAAAGTGCCGGATCCGAAATTCTCCTCACAGACCAAAGATAAGCTG

PANVA_LMG24199{T} GCCGTGGTTTCGGTGAAAGTGCCGGATCCAAAATTCTCCTCACAGACCAAAGACAAACTG

PANEU_LMG24197{T} GCCGTAGTGTCGGTGAAAGTGCCGGATCCAAAATTCTCCTCGCAGACCAAAGATAAACTG

PANAG_DSM3493{T} GCTGTGGTTTCGGTGAAAGTGCCGGATCCTAAATTCTCCTCGCAGACCAAAGATAAACTG

PANAG_CFBP13505 GCTGTGGTTTCGGTGAAAGTGCCGGATCCTAAATTCTCCTCGCAGACCAAAGATAAACTG

TATCI_DSM13699{T} GCCGTGGTATCGGTCAAGGTTCCGGATCCCAAATTCTCGTCTCAGACCAAAGATAAACTG

TATMO_LMG23360{T} GCCGTCGTGTCGGTGAAGGTACCGGATCCAAAATTCTCCTCTCAGACCAAAGATAAACTG

TATPT_ATCC33301{T} GCGGTTGTGTCGGTGAAAGTGCCGGACCCGAAATTCTCTTCCCAGACCAAGGATAAGCTG

TATSA_NML06-3099{T} GCCGTAGTGTCGGTAAAAGTCCCAGACCCGAAATTCTCTTCACAGACCAAAGATAAGCTG

** ** ** ** ** ** ** ** ** ** ** ***** ** ******** ** ** ***

PANST_CCUG26359{T} GTGTCTTCTGAAGTGAAATCGGCCGTTGAGCAGCAGATGAATGAACTGCTGGCGGAATAT

PANST_LMG2632{PT} GTGTCTTCTGAAGTGAAATCGGCCGTTGAGCAGCAGATGAATGAACTGCTGGCGGAATAT

PANAL_LMG24248{T} GTTTCTTCAGAAGTGAAATCGGCCGTTGAACAGCAGATGAACGAACTGCTGGCTGAATAT

PANAN_LMG2665{T} GTTTCTTCAGAAGTGAAATCGGCCGTTGAGCAGCAAATGAATGAACTGCTGGCTGAATAT

PANAN_97-1 GTTTCTTCAGAAGTGAAATCGGCCGTTGAGCAGCAAATGAATGAACTGCTGGCTGAATAT

PANAN_LMG5342 GTTTCTTCAGAAGTGAAATCGGCCGTTGAGCAGCAAATGAATGAACTGCTGGCTGAATAT

PANAN_LMG20103 GTTTCTTCAGAAGTGAAATCGGCCGTTGAGCAGCAAATGAATGAACTGCTGGCTGAATAT

PANAN_NN08200 GTTTCTTCAGAAGTGAAATCGGCCGTTGAGCAGCAAATGAATGAACTGCTGGCTGAATAT

PANAN_ARC311 GTTTCTTCAGAAGTGAAATCGGCCGTTGAGCAGCAAATGAATGAACTACTGGCTGAATAT

PANAN_RSA47 GTTTCTTCAGAAGTGAAATCGGCCGTTGAACAGCAAATGAATGAACTGCTGGCTGAATAT

PANAN_SGAir0210 GTTTCTTCAGAAGTGAAATCGGCCGTTGAACAGCAAATGAATGAACTGCTGGCTGAATAT

MIXCA_DSM22759{T} GTTTCTTCCGAGGTGAAATCGGCGGTTGAATCGCAGATGAACGAACTGCTGGCGGAATAC

MIXGA_DSM22758{T} GTCTCTTCCGAGGTGAAATCGGCGGTTGAATCGCAAATGAACGAACTGCTGGCGGAATAC

MIXAL_LTYR-11Z{T} GTCTCTTCCGAGGTGAAATCGGCAGTTGAATCGCAGATGAACGAGCTGCTGGCGGAATAC

MIXTH_QC88-366{T} GTCTCTTCCGAGGTGAAGTCGGCGGTTGAATCGCAGATGAACGAACTGCTGGCCGAATAC

PANRO_LMG26273{T} GTCTCTTCTGAGGTGAAATCCGCGGTTGAGCAGCAGATGAACGAACTGCTGAGCGAATAC

PANRW_LMG26275{T} GTCTCCTCTGAGGTTAAATCGGCGGTTGAGCAGCAGATGAACGAACTGCTGAGCGAATAC

PANCY_LMG2657{T} GTTTCCTCAGAAGTGAAATCGGCGGTTGAACAGCAGATGAACGAACTGCTGGCTGAGTAC

PANEU_LMG5346{T} GTCTCCTCCGAAGTAAAATCGGCGGTAGAGCAGCAGATGAATGAGCTGCTGAGCGAATAC

PANWA_LMG26277{T} GTCTCCTCTGAGGTGAAATCGGCGGTTGAGCAGCAGATGAACGAATTGCTGAGCGAATAC

PANDI_CCUG25232{T} GTCTCTTCCGAGGTGAAATCGGCGGTGGAACAGCAGATGAACGAACTGCTGAGCGAATAC

PANSE_LMG5345{T} GTCTCGTCCGAGGTGAAATCGGCGGTGGAACAGCAGATGAACGAACTGCTGAGCGAATAC

PANBR_LMG5343{T} GTTTCGTCTGAAGTGAAATCAGCGGTTGAACAGCAGATGAACGAACTGCTGGCGGAATAC

PANCO_LMG24534{T} GTCTCGTCTGAAGTGAAGTCGGCGGTTGAGCAGCAGATGAACGAACTGCTGGCGGAGTAT

PANAN_LMG2558{T} GTTTCGTCAGAGGTGAAATCGGCGGTTGAGCAGCAGATGAACGAACTGCTGGCGGAATAC

PANDE_LMG24200{T} GTCTCGTCCGAGGTGAAATCGGCCGTTGAGCAGCAGATGAACGAACTGCTGGCCGAATAC

PANVA_LMG24199{T} GTCTCGTCAGAGGTGAAATCGGCGGTTGAGCAGCAGATGAACGAACTGCTGGCAGAATAC

PANEU_LMG24197{T} GTCTCCTCCGAGGTGAAATCGGCGGTTGAGCAGCAGATGAATGAGCTGCTGGCAGAATAC

PANAG_DSM3493{T} GTCTCCTCCGAGGTGAAAACAGCGGTTGAGCAGCAGATGAATGAACTGCTGGCTGAATAC

PANAG_CFBP13505 GTCTCCTCCGAGGTGAAAACAGCAGTTGAGCAGCAGATGAATGAACTGCTGGCTGAATAC

TATCI_DSM13699{T} GTCTCTTCGGAAGTGAAATCTGCGGTTGAATCGCAGATGAACGAATTACTGGCAGAGTAC

TATMO_LMG23360{T} GTCTCTTCGGAGGTAAAATCTGCGGTTGAGTCGCAGATGAATGAATTACTGGCAGAATAC

TATPT_ATCC33301{T} GTCTCTTCGGAAGTGAAATCGGCAGTGGAATCGCAGATGAATGAACTGCTCTCTGAATAT

TATSA_NML06-3099{T} GTCTCCTCTGAGGTGAAATCGGCGGTGGAATCGCAGATGAACGAACTGCTGGCCGAATAC

** ** ** ** ** ** * ** ** ** *** ***** ** * ** ** **

PANST_CCUG26359{T} CTGCTGGAAAATCCGGGTGATGCAAAAATCGTTGTCGGCAAAATCATTGATGCCGCCCGC

PANST_LMG2632{PT} CTGCTGGAAAATCCGGGCGATGCAAAAATCGTTGTCGGCAAAATCATTGATGCCGCCCGC

PANAL_LMG24248{T} CTGCTGGAAAATCCGGGCGATGCGAAAATCGTTGTCGGCAAAATTATTGATGCTGCACGC

PANAN_LMG2665{T} CTGCTGGAAAATCCGGGCGATGCAAAAATCGTTGTGGGCAAAATTATTGACGCCGCACGC

PANAN_97-1 CTGCTGGAAAATCCGGGCGATGCAAAAATCGTTGTGGGCAAAATTATTGACGCCGCACGC

PANAN_LMG5342 CTGCTGGAAAATCCGGGCGATGCAAAAATCGTTGTGGGCAAAATTATTGACGCCGCACGC

PANAN_LMG20103 CTGCTGGAAAATCCGGGCGATGCAAAAATCGTTGTGGGCAAAATTATTGACGCCGCACGC

PANAN_NN08200 CTGCTGGAAAATCCGGGCGATGCAAAAATCGTTGTGGGCAAAATTATTGACGCCGCACGC

PANAN_ARC311 CTGCTGGAAAATCCGGGCGATGCAAAAATCGTTGTGGGCAAAATTATTGACGCCGCACGC

PANAN_RSA47 CTGCTGGAAAATCCGGGCGATGCAAAAATCGTTGTGGGCAAAATTATTGACGCCGCACGC

PANAN_SGAir0210 CTGCTGGAAAATCCGGGCGATGCAAAAATCGTTGTGGGCAAAATTATTGACGCCGCACGC

MIXCA_DSM22759{T} CTGCTGGAAAACCCGTCCGATGCGAAAATCGTGGTCGGCAAAATTATTGATGCGGCGCGC

MIXGA_DSM22758{T} CTGCTGGAAAACCCGTCCGATGCGAAAATCGTGGTCGGTAAAATTATCGATGCGGCGCGC

MIXAL_LTYR-11Z{T} CTGCTGGAAAACCCCTCCGATGCGAAAATCGTCGTTGGCAAAATTATCGATGCGGCGCGC

MIXTH_QC88-366{T} CTGCTGGAAAACCCGTCCGATGCGAAAATCGTGGTGGGCAAAATTATCGATGCGGCGCGT

PANRO_LMG26273{T} CTGCTGGAAAACCCATCAGACGCCAAAATCGTAGTCGGTAAAATTATCGATGCGGCCCGT

PANRW_LMG26275{T} CTGCTGGAAAACCCGTCAGACGCGAAAATCGTGGTTGGCAAAATTATTGATGCTGCGCGT

PANCY_LMG2657{T} CTGCTGGAAAACCCGTCTGACGCGAAAATCGTCGTCGGTAAAATTATTGATGCCGCGCGT

PANEU_LMG5346{T} CTGCTGGAAAACCCGGCGGATGCAAAAATTGTGGTAGGCAAAATCATCGATGCGGCGCGC

PANWA_LMG26277{T} CTGCTGGAAAATCCGTCAGACGCCAAAATCGTTGTCGGCAAAATTATCGATGCAGCACGC

PANDI_CCUG25232{T} CTGCTGGAAAATCCGTCTGACGCCAAAATCGTGGTCGGCAAAATTATCGATGCCGCGCGT

PANSE_LMG5345{T} CTGCTGGAAAACCCGAGCGACGCCAAAATTGTCGTCGGCAAGATCATTGACGCCGCCCGC

PANBR_LMG5343{T} CTGCTGGAAAATCCGGGTGACGCAAAAATCGTGGTCGGCAAAATCATCGACGCAGCGCGC

PANCO_LMG24534{T} CTGCTGGAAAACCCGTCAGACGCCAAAATCGTGGTGGGCAAAATCATCGACGCCGCCCGC

PANAN_LMG2558{T} CTGCTGGAAAACCCGGCAGACGCCAAAATCGTGGTCGGTAAAATCATCGACGCCGCGCGC

PANDE_LMG24200{T} CTGCTGGAAAACCCGGCAGACGCCAAAATTGTGGTCGGCAAAATCATTGACGCCGCCCGT

PANVA_LMG24199{T} CTGCTGGAAAACCCGTCAGACGCCAAAATCGTCGTGGGCAAAATTATCGATGCTGCCCGT

PANEU_LMG24197{T} CTGCTGGAAAATCCATCAGACGCCAAAATCGTCGTCGGCAAAATCATCGACGCAGCCCGT

PANAG_DSM3493{T} CTGCTGGAAAATCCTTCAGACGCCAAAATTGTAGTCGGCAAAATCATCGACGCTGCCCGT

PANAG_CFBP13505 CTGCTGGAAAACCCATCAGACGCCAAAATTGTAGTCGGCAAAATCATCGACGCCGCCCGT

TATCI_DSM13699{T} CTGCTGGAGAATCCGTCTGATGCGAAAATTGTGGTCGGTAAAATTATTGATGCGGCTCGT

TATMO_LMG23360{T} CTGCTGGAAAATCCGTCTGATGCGAAAATTGTGGTCGGAAAAATTATCGATGCGGCCCGT

TATPT_ATCC33301{T} CTGCTGGAAAATCCGTCCGATGCCAAGATTGTCGTCGGTAAAATTATCGATGCTGCCCGT

TATSA_NML06-3099{T} CTGCTGGAAAACCCGGGTGATGCAAAAATCGTGGTGGGTAAAATTATCGATGCGGCCCGT

******** ** ** ** ** ** ** ** ** ** ** ** ** ** ** ** **

PANST_CCUG26359{T} GCCCGTGAAGCCGCGCGCCGTGCCCGTGAAATGACCCGCCGCAAAGGTGCGCTGGATCTG

PANST_LMG2632{PT} GCCCGTGAAGCCGCGCGCCGTGCCCGTGAAATGACACGCCGCAAAGGTGCGCTGGATCTG

PANAL_LMG24248{T} GCCCGTGAAGCTGCACGTCGTGCCCGTGAGATGACCCGCCGTAAAGGCGCGCTGGATCTT

PANAN_LMG2665{T} GCTCGTGAAGCCGCACGCCGTGCCCGTGAGATGACCCGCCGTAAAGGCGCGCTGGATCTT

PANAN_97-1 GCTCGTGAAGCCGCACGCCGTGCCCGTGAGATGACCCGCCGTAAAGGCGCGCTGGATCTT

PANAN_LMG5342 GCTCGTGAAGCTGCACGCCGTGCCCGTGAGATGACCCGCCGTAAAGGCGCGCTGGATCTT

PANAN_LMG20103 GCTCGTGAAGCCGCACGCCGTGCCCGTGAGATGACCCGCCGTAAAGGCGCGCTGGATCTT

PANAN_NN08200 GCTCGTGAAGCTGCACGCCGTGCCCGTGAGATGACCCGCCGTAAAGGCGCGCTGGATCTT

PANAN_ARC311 GCGCGTGAAGCGGCACGCCGTGCCCGTGAAATGACCCGCCGTAAAGGCGCGCTGGATCTT

PANAN_RSA47 GCGCGTGAAGCGGCACGCCGTGCCCGTGAAATGACCCGCCGCAAAGGCGCGCTGGATCTT

PANAN_SGAir0210 GCGCGTGAAGCGGCACGCCGTGCCCGTGAAATGACCCGCCGCAAAGGCGCGCTGGATCTT

MIXCA_DSM22759{T} GCGCGCGAAGCAGCCCGTCGCGCCCGTGAAATGACCCGTCGTAAAGGCGCGCTGGATCTG

MIXGA_DSM22758{T} GCGCGCGAAGCGGCGCGTCGCGCCCGTGAAATGACCCGTCGTAAAGGCGCGTTGGATCTG

MIXAL_LTYR-11Z{T} GCGCGCGAAGCGGCACGTCGTGCACGTGAAATGACCCGTCGTAAAGGCGCGCTGGATCTG

MIXTH_QC88-366{T} GCGCGTGAAGCAGCGCGCCGTGCGCGTGAAATGACCCGCCGTAAAGGCGCGCTGGACCTG

PANRO_LMG26273{T} GCGCGTGAAGCGGCTCGTCGTGCTCGTGAAATGACGCGTCGTAAAGGCGCACTGGATTTG

PANRW_LMG26275{T} GCACGTGAAGCGGCACGCCGCGCACGTGAAATGACTCGCCGTAAAGGCGCACTGGATTTG

PANCY_LMG2657{T} GCGCGTGAAGCGGCACGTCGTGCCCGTGAAATGACCCGCCGTAAAGGCGCGCTGGATCTG

PANEU_LMG5346{T} GCGCGCGAAGCAGCACGCCGTGCGCGTGAGATGACCCGCCGCAAAGGCGCGCTGGATCTG

PANWA_LMG26277{T} GCGCGTGAAGCGGCGCGTCGTGCACGTGAAATGACCCGCCGTAAAGGCGCGCTGGATCTG

PANDI_CCUG25232{T} GCGCGTGAAGCCGCACGTCGTGCACGTGAAATGACCCGCCGTAAAGGCGCGCTGGACCTG

PANSE_LMG5345{T} GCCCGTGAAGCGGCCCGCCGCGCTCGCGAAATGACGCGCCGTAAGGGCGCGCTCGATCTG

PANBR_LMG5343{T} GCCCGTGAAGCCGCACGTCGTGCCCGTGAAATGACCCGTCGTAAAGGCGCGCTGGACCTG

PANCO_LMG24534{T} GCGCGTGAAGCCGCGCGTCGTGCGCGTGAAATGACCCGTCGTAAAGGCGCGCTGGACCTG

PANAN_LMG2558{T} GCCCGTGAAGCGGCCCGTCGCGCCCGCGAGATGACCCGCCGTAAAGGCGCGCTGGATCTG

PANDE_LMG24200{T} GCCCGTGAAGCGGCGCGTCGCGCCCGTGAAATGACCCGCCGTAAGGGCGCGCTGGATCTG

PANVA_LMG24199{T} GCCCGTGAAGCAGCACGTCGCGCCCGTGAAATGACCCGCCGTAAAGGCGCGCTGGATCTG

PANEU_LMG24197{T} GCCCGTGAAGCGGCCCGCCGCGCCCGTGAAATGACCCGCCGTAAAGGCGCGCTGGATCTG

PANAG_DSM3493{T} GCCCGTGAAGCGGCACGCCGCGCCCGTGAAATGACCCGCCGTAAAGGCGCGCTGGACCTG

PANAG_CFBP13505 GCCCGTGAAGCGGCACGCCGTGCCCGTGAAATGACCCGCCGTAAAGGCGCGCTGGACCTG

TATCI_DSM13699{T} GCCCGTGAAGCGGCCCGTCGCGCCCGTGAAATGACCCGCCGTAAAGGTGCACTGGATCTG

TATMO_LMG23360{T} GCCCGTGAAGCAGCCCGACGTGCTCGTGAAATGACCCGCCGTAAAGGTGCTCTGGATCTG

TATPT_ATCC33301{T} GCGCGTGAAGCGGCGCGCCGTGCCCGTGAAATGACCCGTCGTAAAGGCGCGCTGGACCTG

TATSA_NML06-3099{T} GCCCGTGAAGCCGCTCGTCGTGCCCGTGAGATGACCCGCCGTAAAGGGGCGCTGGATTTG

** ** ***** ** ** ** ** ** ** ***** ** ** ** ** ** * ** *

PANST_CCUG26359{T} GCTGGCCTGCCGGGCAAGCTGGCGGATTGTCAGGAGCGCGATCCGGCTCTGTCTGAAATC

PANST_LMG2632{PT} GCTGGCCTGCCGGGCAAGCTGGCGGATTGTCAGGAGCGCGATCCGGCTCTGTCTGAAATC

PANAL_LMG24248{T} GCTGGCCTGCCAGGCAAACTGGCGGATTGTCAGGAGCGCGATCCGGCGCTGTCAGAAATC

PANAN_LMG2665{T} GCTGGCCTGCCAGGCAAACTGGCGGACTGCCAGGAGCGCGACCCGGCGCTGTCAGAAATT

PANAN_97-1 GCTGGCCTGCCAGGCAAACTGGCGGACTGTCAGGAGCGCGACCCGGCGCTGTCAGAAATT

PANAN_LMG5342 GCTGGCCTGCCAGGCAAACTGGCGGACTGCCAGGAACGCGACCCGGCGCTGTCAGAAATT

PANAN_LMG20103 GCTGGCCTGCCAGGCAAACTGGCGGACTGCCAGGAGCGCGACCCGGCGCTGTCAGAAATT

PANAN_NN08200 GCTGGCCTGCCAGGCAAACTGGCGGACTGCCAGGAACGCGACCCGGCGCTGTCAGAAATT

PANAN_ARC311 GCTGGCCTGCCAGGCAAACTGGCGGACTGCCAGGAGCGCGACCCGGCGCTGTCAGAAATT

PANAN_RSA47 GCTGGCCTGCCAGGCAAACTGGCGGACTGCCAGGAGCGCGACCCGGCGCTGTCAGAAATT

PANAN_SGAir0210 GCTGGCCTGCCAGGCAAACTGGCGGACTGCCAGGAGCGCGACCCGGCGCTGTCAGAAATT

MIXCA_DSM22759{T} GCGGGCCTGCCGGGCAAACTGGCCGACTGTCAGGAGCGCGATCCGGCGCTGTCGGAAATC

MIXGA_DSM22758{T} GCGGGCCTGCCGGGCAAGCTGGCTGACTGTCAGGAACGCGATCCGGCGCTGTCGGAAATC

MIXAL_LTYR-11Z{T} GCCGGTCTGCCGGGCAAACTGGCCGACTGTCAGGAGCGCGATCCGGCGCACTCAGAAATT

MIXTH_QC88-366{T} GCCGGGCTGCCGGGCAAGCTGGCCGACTGTCAGGAGCGCGATCCGGCGCACTCTGAAATT

PANRO_LMG26273{T} GCTGGCCTGCCGGGCAAACTGGCAGATTGCCAGGAACGTGACCCTGCTCTGTCTGAAATC

PANRW_LMG26275{T} GCTGGTCTGCCGGGCAAGCTGGCAGATTGCCAGGAACGCGACCCTGCACTGTCTGAAATC

PANCY_LMG2657{T} GCCGGTCTGCCGGGCAAACTGGCAGATTGCCAGGAACGCGACCCGGCGCTGTCCGAAATC

PANEU_LMG5346{T} GCCGGCCTGCCGGGCAAGCTGGCAGACTGTCAGGAGCGCGATCCGGCGCTCTCTGAAATC

PANWA_LMG26277{T} GCTGGCCTGCCGGGCAAGCTGGCGGACTGTCAGGAGCGCGATCCGGCGCTGTCTGAAATC

PANDI_CCUG25232{T} GCGGGCCTGCCGGGCAAGCTGGCGGACTGTCAGGAGCGCGATCCGGCGCTGTCTGAAATC

PANSE_LMG5345{T} GCGGGTCTGCCGGGCAAGCTGGCGGATTGCCAGGAGCGCGATCCGGCGCTCTCTGAAATC

PANBR_LMG5343{T} GCGGGTCTGCCAGGCAAACTGGCAGATTGCCAGGAGCGCGACCCGGCGCTGTCTGAAATC

PANCO_LMG24534{T} GCCGGTCTGCCAGGCAAACTGGCGGATTGCCAGGAACGCGACCCGGCGCTGTCAGAAATT

PANAN_LMG2558{T} GCTGGCCTGCCAGGCAAACTGGCGGATTGCCAGGAGCGCGACCCGGCGCTCTCTGAAATC

PANDE_LMG24200{T} GCCGGTCTGCCAGGCAAACTGGCGGATTGCCAGGAGCGCGACCCGGCGCTCTCTGAAATC

PANVA_LMG24199{T} GCTGGTCTGCCAGGCAAACTGGCGGATTGCCAGGAGCGCGACCCGGCGCTGTCTGAAATC

PANEU_LMG24197{T} GCAGGTCTGCCAGGCAAACTGGCGGATTGCCAGGAGCGCGACCCGGCGTTGTCCGAAATC

PANAG_DSM3493{T} GCGGGTCTGCCAGGCAAACTGGCGGATTGCCAGGAGCGCGACCCGGCGCTGTCTGAAATC

PANAG_CFBP13505 GCGGGTCTGCCAGGAAAACTGGCGGATTGCCAGGAGCGCGACCCGGCGCTGTCTGAAATC

TATCI_DSM13699{T} GCTGGCTTGCCGGGCAAACTGGCCGATTGCCAGGAACGTGATCCTGCATTCTCCGAAGTT

TATMO_LMG23360{T} GCTGGTTTGCCGGGCAAACTGGCGGACTGCCAGGAGCGTGATCCTGCATTCTCTGAAGTT

TATPT_ATCC33301{T} GCCGGATTACCGGGCAAACTGGCGGATTGCCAGGAACGCGACCCTGCGCTTTCAGAAGTT

TATSA_NML06-3099{T} GCCGGTTTACCAGGCAAGCTGGCGGACTGTCAGGAGCGCGATCCGGCCCTGTCCGAAGTG

** ** * ** ** ** ***** ** ** ***** ** ** ** ** ** *** *

PANST_CCUG26359{T} TACCTGGTGGAGGGTGACTCCGCGGGCGGTTCTGCCAAGCAAGGCCGTAACCGTAAAAAC

PANST_LMG2632{PT} TACCTGGTGGAGGGTGACTCCGCGGGCGGTTCTGCCAAGCAAGGCCGTAACCGTAAAAAC

PANAL_LMG24248{T} TACCTGGTGGAGGGTGATTCCGCAGGCGGTTCTGCCAAGCAAGGCCGTAACCGTAAGAAT

PANAN_LMG2665{T} TACCTGGTGGAGGGTGACTCTGCTGGCGGTTCTGCCAAGCAAGGCCGCAACCGTAAGAAT

PANAN_97-1 TACCTGGTGGAGGGTGACTCTGCGGGCGGTTCTGCCAAGCAAGGCCGCAACCGTAAGAAT

PANAN_LMG5342 TACCTGGTGGAGGGTGACTCTGCGGGCGGTTCTGCCAAGCAAGGCCGGAACCGTAAGAAT

PANAN_LMG20103 TACCTGGTGGAGGGTGACTCTGCGGGCGGTTCTGCCAAGCAAGGCCGCAACCGTAAGAAT

PANAN_NN08200 TACCTGGTGGAGGGTGACTCTGCGGGCGGTTCTGCCAAGCAAGGCCGGAACCGTAAGAAT

PANAN_ARC311 TACCTGGTGGAGGGTGACTCTGCGGGCGGTTCTGCCAAGCAAGGCCGCAACCGTAAGAAT

PANAN_RSA47 TACCTGGTGGAGGGTGACTCAGCGGGCGGTTCTGCCAAGCAAGGCCGCAACCGTAAAAAT

PANAN_SGAir0210 TACCTGGTGGAGGGTGACTCAGCGGGCGGTTCTGCCAAGCAAGGCCGCAACCGTAAAAAT

MIXCA_DSM22759{T} TACCTGGTGGAGGGTGACTCCGCAGGCGGTTCCGCCAAGCAGGGCCGTAACCGTAAGAAC

MIXGA_DSM22758{T} TATCTGGTGGAGGGTGACTCCGCAGGCGGTTCCGCCAAACAGGGCCGTAACCGTAAGAAC

MIXAL_LTYR-11Z{T} TACCTGGTGGAGGGTGACTCCGCAGGCGGTTCAGCCAAGCAGGGTCGTAACCGTAAGAAT

MIXTH_QC88-366{T} TACCTGGTGGAGGGTGACTCCGCAGGGGGTTCAGCCAAGCAGGGACGTAACCGTAAGAAC

PANRO_LMG26273{T} TACTTAGTGGAAGGGGACTCAGCGGGCGGCTCTGCCAAGCAGGGACGTAACCGTAAGAAT

PANRW_LMG26275{T} TACTTAGTGGAAGGGGACTCAGCGGGCGGCTCTGCGAAGCAGGGACGTAACCGTAAGAAC

PANCY_LMG2657{T} TACCTGGTGGAGGGTGACTCCGCAGGCGGTTCCGCCAAACAGGGCCGTAACCGTAAGAAC

PANEU_LMG5346{T} TACCTGGTGGAGGGTGACTCTGCAGGCGGATCGGCGAAGCAGGGGCGTAACCGTAAAAAC

PANWA_LMG26277{T} TACCTGGTGGAGGGTGACTCTGCGGGCGGTTCAGCCAAGCAGGGTCGTAACCGTAAAAAC

PANDI_CCUG25232{T} TACCTGGTGGAGGGTGACTCTGCAGGCGGTTCCGCCAAGCAGGGACGTAACCGTAAGAAC

PANSE_LMG5345{T} TACCTGGTGGAGGGTGACTCCGCAGGCGGTTCGGCCAAACAGGGCCGTAACCGTAAGAAC

PANBR_LMG5343{T} TACCTGGTGGAGGGTGACTCCGCTGGCGGTTCGGCCAAACAGGGCCGTAACCGTAAGAAT

PANCO_LMG24534{T} TACCTGGTGGAGGGTGACTCCGCTGGCGGTTCGGCTAAACAGGGCCGTAACCGTAAAAAT

PANAN_LMG2558{T} TACCTGGTGGAGGGTGACTCCGCAGGCGGCTCGGCCAAACAGGGCCGTAACCGTAAAAAC

PANDE_LMG24200{T} TACCTGGTGGAGGGTGACTCCGCAGGCGGCTCGGCCAAACAGGGCCGTAACCGTAAAAAC

PANVA_LMG24199{T} TACCTGGTGGAGGGTGACTCCGCAGGCGGCTCGGCCAAACAGGGCCGTAACCGTAAAAAC

PANEU_LMG24197{T} TACCTGGTGGAGGGTGACTCCGCAGGCGGCTCGGCTAAACAGGGCCGTAACCGTAAGAAC

PANAG_DSM3493{T} TACCTGGTGGAGGGTGACTCCGCAGGCGGCTCGGCCAAACAGGGCCGTAACCGTAAAAAC

PANAG_CFBP13505 TACCTGGTGGAGGGTGACTCCGCAGGCGGCTCGGCCAAACAGGGCCGTAACCGTAAAAAC

TATCI_DSM13699{T} TACCTGGTGGAAGGGGACTCTGCTGGCGGATCAGCCAAGCAGGGGCGTAACCGTAAAAAC

TATMO_LMG23360{T} TACCTGGTGGAAGGGGACTCTGCTGGCGGCTCTGCCAAGCAGGGGCGTAACCGTAAAAAC

TATPT_ATCC33301{T} TATCTGGTGGAAGGGGACTCTGCGGGCGGCTCTGCGAAGCAGGGGCGTAACCGCAAGAAT

TATSA_NML06-3099{T} TACTTGGTGGAAGGGGACTCCGCGGGTGGTTCTGCCAAGCAGGGCCGTAACCGTAAAAAC

** * ***** ** ** ** ** ** ** ** ** ** ** ** ** ***** ** **

PANST_CCUG26359{T} CAGGCTATTCTGCCGCTGAAAGGGAAAATTCTGAACGTTGAGAAGGCGCGTTTCGATAAG

PANST_LMG2632{PT} CAGGCTATTCTGCCGCTGAAAGGGAAAATTCTGAACGTTGAGAAGGCGCGTTTCGATAAG

PANAL_LMG24248{T} CAGGCCATTCTGCCACTTAAAGGTAAGATCCTGAACGTCGAGAAGGCGCGCTTCGATAAG

PANAN_LMG2665{T} CAGGCCATTCTGCCGCTGAAAGGTAAGATCCTGAACGTTGAGAAGGCGCGTTTCGATAAG

PANAN_97-1 CAGGCCATTCTGCCGCTTAAAGGTAAGATCCTGAACGTTGAGAAGGCGCGTTTCGATAAG

PANAN_LMG5342 CAGGCCATTCTGCCGCTGAAAGGTAAGATCCTGAACGTTGAGAAGGCGCGTTTCGATAAG

PANAN_LMG20103 CAGGCCATTCTGCCGCTGAAAGGTAAGATCCTGAACGTTGAGAAGGCGCGTTTCGATAAG

PANAN_NN08200 CAGGCCATTCTGCCGCTGAAAGGTAAGATCCTGAACGTTGAGAAGGCGCGTTTCGATAAG

PANAN_ARC311 CAGGCCATTCTGCCGCTGAAAGGTAAGATCCTGAACGTTGAGAAGGCGCGTTTCGATAAG

PANAN_RSA47 CAGGCCATTCTGCCGCTGAAAGGTAAGATCCTGAACGTTGAGAAGGCGCGTTTCGATAAG

PANAN_SGAir0210 CAGGCCATTCTGCCGCTGAAAGGTAAGATCCTGAACGTTGAGAAGGCGCGTTTCGATAAG

MIXCA_DSM22759{T} CAGGCGATTCTGCCGCTAAAAGGTAAAATCCTGAACGTGGAGAAGGCGCGCTTCGACAAG

MIXGA_DSM22758{T} CAGGCGATTCTGCCGCTGAAGGGGAAAATCCTGAACGTGGAGAAGGCGCGCTTCGACAAG

MIXAL_LTYR-11Z{T} CAGGCGATTCTGCCGCTGAAGGGTAAAATCCTGAACGTAGAGAAAGCGCGCTTCGATAAG

MIXTH_QC88-366{T} CAGGCCATCCTGCCGCTGAAAGGTAAAATCCTGAACGTAGAGAAAGCGCGCTTCGATAAG

PANRO_LMG26273{T} CAGGCGATTCTGCCACTGAAAGGTAAAATCCTGAACGTGGAGAAAGCGCGTTTCGACAAG

PANRW_LMG26275{T} CAGGCGATCCTGCCGTTGAAAGGTAAAATCCTCAACGTTGAGAAAGCGCGTTTCGATAAG

PANCY_LMG2657{T} CAGGCGATTCTGCCGCTGAAAGGTAAAATCCTTAACGTGGAGAAAGCGCGTTTCGACAAG

PANEU_LMG5346{T} CAGGCGATTCTGCCGCTGAAGGGTAAAATCCTCAACGTAGAGAAAGCGCGTTTCGATAAA

PANWA_LMG26277{T} CAGGCGATTCTGCCGCTGAAAGGGAAGATCCTGAACGTAGAGAAAGCGCGTTTTGATAAA

PANDI_CCUG25232{T} CAGGCGATTCTGCCGCTGAAAGGTAAAATCCTTAACGTGGAGAAAGCGCGTTTCGACAAA

PANSE_LMG5345{T} CAGGCGATTCTGCCGCTGAAGGGCAAAATCCTCAACGTAGAGAAAGCGCGCTTCGACAAG

PANBR_LMG5343{T} CAGGCGATTCTGCCGCTGAAAGGTAAGATCCTTAACGTTGAGAAAGCGCGCTTCGACAAA

PANCO_LMG24534{T} CAGGCGATTCTGCCGCTGAAAGGTAAGATCCTCAACGTTGAGAAAGCGCGCTTCGATAAA

PANAN_LMG2558{T} CAGGCGATCCTGCCGCTGAAAGGTAAGATCCTGAACGTTGAGAAAGCGCGTTTCGATAAG

PANDE_LMG24200{T} CAGGCGATTCTGCCGCTGAAAGGTAAGATCCTGAACGTCGAGAAAGCGCGTTTCGACAAG

PANVA_LMG24199{T} CAGGCGATTCTGCCGCTGAAAGGTAAGATCCTGAACGTTGAGAAAGCGCGTTTCGACAAG

PANEU_LMG24197{T} CAGGCGATTCTGCCGCTGAAAGGTAAAATCCTTAACGTTGAGAAAGCGCGTTTCGACAAG

PANAG_DSM3493{T} CAGGCGATTCTGCCGCTGAAAGGTAAGATTCTTAACGTTGAGAAAGCGCGTTTCGACAAG

PANAG_CFBP13505 CAGGCGATTCTGCCGCTGAAAGGTAAGATTCTTAACGTTGAGAAAGCGCGTTTCGACAAG

TATCI_DSM13699{T} CAGGCAATTTTGCCGCTTAAAGGTAAGATTCTGAACGTCGAAAAAGCGCGTTTCGACAAA

TATMO_LMG23360{T} CAGGCGATTCTGCCGCTAAAAGGTAAAATTCTGAATGTCGAAAAAGCGCGTTTCGACAAA

TATPT_ATCC33301{T} CAGGCCATTTTACCTCTGAAAGGTAAGATCCTGAACGTCGAAAAAGCCCGTTTTGACAAG

TATSA_NML06-3099{T} CAAGCCATATTACCGTTGAAAGGTAAGATCCTGAACGTGGAAAAAGCGCGTTTCGACAAG

** ** ** * ** * ** ** ** ** ** ** ** ** ** ** ** ** ** **

PANST_CCUG26359{T} ATGCTGGCGTCTCAGGAAGTGGCTACGCTGATTACCGCGCTGGGCTGCGGCATTGGCCGC

PANST_LMG2632{PT} ATGCTGGCGTCTCAGGAAGTGGCTACGCTGATTACCGCGCTGGGCTGCGGCATTGGCCGC

PANAL_LMG24248{T} ATGCTGGCTTCTCAGGAAGTGGCAACGCTGATTACCGCGCTCGGCTGCGGCATCGGCCGC

PANAN_LMG2665{T} ATGCTGGCTTCTCAGGAAGTCGCCACGTTGATTACCGCGCTGGGCTGTGGCATCGGCCGC

PANAN_97-1 ATGCTGGCTTCTCAGGAAGTCGCCACGTTGATTACCGCGCTGGGCTGTGGCATCGGCCGC

PANAN_LMG5342 ATGCTGGCTTCTCAGGAAGTCGCCACGTTGATTACCGCGCTGGGCTGTGGCATCGGCCGC

PANAN_LMG20103 ATGCTGGCTTCTCAGGAAGTCGCCACGTTGATTACCGCGCTGGGCTGTGGCATCGGCCGC

PANAN_NN08200 ATGCTGGCTTCTCAGGAAGTCGCCACGTTGATTACCGCGCTGGGCTGTGGCATCGGCCGC

PANAN_ARC311 ATGCTGGCTTCTCAGGAAGTTGCCACGTTGATTACCGCACTGGGCTGTGGCATCGGCCGC

PANAN_RSA47 ATGCTGGCTTCTCAGGAAGTTGCCACGTTGATTACCGCACTGGGCTGTGGCATCGGCCGC

PANAN_SGAir0210 ATGCTGGCTTCTCAGGAAGTTGCCACGTTGATTACCGCACTGGGCTGTGGCATCGGCCGC

MIXCA_DSM22759{T} ATGCTGGCGTCACAGGAAGTCGCTACGCTGATCACCGCGCTGGGCTGCGGCATTGGCCGC

MIXGA_DSM22758{T} ATGCTGGCATCGCAGGAAGTCGCCACGCTGATCACCGCGCTGGGCTGCGGCATCGGCCGT

MIXAL_LTYR-11Z{T} ATGCTCGCTTCGCAGGAAGTGGCAACGCTGATCACCGCGCTGGGCTGCGGCATTGGCCGT

MIXTH_QC88-366{T} ATGCTCGCCTCGCAGGAAGTGGCGACGCTGATCACCGCGCTGGGCTGCGGCATCGGCCGT

PANRO_LMG26273{T} ATGCTCGCTTCGCAGGAAGTCGCCACCCTGATTACCGCACTTGGTTGTGGTATTGGTCGT

PANRW_LMG26275{T} ATGCTCTCTTCTCAGGAAGTGGCTACGCTTATCACCGCACTCGGTTGTGGTATCGGACGC

PANCY_LMG2657{T} ATGCTTTCCTCGCAGGAAGTTGCCACGCTGATTACCGCGCTGGGCTGCGGTATTGGTCGT

PANEU_LMG5346{T} ATGCTCTCTTCGCAGGAAGTCGCCACGCTGATCACCGCGCTGGGCTGCGGTATCGGCCGC

PANWA_LMG26277{T} ATGCTCTCTTCGCAGGAGGTCGCCACGCTGATCACCGCCCTGGGCTGCGGTATCGGTCGC

PANDI_CCUG25232{T} ATGCTCTCTTCACAGGAAGTTGCCACGCTGATCACCGCACTGGGCTGCGGTATCGGCCGC

PANSE_LMG5345{T} ATGCTCTCTTCGCAGGAAGTGGCGACGCTGATCACCGCGCTGGGCTGCGGCATCGGCCGC

PANBR_LMG5343{T} ATGCTCGCCTCGCAGGAAGTGGCCACGCTGATTACCGCGCTCGGTTGCGGCATTGGTCGC

PANCO_LMG24534{T} ATGCTCGCCTCGCAGGAAGTAGCCACGCTGATCACCGCGCTCGGTTGCGGTATTGGTCGC

PANAN_LMG2558{T} ATGCTCGCCTCTCAGGAAGTGGCCACGCTGATCACCGCACTGGGCTGTGGCATTGGCCGC

PANDE_LMG24200{T} ATGCTCGCGTCGCAGGAAGTCGCTACGCTGATCACCGCACTGGGTTGCGGCATTGGTCGC

PANVA_LMG24199{T} ATGCTCGCCTCGCAGGAAGTGGCCACGCTGATCACCGCACTGGGTTGCGGTATTGGTCGC

PANEU_LMG24197{T} ATGCTTGCTTCCCAGGAAGTTGCCACGCTGATCACCGCGCTGGGCTGTGGTATTGGTCGC

PANAG_DSM3493{T} ATGCTCGCCTCCCAGGAAGTGGCCACGCTGATCACCGCGCTGGGCTGCGGTATTGGTCGC

PANAG_CFBP13505 ATGCTCGCCTCCCAGGAAGTGGCCACGCTGATCACCGCGCTGGGCTGCGGTATTGGTCGC

TATCI_DSM13699{T} ATGCTTTCTTCCCAGGAAGTGGCAACACTGATCACGGCGCTGGGTTGTGGTATTGGTCGC

TATMO_LMG23360{T} ATGCTCTCTTCTCAGGAAGTCGCCACTCTGATCACCGCACTGGGTTGCGGTATTGGCCGC

TATPT_ATCC33301{T} ATGCTCTCTTCCCAGGAAGTGGCAACACTGATCACCGCGCTGGGTTGTGGTATCGGCCGC

TATSA_NML06-3099{T} ATGCTTTCTTCGCAGGAGGTGGCAACCCTGATCACCGCATTGGGCTGTGGTATCGGTCGT

***** * ** ***** ** ** ** * ** ** ** * ** ** ** ** ** **

PANST_CCUG26359{T} GACGAATATAACCCGGATAAACTGCGCTATCACAGCATCATCATCATGACCGATGCGGAC

PANST_LMG2632{PT} GACGAATATAACCCGGATAAACTGCGCTATCACAGCATCATCATCATGACCGATGCGGAC

PANAL_LMG24248{T} GATGAATATAATCCAGACAAACTGCGTTATCACAGCATCATCATCATGACCGATGCGGAC

PANAN_LMG2665{T} GATGAATACAATCCGGATAAACTGCGTTACCACAGCATCATCATCATGACCGATGCGGAC

PANAN_97-1 GATGAATACAATCCGGATAAACTGCGTTACCACAGCATCATCATCATGACCGATGCGGAC

PANAN_LMG5342 GATGAATACAATCCGGATAAACTGCGTTACCACAGCATCATCATCATGACCGATGCGGAC

PANAN_LMG20103 GATGAATACAATCCGGATAAACTGCGTTACCACAGCATCATCATCATGACCGATGCGGAC

PANAN_NN08200 GATGAATACAATCCGGATAAACTGCGTTACCACAGCATCATCATCATGACCGATGCGGAC

PANAN_ARC311 GATGAATACAATCCGGATAAACTGCGTTACCACAGCATCATCATCATGACCGATGCGGAC

PANAN_RSA47 GATGAATACAATCCGGATAAACTGCGTTACCACAGCATCATCATCATGACCGATGCGGAC

PANAN_SGAir0210 GATGAATACAATCCAGATAAACTGCGTTACCACAGCATCATCATCATGACCGATGCGGAC

MIXCA_DSM22759{T} GACGAGTACAACCCGGACAAGCTGCGCTATCACAGCATCATTATCATGACCGATGCGGAC

MIXGA_DSM22758{T} GACGAGTACAACCCGGACAAGCTGCGCTATCACAGCATCATCATCATGACCGATGCGGAC

MIXAL_LTYR-11Z{T} GACGAATATAACCCGGACAAGCTGCGTTATCACAGCATCATTATCATGACCGATGCGGAC

MIXTH_QC88-366{T} GACGAATACAACCCGGACAAGCTGCGCTATCACAGCATCATTATCATGACCGATGCGGAC

PANRO_LMG26273{T} GACGAATACAACCCGGATAAACTGCGTTATCACAGCATCATCATCATGACCGATGCGGAC

PANRW_LMG26275{T} GACGAATATAACCCGGACAAACTGCGTTATCACAGCATCATCATCATGACCGATGCTGAC

PANCY_LMG2657{T} GACGAATACAACCCGGACAAACTGCGTTATCACAGCATCATCATCATGACCGATGCGGAC

PANEU_LMG5346{T} GATGAGTACAACCCGGATAAGCTGCGCTATCACAGCATCATTATCATGACCGATGCGGAC

PANWA_LMG26277{T} GATGAGTACAACCCGGACAAGCTGCGCTATCACAGCATCATCATCATGACCGATGCGGAC

PANDI_CCUG25232{T} GATGAGTACAACCCGGACAAGTTGCGCTATCACAGCATCATCATCATGACCGATGCGGAC

PANSE_LMG5345{T} GACGAATACAACCCGGACAAGCTGCGCTATCACAGCATCATCATCATGACCGATGCTGAC

PANBR_LMG5343{T} GACGAGTACAACCCGGACAAGCTGCGCTATCACAGCATCATCATCATGACCGATGCCGAT

PANCO_LMG24534{T} GACGAGTACAACCCGGACAAGCTGCGCTATCACAGCATCATCATCATGACCGATGCCGAT

PANAN_LMG2558{T} GACGAATACAACCCGGACAAGCTGCGCTATCACAGCATCATCATCATGACCGATGCCGAC

PANDE_LMG24200{T} GACGAGTACAACCCGGACAAGCTGCGCTATCACAGCATCATCATCATGACCGATGCCGAC

PANVA_LMG24199{T} GATGAATACAATCCGGACAAACTGCGCTATCACAGCATCATCATCATGACCGATGCCGAC

PANEU_LMG24197{T} GATGAGTACAACCCGGACAAGCTGCGTTATCACAGCATCATCATCATGACCGATGCCGAC

PANAG_DSM3493{T} GACGAGTACAACCCGGACAAGCTGCGCTATCACAGCATCATCATCATGACCGATGCCGAC

PANAG_CFBP13505 GACGAGTACAACCCGGACAAACTGCGCTATCACAGCATCATCATCATGACCGATGCCGAC

TATCI_DSM13699{T} GATGAATACAATCCGGATAAATTGCGTTACCACAGCATTATTATCATGACGGATGCGGAC

TATMO_LMG23360{T} GATGAGTACAATCCGGACAAATTGCGTTACCACAGCATCATTATCATGACGGATGCGGAC

TATPT_ATCC33301{T} GATGAGTACAATCCGGACAAATTGCGTTATCACAGTATCATTATTATGACGGATGCGGAC

TATSA_NML06-3099{T} GATGAGTACAACCCCGATAAACTGCGTTATCACAGCATCATCATCATGACGGATGCGGAC

** ** ** ** ** ** ** **** ** ***** ** ** ** ***** ***** **

PANST_CCUG26359{T} GTCGACGGCTCGCACATTCGTACACTGCTGTTGACCTTTTTCTATCGTCAGATGCCAGAA

PANST_LMG2632{PT} GTCGACGGCTCGCACATTCGTACACTGCTGTTGACCTTTTTCTATCGTCAGATGCCAGAA

PANAL_LMG24248{T} GTCGACGGCTCACACATTCGTACGCTGCTGTTGACCTTCTTCTATCGCCAGATGCCAGAA

PANAN_LMG2665{T} GTCGACGGCTCCCACATTCGCACACTGCTGTTGACCTTCTTCTATCGTCAGATGCCAGAG

PANAN_97-1 GTCGACGGCTCCCACATTCGTACACTGCTGTTGACCTTCTTCTATCGTCAGATGCCAGAG

PANAN_LMG5342 GTCGACGGCTCCCACATTCGTACACTGCTGTTGACCTTCTTCTATCGTCAGATGCCAGAG

PANAN_LMG20103 GTCGACGGCTCCCACATTCGTACACTGCTGTTGACCTTCTTCTATCGTCAGATGCCAGAA

PANAN_NN08200 GTCGACGGCTCCCACATTCGTACACTGCTGTTGACCTTCTTCTATCGTCAGATGCCAGAG

PANAN_ARC311 GTCGACGGCTCCCACATTCGTACACTGCTGTTGACCTTCTTCTATCGTCAGATGCCAGAG

PANAN_RSA47 GTCGACGGCTCCCACATTCGTACACTGCTGCTGACCTTCTTCTATCGTCAGATGCCAGAG

PANAN_SGAir0210 GTCGACGGCTCCCACATTCGTACACTGCTGTTGACCTTCTTCTATCGTCAGATGCCAGAG

MIXCA_DSM22759{T} GTCGACGGCTCGCACATTCGTACGCTGCTGTTGACCTTCTTCTATCGTCAGATGCCAGAA

MIXGA_DSM22758{T} GTCGACGGCTCGCACATTCGTACGCTGCTGTTGACCTTCTTCTATCGTCAGATGCCGGAA

MIXAL_LTYR-11Z{T} GTCGACGGCTCGCACATTCGTACGCTGCTGTTGACCTTCTTCTATCGTCAGATGCCGGAA

MIXTH_QC88-366{T} GTCGACGGCTCGCACATTCGTACCCTGCTGTTGACCTTCTTCTATCGTCAGATGCCGGAA

PANRO_LMG26273{T} GTCGATGGCTCGCACATCCGTACGCTGCTGTTGACCTTCTTCTACCGTCAGATGCCGGAA

PANRW_LMG26275{T} GTCGATGGTTCGCACATTCGTACGCTGCTGTTGACCTTCTTCTATCGTCAGATGCCGGAA

PANCY_LMG2657{T} GTCGACGGCTCGCACATTCGTACCCTGCTGTTGACCTTCTTCTATCGTCAGATGCCGGAA

PANEU_LMG5346{T} GTCGATGGCTCACATATCCGTACGCTGCTGCTGACCTTCTTCTACCGTCAGATGCCGGAA

PANWA_LMG26277{T} GTCGATGGCTCGCACATCCGTACGCTGCTGCTGACCTTCTTCTATCGTCAGATGCCGGAA

PANDI_CCUG25232{T} GTCGATGGTTCGCACATCCGTACGCTGCTGCTGACCTTCTTCTATCGCCAGATGCCGGAA

PANSE_LMG5345{T} GTCGACGGCTCGCACATCCGTACGCTGCTGTTGACCTTCTTCTATCGTCAGATGCCGGAA

PANBR_LMG5343{T} GTCGATGGTTCGCACATCCGTACGCTGCTGCTGACTTTCTTCTACCGTCAGATGCCGGAA

PANCO_LMG24534{T} GTCGATGGTTCGCACATCCGTACGCTGCTGCTGACTTTCTTCTACCGTCAGATGCCGGAA

PANAN_LMG2558{T} GTTGATGGTTCACACATCCGTACCCTGCTGCTGACCTTCTTCTATCGTCAGATGCCGGAA

PANDE_LMG24200{T} GTCGATGGTTCACACATCCGTACCCTGCTGCTGACCTTCTTCTATCGTCAGATGCCGGAG

PANVA_LMG24199{T} GTCGATGGTTCACACATCCGTACCCTGCTGCTGACCTTCTTCTATCGTCAGATGCCTGAA

PANEU_LMG24197{T} GTCGATGGTTCCCACATCCGTACCCTGCTGCTGACCTTCTTCTATCGTCAGATGCCTGAA

PANAG_DSM3493{T} GTCGATGGTTCGCACATCCGTACCCTGCTGCTGACCTTCTTCTATCGTCAGATGCCTGAA

PANAG_CFBP13505 GTCGATGGTTCGCACATCCGTACCCTGCTGCTGACCTTCTTCTATCGTCAGATGCCTGAA

TATCI_DSM13699{T} GTCGATGGTTCACACATCCGTACGCTGCTGCTGACCTTCTTCTATCGTCAGATGCCGGAA

TATMO_LMG23360{T} GTCGATGGTTCACACATCCGTACCCTGTTATTGACCTTCTTCTACCGTCAGATGCCGGAG

TATPT_ATCC33301{T} GTCGATGGTTCGCATATCCGTACGCTGCTGCTGACTTTCTTCTATCGTCAGATGCCTGAA

TATSA_NML06-3099{T} GTCGATGGCTCGCACATTCGTACGCTGCTGCTGACCTTCTTCTACCGCCAGATGCCGGAA

** ** ** ** ** ** ** ** *** * **** ** ***** ** ******** **

PANST_CCUG26359{T} ATTATTGAGCGTGGTCACGTCTACATTGCTCAGCCGCCGCTGTATAAAGTGAAAAAAGGC

PANST_LMG2632{PT} ATTATTGAGCGTGGTCACGTCTACATTGCTCAGCCGCCGCTGTATAAAGTGAAAAAAGGC

PANAL_LMG24248{T} ATTATCGAGCGTGGCCACGTTTATATCGCGCAGCCGCCGCTGTACAAGGTGAAAAAAGGC

PANAN_LMG2665{T} ATTATCGAGCGTGGTCACGTGTATATCGCGCAGCCGCCGCTGTATAAGGTGAAAAAAGGT

PANAN_97-1 ATTATCGAGCGTGGTCACGTGTATATCGCGCAGCCGCCGCTGTATAAGGTGAAAAAAGGT

PANAN_LMG5342 ATTATCGAGCGTGGTCACGTGTATATCGCGCAGCCGCCGCTGTATAAGGTGAAAAAAGGT

PANAN_LMG20103 ATTATCGAGCGTGGTCACGTGTATATCGCGCAGCCGCCGCTGTATAAGGTGAAAAAAGGT

PANAN_NN08200 ATTATCGAGCGTGGTCACGTGTATATCGCGCAGCCGCCGCTGTATAAGGTGAAAAAAGGT

PANAN_ARC311 ATTATCGAGCGTGGTCACGTGTATATCGCGCAGCCGCCGCTGTATAAGGTGAAAAAAGGT

PANAN_RSA47 ATTATCGAGCGTGGTCACGTTTATATCGCGCAGCCGCCGCTGTATAAGGTGAAAAAAGGT

PANAN_SGAir0210 ATTATCGAGCGTGGTCACGTGTATATCGCGCAGCCGCCGCTGTATAAGGTGAAAAAAGGT

MIXCA_DSM22759{T} ATCATTGAGCGCGGCCACGTCTATATCGCGCAGCCGCCGCTCTACAAGGTGAAAAAAGGC

MIXGA_DSM22758{T} ATCATCGAACGCGGCCACGTCTATATTGCGCAGCCGCCGCTCTACAAGGTGAAAAAAGGC

MIXAL_LTYR-11Z{T} ATCATCGAGCGCGGCCACGTTTATATTGCGCAGCCGCCGCTGTACAAGGTGAAAAAAGGC

MIXTH_QC88-366{T} ATCATCGAGCGCGGCCACGTTTATATTGCGCAGCCGCCGCTGTACAAGGTGAAAAAAGGC

PANRO_LMG26273{T} ATTGTGGAACGTGGCCATGTCTATATTGCTCAGCCGCCTCTGTATAAAGTGAAGAAAGGC

PANRW_LMG26275{T} ATCGTCGAACGTGGTCATGTTTATATTGCTCAGCCGCCACTGTATAAAGTGAAGAAAGGC

PANCY_LMG2657{T} ATCGTTGAACGTGGCCATGTCTACATTGCTCAGCCGCCGCTGTATAAAGTGAAGAAGGGC

PANEU_LMG5346{T} ATCATTGAGCGCGGCCACGTTTACATTGCGCAGCCGCCGCTGTTTAAAGTGAAAAAAGGC

PANWA_LMG26277{T} ATCATCGAGCGCGGTCATGTCTATATTGCTCAGCCGCCGCTGTACAAAGTGAAGAAAGGC

PANDI_CCUG25232{T} ATCATCGAGCGTGGCCACGTCTACATCGCGCAGCCACCGCTGTACAAGGTGAAGAAGGGC

PANSE_LMG5345{T} ATCATCGAGCGCGGCCACGTCTATATCGCGCAGCCGCCGCTTTACAAGGTGAAGAAAGGC

PANBR_LMG5343{T} ATCATCGAGCGCGGCCACGTTTATATCGCGCAGCCGCCGCTGTATAAAGTGAAGAAAGGC

PANCO_LMG24534{T} ATTATTGAGCGTGGCCATGTATATATCGCTCAGCCGCCACTGTATAAAGTGAAAAAAGGC

PANAN_LMG2558{T} ATCATTGAGCGCGGCCACGTTTATATTGCCCAGCCGCCGCTCTACAAAGTGAAGAAAGGC

PANDE_LMG24200{T} ATCATTGAGCGTGGCCACGTCTATATCGCTCAGCCGCCGCTCTACAAAGTGAAGAAAGGC

PANVA_LMG24199{T} ATCATTGAGCGCGGCCACGTCTATATCGCTCAGCCGCCGCTCTACAAAGTGAAGAAAGGC

PANEU_LMG24197{T} ATCATTGAACGCGGCCATGTTTATATCGCCCAGCCGCCACTCTACAAAGTGAAGAAAGGT

PANAG_DSM3493{T} ATCATTGAGCGCGGCCACGTTTATATCGCCCAGCCGCCACTCTATAAAGTGAAGAAAGGC

PANAG_CFBP13505 ATCATTGAGCGCGGCCACGTTTATATCGCCCAGCCGCCACTCTATAAAGTGAAGAAAGGC

TATCI_DSM13699{T} ATTATTGAGCGTGGTCATGTGTTTATTGCTCAGCCGCCACTGTATAAAGTGAAGAAAGGC

TATMO_LMG23360{T} ATTATTGAGCGTGGTCATGTGTTTATTGCTCAGCCGCCATTGTACAAAGTGAAGAAAGGC

TATPT_ATCC33301{T} ATCATTGAACGCGGCCATGTGTTTATCGCACAGCCACCGCTGTACAAAGTGAAAAAAGGT

TATSA_NML06-3099{T} ATTATCGAACGCGGTCACGTGTTTATTGCCCAGCCGCCACTGTATAAAGTGAAAAAAGGT

** * ** ** ** ** ** * ** ** ***** ** * * ** ***** ** **

PANST_CCUG26359{T} AAGCAGGAACAGTATATTAAAGACGATGAAGCGATGGATCAGTATCAGATCGCTATCGCC

PANST_LMG2632{PT} AAGCAGGAACAGTATATTAAAGACGATGAGGCGATGGATCAGTATCAGATCGCTATCGCC

PANAL_LMG24248{T} AAGCAGGAGCAGTACATCAAGGATGATGAGGCAATGCTTCAGTACCAGATTGCTATTGCC

PANAN_LMG2665{T} AAGCAGGAGCAATACATCAAGGATGACGAAGCCATGCTGCAGTACCAGATTGCTATCGCC

PANAN_97-1 AAGCAGGAGCAATACATCAAGGATGACGAGGCCATGCTGCAGTACCAGATTGCTATCGCC

PANAN_LMG5342 AAGCAGGAGCAATACATCAAGGATGACGAGGCCATGCTGCAGTACCAGATTGCTATCGCC

PANAN_LMG20103 AAGCAGGAGCAATACATCAAGGATGACGAGGCCATGCTGCAGTACCAGATTGCTATCGCC

PANAN_NN08200 AAGCAGGAGCAATACATCAAGGATGACGAAGCCATGCTGCAGTACCAGATTGCTATCGCC

PANAN_ARC311 AAGCAGGAGCAATACATCAAGGATGACGAGGCCATGCTGCAGTACCAGATTGCTATCGCC

PANAN_RSA47 AAGCAGGAGCAATACATCAAGGATGACGAAGCCATGCTGCAGTACCAGATTGCTATCGCC

PANAN_SGAir0210 AAGCAGGAGCAATACATCAAGGATGACGAAGCCATGCTGCAGTACCAGATTGCTATCGCC

MIXCA_DSM22759{T} AAGCAGGAGCAGTACATCAAGGATGATGAGGCGATGGATCAGTATCAGATCGCCATTGCG

MIXGA_DSM22758{T} AAGCAGGAGCAGTACATCAAGGATGACGAGGCGATGGATCAATATCAGATCGCCATCGCG

MIXAL_LTYR-11Z{T} AAGCAGGAGCAGTACATCAAAGATGATGAGGCGATGGATCAGTATCAGATCGCCATTGCG

MIXTH_QC88-366{T} AAGCAGGAGCAGTACATCAAAGATGATGAGGCGATGGATCAGTATCAGATCGCCATTGCG

PANRO_LMG26273{T} AAGCAAGAACAGTACATTAAAGATGACGAAGCGATGGATCAGTATCAGATCGCTATCGCA

PANRW_LMG26275{T} AAGCAAGAACAGTACATTAAAGATGACGAAGCGATGGATCAGTACCAGATCGCTATCGCG

PANCY_LMG2657{T} AAGCAAGAGCAGTACATCAAAGACGACGAAGCGATGGATCAGTATCAAATCGCGATCGCT

PANEU_LMG5346{T} AAGCAGGAACAGTATATTAAAGACACCGACGCGATGGATCAGTATCAGATCGCCATCGCG

PANWA_LMG26277{T} AAGCAAGAGCAGTACATCAAAGATGATGAGGCGATGGATCAGTACAAAATTTCCATCGCC

PANDI_CCUG25232{T} AAGCAGGAGCAGTACATTAAAGATGACGACGCGATGGACCAGTATCAGATTGCCATCGCG

PANSE_LMG5345{T} AAGCAGGAGCAGTACATCAAAGACGATGAGGCGATGGATCAGTATCAGATCGCCATCGCG

PANBR_LMG5343{T} AAGCAGGAGCAGTACATCAAAGATGACGACGCGATGGATCAATACCAGATCGCTATCGCG

PANCO_LMG24534{T} AAGCAGGAGCAGTACATCAAAGATGACGACGCGATGGATCAATACCAGATCGCTATCGCA

PANAN_LMG2558{T} AAGCAGGAGCAGTACATCAAAGATGACGAGGCGATGGATCAGTATCAGATCGCCATCGCG

PANDE_LMG24200{T} AAGCAGGAGCAGTACATCAAAGATGATGAGGCGATGGATCAGTATCAGATCGCTATCGCC

PANVA_LMG24199{T} AAGCAGGAGCAGTACATCAAAGATGACGAGGCGATGGACCAATACCAGATCGCTATCGCC

PANEU_LMG24197{T} AAGCAGGAGCAGTACATCAAAGATGACGAGGCGATGGATCAATACCAGATCGCTATCGCG

PANAG_DSM3493{T} AAGCAGGAGCAGTACATCAAAGATGATGAAGCGATGGATCAATACCAGATCGCTATCGCG

PANAG_CFBP13505 AAGCAGGAGCAGTACATCAAAGATGATGAAGCGATGGATCAATACCAGATCGCTATCGCC

TATCI_DSM13699{T} AAACAGGAACAGTACATTAAAGATGATGATGCGATGGATCAATACCAGATAGCGATTGCG

TATMO_LMG23360{T} AAACAGGAACAGTACATCAAAGATGATGATGCGATGGATCAATACCAGATAGCGATTGCG

TATPT_ATCC33301{T} AAGCAGGAACAGTACATCAAAGATGATGATGCGATGGACCAGTACCAGATAGCGATTGCG

TATSA_NML06-3099{T} AAGCAGGAACAGTACATCAAAGACGATGATGCCATGGACCAGTACCAGATCGCTATCGCT

** ** ** ** ** ** ** ** ** ** *** ** ** * ** * ** **

PANST_CCUG26359{T} CTCGACGGAACGACGCTGCACACCAATGCCAGCGCACCGGCATTAGGTGGTGAGCCGCTT

PANST_LMG2632{PT} CTCGACGGAACGACGCTGCACACCAATGCCAGCGCACCGGCATTAGGTGGTGAGCCGCTT

PANAL_LMG24248{T} CTTGACGGCACTTCGCTGCACACCACCGCCAGCGCACCGGCGTTAGGCGGCGAACCACTG

PANAN_LMG2665{T} CTTGACGGTACCTCGCTGCATACCACCGCCAGCGCACCCGCGCTAGGCGGCGAACCGCTG

PANAN_97-1 CTTGACGGTACCTCGCTGCATACCACCGCCAGCGCACCCGCGCTAGGCGGCGAACCGCTG

PANAN_LMG5342 CTTGACGGTACCTCGCTGCATACCACCGCCAGCGCACCCGCGCTAGGCGGCGAACCGCTG

PANAN_LMG20103 CTTGACGGTACCTCGCTGCATACCACCGCCAGCGCACCCGCGCTAGGCGGCGAACCGCTG

PANAN_NN08200 CTTGACGGTACCTCGCTGCATACCACCGCCAGCGCACCCGCGCTAGGCGGCGAACCGCTG

PANAN_ARC311 CTTGACGGTACCTCGCTGCATACTACCGCCAGCGCACCCGCGCTAGGCGGCGAACCGCTG

PANAN_RSA47 CTTGACGGTACCTCGCTGCATACTACCGCCAGCGCACCCGCGCTAGGCGGCGAACCGCTG

PANAN_SGAir0210 CTTGACGGTACCTCGCTGCATACTACCGCCAGCGCACCCGCGCTAGGCGGCGAACCACTG

MIXCA_DSM22759{T} CTGGACGGCGCGACGCTACACACCAACGCCAGCGCGCCGGCGCTCGGCGGCGAACAGCTG

MIXGA_DSM22758{T} CTGGACGGCGCGACGCTGCACACCAACGCCAGCGCGCCGGCGCTGGGCGGCGAACAGCTG

MIXAL_LTYR-11Z{T} CTGGATGGCGCAACGCTCCATACCAACGCCAGCGCCCCTGCGCTGGGCGGTGAGCCGTTG

MIXTH_QC88-366{T} CTGGATGGCGCAACGCTGCACACCAACGGAGCCGCGCCGGCTCTGGGCGGCGAACCGTTG

PANRO_LMG26273{T} TTGGATGGTGCAACGCTGCACACCAACGCCAGCGCACCGGCGCTTGGCGGCCAGCCGCTG

PANRW_LMG26275{T} CTTGATGGTGCAACGCTGCACACCAACGCCAGTGCTCCGGCGCTGGGTGGCCAGCCGCTG

PANCY_LMG2657{T} CTGGATGGCGCGACCCTGCACACCAACGCCAGCGCCCCGGCACTGGGTGGCGAGCCGCTG

PANEU_LMG5346{T} CTGGACGGCGCTACGCTGCACACCAACGCCAGCGCACCGGCGCTGGGCGGCGAGCCGCTG

PANWA_LMG26277{T} ATGGACGGTGCCACGCTGCACACCAACGCCAGTGCTCCAGCCCTGGGCGGCGAACCGCTG

PANDI_CCUG25232{T} CTGGACGGCGCAACGCTGCACACCAACGCCAGCGCACCGGCGCTGGGCGGCGAGCCGCTG

PANSE_LMG5345{T} CTCGACGGCGCGACGCTGCACACCAACGCCAGCGCACCGGCGCTGGGCGGCGAGCCGCTG

PANBR_LMG5343{T} CTTGATGGTGCCACGCTGCACACTAATGCCAGCGCCCCGGCCCTGGCTGGTGAGCCGCTG

PANCO_LMG24534{T} CTGGATGGTGCCACGCTGCACACCAATGCCAGCGCCCCGGCCCTGGCGGGTGAGCCGCTG

PANAN_LMG2558{T} CTGGATGGTGCCACGCTGCACACCAACGCCAGCGCTCCGGCACTGGGCGGTGAGCCGCTG

PANDE_LMG24200{T} CTGGACGGGGCCACGCTGCACACCAATGCCAGCGCGCCAGCCCTGGGCGGTGAGCCGCTG

PANVA_LMG24199{T} CTTGATGGTGCAACGCTGCACACCAATGCCAGCGCGCCTGCGCTGGGCGGTGAGCCGCTG

PANEU_LMG24197{T} CTTGATGGTGCGACGCTGCACACCAATGCCAGCGCACCCGCGCTGGGCGGTGAGCCGCTG

PANAG_DSM3493{T} CTTGATGGCGCGACGCTGCACACCAATGCCAGCGCGCCGGCGCTGGGCGGTGAGCCGCTG

PANAG_CFBP13505 CTTGATGGTGCGACGCTGCACACCAATGCCAGCGCGCCGGCGCTGGGCGGTGAGCCGCTG

TATCI_DSM13699{T} CTGGATGGTGCGACCCTGCATACCAATGCCAGTGCTCCGGCACTGAGTGGTGCTTCGCTT

TATMO_LMG23360{T} CTGGATGGTGCGACCCTGCATACCAATTCCAGTGCACCGGCATTAAGTGGTGCTTCGCTT

TATPT_ATCC33301{T} CTGGACGGAGCTACCCTGCATACCAATGCCAGCGCACCTGCGCTGAGCGGAGCCTCGCTG

TATSA_NML06-3099{T} CTGGACGGTGCCACTCTGCATACCAACGCGAGTGCGCCAGCTCTGAGCGGTGCATCGCTG

* ** ** * * ** ** ** * ** ** ** * ** *

PANST_CCUG26359{T} GAATCTCTGGTCGCGGACTTTAACAGCACGCAGAAGATGATCAAACGTATGGAGCGCCGT

PANST_LMG2632{PT} GAATCTCTGGTCGCGGACTTTAACAGCACGCAGAAGATGATCAAACGTATGGAGCGCCGT

PANAL_LMG24248{T} GAAACGCTGGTGGCTGAATTCAACAGCACCCAGAAGATGATCAAACGTATGGAGCGCCGT

PANAN_LMG2665{T} GAAACACTGGTCGCTGAATTCAACAGCACTCAGAAGATGATCAAACGCATGGAGCGTCGT

PANAN_97-1 GAAACACTGGTCGCTGAATTCAACAGCACTCAGAAGATGATCAAACGCATGGAGCGTCGT

PANAN_LMG5342 GAAACACTGGTCGCTGAATTCAACAGCACTCAGAAGATGATCAAACGCATGGAGCGTCGT

PANAN_LMG20103 GAAACACTGGTCGCTGAATTCAACAGCACTCAGAAGATGATCAAACGCATGGAGCGTCGT

PANAN_NN08200 GAAACACTGGTCGCTGAATTCAACAGCACTCAGAAGATGATCAAACGCATGGAGCGTCGT

PANAN_ARC311 GAAGCGCTGGTCGCTGAATTCAACAGCACTCAGAAGATGATCAAACGCATGGAGCGTCGT

PANAN_RSA47 GAAACGCTGGTCGCTGAATTCAACAGCACTCAGAAGATGATCAAACGCATGGAGCGTCGT

PANAN_SGAir0210 GAAACGCTGGTCGCTGAATTCAACAGCACTCAGAAGATGATCAAACGCATGGAGCGTCGT

MIXCA_DSM22759{T} GAAAACCTGGTCTCCGACTACAACAGCACGCAGCGCATGATCAAGCGTATGGAGCGCCGC

MIXGA_DSM22758{T} GAAAAAATGGTCTCCGACTACAACAGCACGCAGCGCATGATCAAGCGCATGGAGCGCCGC

MIXAL_LTYR-11Z{T} GAAAATCTGGTTTCCGAGTACAACAGCACGCAGCGCATGATCAAGCGTATGGAACGTCGT

MIXTH_QC88-366{T} GAAAGCCTGGTTTCCGAGTACAACAGCACGCAGCGCATGATCAAGCGTATGGAACGTCGC

PANRO_LMG26273{T} GAAAATCTGGTGGGCGATTTCAACAGCACCCAGCGCATGATCAAGCGTATGGAACGTCGT

PANRW_LMG26275{T} GAAAATCTGGTGGGTGATTTCAACAGCACCCAGCGCATGATCAAGCGTATGGAACGCCGT

PANCY_LMG2657{T} GAAAATCTGGTGGCGAGCTTCAACAGCACGCAACGCATGATCAAACGTATGGAGCGCCGC

PANEU_LMG5346{T} GAGAGCCTGGTCGCGGAGTACAACAGTACGCAGAAGATGATCAAACGCATGGAGCGTCGC

PANWA_LMG26277{T} GAGAATCTGGTGGCTGAGTACAACAGCACGCAGAAGATGATCAAGCGTATGGAGCGCCGC

PANDI_CCUG25232{T} GAAAGCCTGGTGGCGGAATACAACAGCACCCAGAAGATGATCAAGCGTATGGAGCGCCGC

PANSE_LMG5345{T} GAAAATCTGGTCGCGGAGTTCAACAGCACGCAGAAGATGATCAAGCGTATGGAGCGCCGC

PANBR_LMG5343{T} GAGACGCTGGTCTCTGACTTCAACAGCACCCAGAAGATGATCAAGCGTATGGAGCGCCGT

PANCO_LMG24534{T} GAGACGCTGGTCTCTGACTTCAACAGCACCCAGAAGATGATCAAGCGTATGGAGCGCCGT

PANAN_LMG2558{T} GAGACGCTGGTCTCTGACTTCAACAGCACGCAGAAGATGATCAAGCGTATGGAGCGCCGT

PANDE_LMG24200{T} GAGTCACTGGTGTCTGAATTCAACAGCACCCAGAAGATGATCAAGCGGATGGAACGCCGT

PANVA_LMG24199{T} GAGACGCTGGTCTCTGACTTCAACAGCACCCAGAAGATGATCAAGCGTATGGAACGCCGT

PANEU_LMG24197{T} GAGACACTGGTCTCTGACTTCAACAGTACCCAGAAGATGATCAAGCGTATGGAACGCCGT

PANAG_DSM3493{T} GAGACGCTGGTCACTGACTTCAACAGCACCCAGAAGATGATCAAGCGTATGGAGCGCCGT

PANAG_CFBP13505 GAGACGCTGGTCACTGACTTCAACAGCACCCAGAAGATGATCAAGCGTATGGAGCGCCGT

TATCI_DSM13699{T} GAAGAGCTGGTGGCTATGTTTAATAACACCGAAAAAATGATCAAGCGGTTGTCCCGTCGT

TATMO_LMG23360{T} GAAGAGTTGGTAGCGATGTTCAATTACACCGAAAAGATGATTAAGCGGATGACCCGCCGT

TATPT_ATCC33301{T} GAAAATCTGGTATCAATGTACAACAGTACAGAGAAGATGATTAAGCGCATGACCCGTCGC

TATSA_NML06-3099{T} GAAAATCTGGTCTCTATGTACAACAGCACTGAGAAAATGATTAAGCGTATGACCCGCCGT

** **** * ** ** * ***** ** ** ** ** **

PANST_CCUG26359{T} TTTCCACTGGCGATGTTACGTGCGCTGGTCTATCACGACACGCTGAGCGATCTGAGTAAT

PANST_LMG2632{PT} TTTCCACTGGCGATGTTACGTGCGCTGGTCTATCACGACACGCTGAGCGATCTGAGTAAT

PANAL_LMG24248{T} TATCCGGTCGCCATGCTGCGTGCGCTGATTTATCACGATACCCTGAGCGATATCAGCAAC

PANAN_LMG2665{T} TATCCGATGGCCATGCTGCGTGCGCTGATTTATCACGATACACTGAGCGATATCAGCAAC

PANAN_97-1 TATCCGATGGCCATGCTGCGTGCGCTGATTTATCACGACACACTGAGCGATATCAGCAAC

PANAN_LMG5342 TATCCGATGGCCATGCTGCGTGCGCTGATTTATCACGACACACTGAGCGATATCAGCAAC

PANAN_LMG20103 TATCCGATGGCCATGCTGCGTGCGCTGATTTATCACGACACACTGAGCGATATCAGCAAC

PANAN_NN08200 TATCCGATGGCCATGCTGCGTGCGCTGATTTATCACGATACACTGAGCGATATCAGCAAC

PANAN_ARC311 TATCCGATGGCCATGCTGCGTGCGCTGATTTATCACGACACACTGAGTGATATCAGCAAC

PANAN_RSA47 TATCCGATGGCCATGCTGCGTGCGCTGATTTATCACGACACACTGAGCGATATCAGCAAC

PANAN_SGAir0210 TATCCGATGGCCATGCTGCGTGCGCTGATTTATCACGACACACTGAGCGATATCAGCAAC

MIXCA_DSM22759{T} TTCCCGCTCGCGCTGCTTAACGCGCTGGTTTACCATCCGACGCTGGAAAGCCTGGACAAC

MIXGA_DSM22758{T} TTCCCGATCGCGTTGCTTAACGCGCTGGTTTACCATCCGACGCTGGAAAACCTGGATAAC

MIXAL_LTYR-11Z{T} TTCCCGATGGCGCTGCTTAAATCGCTGGTTTACCATCCGACGCTGGAAAGCCTGGATAAC

MIXTH_QC88-366{T} TTCCCGATGGCCCTGCTTAAAGCGCTGGTTTACCATCCGACGCTGGAAAGCCTGGATAAC

PANRO_LMG26273{T} TTCCCAACGGCAATGCTGCGTGGCCTGATTTATCATCCAACCCTGAGCGACCTCGCTGAT

PANRW_LMG26275{T} TTCCCAACGGCAATGCTGCGTGGCCTGATCTATCATCCAACCCTGAGCGATCTCAGCAAT

PANCY_LMG2657{T} TACCCGCTCGCGCTGCTGCGTGGCCTGATTTACCACCCAACCCTGAGCGATCTCAGCGAT

PANEU_LMG5346{T} TTCCCGGCGGCGCTGCTGAACGCGCTGGTTTACCATCCAACGCTGGCGGGGCTGGAAGAT

PANWA_LMG26277{T} TATCCGGTTGCGCTGCTGCGCGCGCTGGTTTATCGCCCGACGCTGAGCGAGCTTAGCAAC

PANDI_CCUG25232{T} TTCCCGTCTGCGCTGCTGCGCGCGCTGGTTTATCATCCAACCCTGAGCGATCTCAGCGAT

PANSE_LMG5345{T} TATCCGGTCGCGCTGCTGCGCGCGCTGGTTTATCACCCAACGCTGAGCGATCTGAGCAAC

PANBR_LMG5343{T} TATCCGGTTGCC-TGCTGCGCGCGCTGATTTATCACGACACGCTGAGCGATCTGAGCAAT

PANCO_LMG24534{T} TATCCGCTGGCCCTGCTGCGCGCGCTGATTTATCACGACACGCTGAGCGATCTGAGCGAT

PANAN_LMG2558{T} TATCCGATGGCCATGCTGCGCGCGCTGATTTATCACGACACGCTGAGCGATCTGACCAAT

PANDE_LMG24200{T} TATCCGGTTGCCATGCTGCGTGCGCTGATCTATCACGACACGCTGAGCGATCTGGGCAAC

PANVA_LMG24199{T} TATCCGATGGCCATGCTGCGTGCGATGATTTATCACGACACGCTGAGCGACCTGAGCAAC

PANEU_LMG24197{T} TATCCGATGGCGATGCTGCGTGCGATGATTTATCACGACACGCTGAGCGATCTGAGCAAC

PANAG_DSM3493{T} TATCCGATGGCCATGCTGCGTGCGATGATTTATCACGACACGCTGAGCGACCTGACCAGC

PANAG_CFBP13505 TATCCGATGGCCATGCTGCGTGCGATGATTTATCACGACACGCTGAGCGACCTGAGCAAT

TATCI_DSM13699{T} TATCCTGCAATGCTGCTGAAAAGGCTGGTCTACCGTCCGACCCTGAGCGATCTTAGCAGT

TATMO_LMG23360{T} TATCCTGCAGCGATGCTGAAAAGACTGGTGTATCGTCCGACCCTGAGCGATCTCACCGAC

TATPT_ATCC33301{T} TATCCGGCGGCATTACTGAGAGGGCTGGTGTATCACCCGACGCTGAGTGATTTATCTGAC

TATSA_NML06-3099{T} TATCCGTCAGCACTGCTGCGGGGCTTAGTGTATCACTCGACCCTGAGCGACTTGTCAGAG

* ** * * * * ** * ** *** *

PANST_CCUG26359{T} GAAGCGGCCGTTAACGCCTGGATTAACGGACTGGTGAGCTATCTGACTGAACGTGAAGCG

PANST_LMG2632{PT} GAAGCGGCCGTTAACGCCTGGATTAACGGACTGGTGAGCTATCTGACTGAACGTGAAGCG

PANAL_LMG24248{T} GAAGCGCAAGTGACCCTGTGGATCAACGGCCTGGTTAGCTACCTGACCGAACGCGAAGCG

PANAN_LMG2665{T} GAAGCGCAGGTGACCAGTTGGATTAACGGTCTGGTTAGCTACCTGACAGCACGCGAGGCG

PANAN_97-1 GAAGCGCAGGTGACCAGTTGGATCAACGGTCTGGTTAGCTACCTGACAGCACGCGAGGCG

PANAN_LMG5342 GAAGCGCAGGTGACCAGTTGGATCAACGGTCTGGTTAGCTACCTGACAGCACGCGAGGCG

PANAN_LMG20103 GAAGCGCAGGTGACCAGTTGGATCAACGGTCTGGTTAGCTACCTGACAGCACGCGAGGCG

PANAN_NN08200 GAAGCGCAAGTGACCAGTTGGATCAACGGTCTGGTTAGCTACCTGACAGCACGCGAGGCG

PANAN_ARC311 GAAGCGCAGGTGACCCGTTGGATCAACGGTCTGGTTAGCTACCTGACAGCACGCGAGGCG

PANAN_RSA47 GAAGCGCAGGTGACCCGTTGGATCAACGGTCTGGTTAGCTACCTGACAGCACGCGAGGCG

PANAN_SGAir0210 GAAGCGCAGGTGACCCGTTGGATCAACGGTCTGGTTAGCTACCTGACAGCACGCGAGGCG

MIXCA_DSM22759{T} CAGCAGACGGTGACGACCTGGATCGAGACGCTGGTTACGCTGCTGAACGAAAGAGAAACG

MIXGA_DSM22758{T} CAGCAGGCAGTCAGCAGCTGGATCGATGCGCTGGTTACGCTGCTGAATGAAAGGGAAACC

MIXAL_LTYR-11Z{T} CAACAGACGGTTACCGAGTGGATCGATACGCTGGTTACCCGGCTGAATGAAACAGAGACG

MIXTH_QC88-366{T} CAACAGACGGTCACCGACTGGATCGATACGCTGGTTACCTTGCTGAATGAAAAAGAGACC

PANRO_LMG26273{T} GAAGGCGTGGTGAAGGGCTGGATTGAGGGCTTAGTCGCTTACCTGACCGAGCGCGAAGCG

PANRW_LMG26275{T} GAAGCCGCGGTTAAAGGCTGGATTGAAGGTTTAGTTGCATACCTGACCGAGCGTGAAGCG

PANCY_LMG2657{T} GAGGCCGCAGTTAAAGGTTGGCTGGATGGGCTGGTGGCGTACCTGACCGAGCGCGAAGCG

PANEU_LMG5346{T} ACCGCTGCCGTCAGCAGCTGGCTGGAGACGCTGGTGGCGTATCTCAACGAGCGTGAAGCA

PANWA_LMG26277{T} GAAGCCGCAGTGAAAACCTGGCTGGATGAGCTGGTGGCTTACCTCAACGAGCGTGAAGCG

PANDI_CCUG25232{T} CAGACAGCAGTGACCGCATGGCTGGAGAGCCTGGTGGCTTACCTGAACGAGCGCGAAGCG

PANSE_LMG5345{T} GAGGCTGACGTTCAGGCCTGGCTGGACAAGCTGGTGGCCTTCCTGGGCGAGCGCGAGACG

PANBR_LMG5343{T} GAAGCAGAAGTGACCCGCTGGCTGAACAGCCTGGTGAGCTATCTCACTGAGCGCGAAGCG

PANCO_LMG24534{T} GAAGCGGAAGTGACCCGCTGGCTGAACAGTCTGGTGAGCTATCTCACCGAGCGCGAAGCG

PANAN_LMG2558{T} GAAGCGGAGGTCACGACCTGGCTGAACGGCCTGGTGAGCTATCTGACTGAACGCGAAGCG

PANDE_LMG24200{T} GAAGCGGAAGTCACCACCTGGCTGAACGGTCTGGTGAGCTATCTGACCGAGCGCGAAGCC

PANVA_LMG24199{T} GAAGCAGAGGTCACTACCTGGCTGAACGGCCTGGTGAGCTATCTGACGGAGCGTGAAGCG

PANEU_LMG24197{T} GAAGCAGAGGTCACCACCTGGCTGAACGGTCTGGTGAGCTATCTGACCGAACGCGAAGCG

PANAG_DSM3493{T} GAAGCAGAGGTTACAACCTGGCTGAATGGTCTGGTGAGCTATCTGACGGAGCGCGAAGCG

PANAG_CFBP13505 GAAGCGGAGGTTACAACCTGGCTGAATGGTCTGGTGAGCTATCTGACTGAACGCGAAGCG

TATCI_DSM13699{T} CAGGCTACAGTCCAACAGTGGCTGGACGAGCTGGTGAGTGATTTGACCGAACGTGAAGTG

TATMO_LMG23360{T} CAGGCAACCGTTCAGCAGTGGCTGGATGGACTGGTGAGTGATTTAAACGAACGCGAAGCG

TATPT_ATCC33301{T} CAGGCGGCCGTACAGGCGTGGATGGATGCTCTGGTGGCCCAACTGGAAGAACGCGAACAG

TATSA_NML06-3099{T} CAGTCGGCGGTGCAGCAGTGGTTGGATAAGCTGGTAGCTTCCTTGGCGGAACGCGAGCAG

** *** * * * ** * * **

PANST_CCUG26359{T} CACGGCAGCACCTATGTGGCACACGTACGTGAAAATCGTGAGCTGAATCAGTTTGAGCCT

PANST_LMG2632{PT} CACGGCAGCACCTATGTGGCACACGTACGTGAAAATCGTGAGCTGAATCAGTTTGAGCCT

PANAL_LMG24248{T} CACGGCAGCACCTATCTGGCACAGGTTCGTGAAAACCGTGAGCAGAGCGTCTTTGAGCCA

PANAN_LMG2665{T} CACGGCAGTACCTATCTGGCCCAGGTGCGTGAGAACAGTGAGCAGAATGTCTTTGAGCCG

PANAN_97-1 CACGGCAGTACCTATCTGGCCCAGGTGCGTGAGAACCGTGAGCAGAATGTCTTTGAGCCG

PANAN_LMG5342 CACGGCAGTACCTATCTGGCCCAGGTGCGTGAGAACCGTGAGCAGAATGTCTTTGAGCCG

PANAN_LMG20103 CACGGCAGTACCTATCTGGCCCAGGTGCGTGAGAACCGTGAGCAGAATGTCTTTGAGCCG

PANAN_NN08200 CACGGCAGTACCTATCTGGCCCAGGTGCGTGAGAACCGTGAGCAGAATGTCTTTGAGCCG

PANAN_ARC311 CACGGCAGTACCTATCTGGCCCAGGTGCGTGAGAACCGTGAGCAGAATGTCTTTGAGCCG

PANAN_RSA47 CACGGCAGTACCTATCTGGCCCAGGTGCGTGAGAACCGTGAGCAGAATGTCTTTGAGCCG

PANAN_SGAir0210 CACGGCAGTACCTATCTGGCCCAGGTGCGTGAGAACCGTGAGCAGAATGTCTTTGAGCCG

MIXCA_DSM22759{T} CACGGCAGTACCTATGTCGCCAGCGTGCGTGAAAACCGCGAGCTGAACATCTTCGAACCG

MIXGA_DSM22758{T} CACGGCAGCACCTATGTCGCCAGCGTGCGTGAAAACCGCGAGCTGAATATTTTCGAACCG

MIXAL_LTYR-11Z{T} CACGGCAGCACCTATGTTGCCAGCGTGCGCGAAAACCGCGAGCTGAATGTTTTTGAACCG

MIXTH_QC88-366{T} CACGGCAGTACCTACGTTGCCAGCGTGCGTGAAAATCGTGAGCTGAATATTTTTGAGCCG

PANRO_LMG26273{T} CACGGCAGCAGCTACATGGCGCACGTGCGCACTAACAGCGAACTGAACCTGTTCGAACCA

PANRW_LMG26275{T} CACGGCAGCAGCTATATGGCGCACGTGCGCACCAATAGCGAACTGAACGTGTTCGAACCG

PANCY_LMG2657{T} CACGGCAGCACCTACGTCGCGCAGGTGCGCGCTAACCGTGAACAGAACCTGTTCGAGCCG

PANEU_LMG5346{T} CACGGCAGCACCTATGTGGCACAGGTACGCGAAAACCGTGAGCTGCAGCAGGCTGAGCCG

PANWA_LMG26277{T} CACGGCAGCAGCTATATGGCGCAGGTGCGTGAAAACCGTGAGCAGCAGCTGTTCGAGCCG

PANDI_CCUG25232{T} CATGGCAGCAGCTATGTGGCACAGGTGCGGGAAAACCGCGAACAGCAGCGCTTTGAGCCG

PANSE_LMG5345{T} CACGGCAGCAGCTATGTTGCTCAGGTACGCGAAAACCGCGAGCAGAACATCTTCGAACCG

PANBR_LMG5343{T} CACGGTAGTACCTATCTGGCGCAGGTGCGTGAAAACCGCGAGCTGAACCTGTTCGAACCG

PANCO_LMG24534{T} CACGGCAGCACCTACCTGGCGCAGGTGCGTGAAAACCGCGAGCTGAACCTGTTCGAACCG

PANAN_LMG2558{T} CACGGCAGTACTTATCTGGCCCAGGTTCGCGAAAACCGCGAGCTGAACCTCTTCGAGCCG

PANDE_LMG24200{T} CACGGCAGTACCTGGCTGGCCCAGGTGCGTGAAAACCGCGAACAGAACCTGTTCGAGCCG

PANVA_LMG24199{T} CACGGCAGTACTTATCTGGCACAGGTGCGTGAAAACCGCGAACTGAACCTGTTCGAGCCG

PANEU_LMG24197{T} CACGGCAGCACCTATCTGGCACAGGTGCGTGAAAACCGCGAAACGAACCTGTTCGAGCCG

PANAG_DSM3493{T} CACGGCAGCACCTATCTGGCACAGGTGCGTGAAAACCGTGAATCAAACCTGTTCGAGCCG

PANAG_CFBP13505 CATGGCAGCACCTATCTGGCACAGGTGCGTGAAAACCGCGAATCAAACCTGTTCGAGCCG

TATCI_DSM13699{T} CATGGCAGTAGCTACTCCGGCTTCGTGCGGGAAAATCGGGAACACCAGCGTTTTGAGCCA

TATMO_LMG23360{T} CATGGCAGCAGCTATTCCGGTTACGTGCGGGAAAATCGTGAGCATCAGCGTTTTGAGCCG

TATPT_ATCC33301{T} CACGGTAGCAGCTATACCGGTATTGTCCGTGAAGACCGTGAACATCAGCGTTTTGAGCCG

TATSA_NML06-3099{T} CACGGCAGTGCCTATATCGGTATTGTCCGTGAAGACCGCGAGCATCAACGTTTTGAGCCG

** ** ** * * ** ** * * ** ** **

PANST_CCUG26359{T} GTTTTACGCGTACGGACCCATGGCGTGGACACGGATTATCCTCTGGACAGCGAGTTTATT

PANST_LMG2632{PT} GTTTTACGCGTACGGACCCATGGCGTGGACACGGATTATCCTCTGGACAGCGAGTTTATT

PANAL_LMG24248{T} GTTTTACGCGTCCGTACGCATGGCGTGGATACCGACTATCCTCTGGATGCCGAGTTCCTG

PANAN_LMG2665{T} GTTTTACGCGTTCGTACACATGGAGTGGACACGGATTATCCTCTGGATGCCGAGTTCCTG

PANAN_97-1 GTTTTACGCGTTCGTACACATGGAGTGGACACGGATTATCCTCTGGATGCCGAGTTCCTG

PANAN_LMG5342 GTTTTACGCGTTCGTACACATGGAGTGGACACGGATTATCCTCTGGATGCCGAGTTCCTG

PANAN_LMG20103 GTTTTACGCGTTCGTACACATGGAGTGGACACGGATTATCCTCTGGATGCCGAGTTCCTG

PANAN_NN08200 GTTTTACGCGTTCGTACACATGGAGTGGACACGGATTATCCTCTGGATGCCGAGTTCCTG

PANAN_ARC311 GTTTTACGCGTTCGTACACATGGCGTGGACACGGATTATGCTCTGGATGCCGAGTTCCTG

PANAN_RSA47 GTTTTACGCGTTCGTACACATGGCGTGGACACGGATTATCCTCTGGATGCCGAGTTCCTG

PANAN_SGAir0210 GTTTTACGCGTTCGTACACATGGCGTGGACACGGATTATCCTCTGGATGCCGAGTTCCTG

MIXCA_DSM22759{T} GTGCTGCGCGTGCGCACGCACGGCGTGGATACCGATTATCCGCTGGACAGCGAGTTTATC

MIXGA_DSM22758{T} GTGCTGCGCGTACGCACGCACGGCGTGGATACCGATTATCCGCTGGACAGTGAGTTTATT

MIXAL_LTYR-11Z{T} GTACTGCGCGTGCGTACGCACGGCGTGGATACTGACTATCCGCTGGATAGCGAATTTATT

MIXTH_QC88-366{T} GTACTGCGCGTACGTACGCACGGCGTGGATACCGACTATCTGCTGGATAGCGAATTTATT

PANRO_LMG26273{T} GTTCTGCGTGTGCGTACGCACGGTGTCGATACCGACTATCCGATGGACAGCGAGTTCATC

PANRW_LMG26275{T} GTATTACGTGTGCGTACGCACGGTGTTGATACCGACTATCCAATGGATAACGAGTTCATC

PANCY_LMG2657{T} GTGCTGCGTGTGCGTACCCACGGCGTGGATACCGATTACCCGCTGGACAGCGAATTCATC

PANEU_LMG5346{T} GTACTGCGTGTACGCACCCACGGTGTGGATACCGACTATCCGCTGGATAGCGAGTTTATC

PANWA_LMG26277{T} GTGCTGCGCGTACGCACGCACGGCGTGGATACCGATTATCCGCTGGACAGCGAGTTCATC

PANDI_CCUG25232{T} GTGCTGCGCGTGCGTACTCACGGTGTTGATACCGATTATCCGCTGGACAGCGAGTTCATT

PANSE_LMG5345{T} GTTCTGCGCGTGCGCACGCACGGCGTCGACACCGATTATACGCTGGACGCCGAGTTCATT

PANBR_LMG5343{T} GTGCTGCGCGTCCGTACCCACGGTGTGGATACTGATTATCCGCTGGAAGCCGATTTCATC

PANCO_LMG24534{T} GTGCTGCGTGTCCGTACCCACGGTGTGGATACCGATTATCCGCTGGAAGCCGATTTCATT

PANAN_LMG2558{T} GTTCTGCGCGTTCGCACCCACGGTGTGGATACCGACTACCCGCTGGAAGCGGAGTTCATT

PANDE_LMG24200{T} GTACTGCGCGTGCGTACCCACGGTGTGGATACCGACTATCCGCTGGACGCCGAGTTCATC

PANVA_LMG24199{T} GTGCTGCGCGTCCGTACCCACGGTGTGGATACCGACTATCCGCTGGAAGCTGAGTTTGTT

PANEU_LMG24197{T} GTGCTGCGCGTCCGTACCCACGGTGTGGATACCGACTATCCGCTGGAAGCGGATTTCATT

PANAG_DSM3493{T} GTACTGCGCGTCCGTACCCACGGTGTAGATACTGACTATCCGCTGGATGCTGATTTCATT

PANAG_CFBP13505 GTACTGCGCGTCCGTACCCACGGTGTAGATACTGACTATCCGCTGGATGCTGATTTCATT

TATCI_DSM13699{T} GTACTGCGGATCAAAACTCATGGGGTTGATACTGATTACCCTCTGGATTCTGAATTTATT

TATMO_LMG23360{T} GTCTTACGGGTCAGAACTCATGGGGTGGATACAGACTACCCACTGGATTATGAATTTATT

TATPT_ATCC33301{T} GTACTTCGTATTCGTACACATGGTGTGGATACGGATTATCCTCTGGATTCAGAATTTATT

TATSA_NML06-3099{T} GTACTGCGTATCCGTACCCATGGCGTGGATACTGATTATCCGCTGGACTCCGAGTTTATC

** * ** * ** ** ** ** ** ** ** ** **** ** ** *

PANST_CCUG26359{T} ATGGGGCCGGAATATCGCAAAATTTGCGCCCTGGGTGGCAAACTGCGCGGCCTGCTGGAA

PANST_LMG2632{PT} ATGGGGCCGGAATACCGCAAAATTTGCGCCCTGGGTGGCAAACTGCGCGGCCTGCTGGAA

PANAL_LMG24248{T} CAGGGTGCAGAATACCGCAAGATCTGCACTCTGGGCGGCAAACTGCGCGGTTTGCTGGAA

PANAN_LMG2665{T} CAGGGTGGCGAATACCGCAAGATCTGTGCACTGGGCGGCAAGCTTCGCGGCCTGCTTGAA

PANAN_97-1 CAGGGTGGCGAATACCGCAAGATCTGTGCACTGGGCGGCAAGCTTCGCGGCCTGCTTGAA

PANAN_LMG5342 CAGGGTGGCGAATACCGCAAGATCTGTGCACTGGGCGGCAAGCTTCGCGGCCTGCTTGAA

PANAN_LMG20103 CAGGGTGGCGAATACCGCAAGATCTGTGCACTGGGCGGCAAGCTTCGCGGCCTGCTTGAG

PANAN_NN08200 CAGGGTGGCGAATACCGCAAGATCTGTGCACTGGGCGGCAAGCTTCGCGGCCTGCTTGAG

PANAN_ARC311 CAGGGTGGCGAATACCGCAAGATCTGTGCACTGGGCGGCAAGCTTCGCGGTCTTCTTGAA

PANAN_RSA47 CAGGGTGGCGAATACCGCAAGATCTGTGCACTGGGCGGCAAGCTTCGCGGTCTGCTTGAA

PANAN_SGAir0210 CAGGGTGGCGAATACCGCAAGATCTGTGCACTGGGCGGCAAGCTTCGCGGTCTGCTTGAA

MIXCA_DSM22759{T} CAGGGCCCGGAATACCGCAAAATCTGCGCGCTGGGTGGAAAACTGCGCGGCCTGCTGGAA

MIXGA_DSM22758{T} CAGGGCCCGGAATACCGCAAAATTTGCGCCCTGGGCGACAAACTGCGCGGCCTGCTGGAA

MIXAL_LTYR-11Z{T} CAGGGACCGGAATATCGCAAAATCTGTACGCTGGGCATGAAACTGCGCGGTCTGTTGGAA

MIXTH_QC88-366{T} CAGGGGCCGGAATATCGCAAAATCTGTACGCTGGGCGCTAAGCTGCGCGGTCTGCTGGAA

PANRO_LMG26273{T} GATGGACCGGAATACCGCAAAATCTGTGGTTTGGGCGAGCAGCTGCGCGGCCTGATCGAA

PANRW_LMG26275{T} GATGGACCGGAGTATCGCAAAATCTGTGGTCTGGGCGAGCAACTGCGCGGCCTGATTGAA

PANCY_LMG2657{T} GGCGGTCCGGAATATCGCAAAATCTGCGAGCTCGGCGAGAAGCTGCGTGGTCTGATCGAG

PANEU_LMG5346{T} GGTGGTCCGGAGTATCGCAAAATCTGTGCGCTGGGCGAGCAGCTGCGCGGCCTGATTGAA

PANWA_LMG26277{T} GCGGGTCCGGAATATCGCAAGATCTGCGCGCTGGGCGAGCAGTTACGCGGCCTGATCGAA

PANDI_CCUG25232{T} GACGGCGCGGAATACCGCAAAATCTGTGCACTCGGCGCGCACCTGCGTGGTTTGATTGAA

PANSE_LMG5345{T} CAGGGACCGGAGTACCGTAAAATCAACGCGCTGGGCGAGAAGCTGCGCGGCCTGATTGAG

PANBR_LMG5343{T} CAGGGTCCTGAGTACCGCAAAATCTGTGGCCTCGGCGACAAGCTGCGTGGCCTGCTGGAA

PANCO_LMG24534{T} CAGGGTCCTGAGTACCGCAAAATCTGCGGCCTTGGCGGCAAACTGCGTGGCCTGCTGGAA

PANAN_LMG2558{T} CAGGGGCCGGAGTACCGCAAAATCTGCGATCTTGGCGGCAAACTGCGTGGCCTGCTGGAA

PANDE_LMG24200{T} CAGGGCCCGGAATACCGCAAAATCTGCGCACTGGGCAGCAAGCTGCGCGGTCTGCTGGAA

PANVA_LMG24199{T} CAGGGACCTGAGTACCGCAAAATCTGTGCTCTGGGCGGCAAACTGCGTGGTCTGCTGGAA

PANEU_LMG24197{T} CAGGGGCCTGAGTATCGCAAAATCTGCGCGCTGGGTAGCCAGCTGCGTGGTCTGCTGGAA

PANAG_DSM3493{T} CAGGGTCCTGAGTACCGCAAAATCTGCGCCCTGGGCGGCAAACTGCGTGGTCTGCTGGAA

PANAG_CFBP13505 CAGGGTCCTGAGTACCGCAAAATCTGCGCCCTGGGCGGCAAACTGCGTGGTCTGCTGGAA

TATCI_DSM13699{T} GAGGGGCCTGAATACCAGAAAATCAACGCATTGGGTGAGAAATTACGCGGCCTGATTGAA

TATMO_LMG23360{T} GAGGGGCCTGAGTACCAGAAAATCAACGCATTGGGTGAGAAATTACGTGACCTGATAGAA

TATPT_ATCC33301{T} TACGGACCGGAATACCAGAGAATTAATACACTGGGTGAGCAACTTCGTGGTCTTATCGAA

TATSA_NML06-3099{T} TTTGGCCCGGAATACCAGAAAATTAATGCTTTGGGCGAGCAGTTACGCGGTCTTATCGAA

** ** ** * * ** * ** * * ** * * * **

PANST_CCUG26359{T} GAGGATGCTTACATCGAGCGCGGTGAGCGCCGTCAACCGGTATCCAGCTTCGAACAGGCG

PANST_LMG2632{PT} GAGGATGCTTACATTGAGCGCGGTGAGCGCCGTCAACCGGTATCCAGCTTCGAACAGGCG

PANAL_LMG24248{T} GAAGATGCCTATATCGAACGCGGCGAACGCCGTCAGCCTGTCAGCAGTTTTGAACAGGCG

PANAN_LMG2665{T} GAAGATGCCTATATCGAACGTGGCGAACGCCGTCAGCCCGTCAGCAGTTTTGAACAGGCG

PANAN_97-1 GAAGATGCCTATATCGAACGTGGCGAACGCCGTCAGCCTGTCAGCAGTTTTGAACAGGCG

PANAN_LMG5342 GAAGATGCCTATATCGAACGTGGCGAACGCCGTCAGCCCGTCAGCAGTTTTGAACAGGCG

PANAN_LMG20103 GAAGATGCCTATATCGAACGTGGCGAACGTCGTCAGCCTGTCAGCAGTTTTGAACAGGCG

PANAN_NN08200 GAAGATGCCTATATCGAACGTGGCGAACGTCGTCAGCCTGTCAGCAGTTTTGAACAGGCG

PANAN_ARC311 GAAGATGCCTATATCGAACGTGGCGAACGCCGTCAGCCTGTCAGCAGTTTTGAACAGGCG

PANAN_RSA47 GAAGATGCCTATATCGAACGTGGCGAACGCCGTCAGCCTGTCAGCAGTTTTGAACAGGCG

PANAN_SGAir0210 GAAGATGCCTATATCGAACGTGGCGAACGCCGTCAGCCTGTCAGCAGTTTTGAACAGGCG

MIXCA_DSM22759{T} GAAGACGCCTACATCGAACGCGGCGAGCGCCGTCAGCCGGTCGCCAGCTTCGAGCAGGCG

MIXGA_DSM22758{T} GAAGACGCCTACATTGAGCGCGGCGAACGCCGTCAGCCGGTAGAAAGCTTCGAACAGGCG

MIXAL_LTYR-11Z{T} GAAGATGCCTATATCGAACGCGGCGAACGTCGTCAGCCGGTAGAGAGCTTCGAACAGGCG

MIXTH_QC88-366{T} GAAGATGCTTATATCGAACGCGGCGAACGCCGTCAGCCGATAGAAAGCTTCGAGCAGGCG

PANRO_LMG26273{T} GAAGATGCGTATATCGAACGTGGCGAGCGTCGTCAGCCAGTGGCCAGCTTCGAGCAGGCG

PANRW_LMG26275{T} GAAGATGCGTTTATCGAACGTGGTGATCGCCGTCAGCCAGTGACCAGCTTCGAGCAGGCA

PANCY_LMG2657{T} GAAGACGCCTTTATCGAACGTGGCGAGCGTCGTCAGCCGGTGACCAGTTTCGAACAGGCG

PANEU_LMG5346{T} GAGGACGCCTATATCGAGCGCGGCGAACGTCGTCAGCCGGTTGCCAGCTTCGAGCAGGCG

PANWA_LMG26277{T} GAGGATGCCTTCATCGAGCGTGGCGAGCGCCGTCAGCCGGTAACCAGCTTTGAGCAGGCG

PANDI_CCUG25232{T} GAGGATGCCTATATCGAGCGCGGCGAGCGCCGTCAGCCGGTTGCCAGCTTCGAGCAGGCA

PANSE_LMG5345{T} GAAGACGCCTACATCGAACGCGGCGAGCGCCGTCAGCCGGTCGCCAGCTTCGAGCAGGCT

PANBR_LMG5343{T} GAGGATGCTTACATTGAACGTGGCGAGCGTCGTCAACCGGTAGCCAGCTTCGAGCAGGCG

PANCO_LMG24534{T} GAGGATGCTTACATTGAACGTGGCGAGCGTCGTCAACCGGTGGCCAGCTTCGAGCAGGCG

PANAN_LMG2558{T} GAAGATGCCTACATTGAACGTGGTGAGCGTCGTCAGCCGGTGGCCAGCTTCGAACAGGCG

PANDE_LMG24200{T} GAAGATGCGTACATCGAGCGTGGCGAACGTCGTCAGCCGGTGGCGAGCTTCGAACAGGCG

PANVA_LMG24199{T} GAAGATGCTTACATTGAACGTGGTGAGCGTCGTCAGCCGGTGGCAAGCTTCGAACAGGCG

PANEU_LMG24197{T} GAAGATGCTTACATTGAGCGTGGCGAACGTCGTCAGCCAGTGGCAAGTTTCGAACAGGCG

PANAG_DSM3493{T} GAAGATGCTTACATCGAGCGTGGCGAGCGTCGTCAGCCAGTGGCAAGCTTCGAACAGGCG

PANAG_CFBP13505 GAAGATGCTTACATCGAGCGTGGCGAGCGTCGTCAGCCCGTGGCAAGCTTCGAACAGGCG

TATCI_DSM13699{T} GATGATGCATTTATCGAACGTGGTGAGCGTCGTCAGCCGGTGGCCAGTTTTGAACAGGCG

TATMO_LMG23360{T} GATGATGCGTTTATCGAACGCGGTGAACGCCGTCAGCCGGTGGCCAGTTTTGAACAGGCG

TATPT_ATCC33301{T} GAAGATGCCTTCATTGAACGCGGGGAGCGTCGTCAGCCGGTGGCCAGCTTTGAGCAGGCC

TATSA_NML06-3099{T} GAAGATGCCTTTATCGAACGGGGTGAACGCCGTCAGCCAGTGGCAAGCTTTGAGCAGGCC

** ** ** * ** ** ** ** ** ** ***** ** * ** ** ** *****

PANST_CCUG26359{T} ATTGAGTGGCTGGTGAAAGAATCCCGTCGTGGTCTGTCTGTACAGCGCTATAAAGGTCTG

PANST_LMG2632{PT} ATTGAGTGGCTGGTGAAAGAATCCCGTCGTGGTCTGTCTGTACAGCGCTATAAAGGTCTG

PANAL_LMG24248{T} ATTGACTGGCTGGAGAAAGAGTCGCGTCGCGGTCTGTCGGTCCAGCGCTATAAAGGTCTG

PANAN_LMG2665{T} ATTGACTGGCTGGAGAAAGAGTCACGTCGCGGCCTGTCAGTGCAGCGATATAAAGGTCTG

PANAN_97-1 ATTGACTGGCTGGAGAAAGAGTCGCGTCGCGGCCTGTCAGTGCAGCGATATAAAGGTCTG

PANAN_LMG5342 ATTGACTGGCTGGAGAAAGAGTCACGTCGCGGCCTGTCAGTGCAGCGATATAAAGGTCTG

PANAN_LMG20103 ATTGACTGGCTGGAGAAAGAGTCGCGTCGCGGTCTGTCAGTGCAGCGATATAAAGGTCTG

PANAN_NN08200 ATTGACTGGCTGGAGAAAGAGTCGCGTCGCGGTCTGTCAGTGCAGCGATATAAAGGTCTG

PANAN_ARC311 ATTGACTGGCTGGAGAAAGAGTCGCGTCGCGGTCTGTCAGTGCAGCGATATAAAGGTCTG

PANAN_RSA47 ATTGACTGGCTGGAGAAAGAGTCGCGTCGCGGTCTGTCAGTGCAGCGATATAAAGGTCTG

PANAN_SGAir0210 ATTGACTGGCTGGAGAAAGAGTCGCGTCGCGGTCTGTCAGTACAGCGATATAAAGGTCTG

MIXCA_DSM22759{T} CTGGAGTGGCTGGTGAAAGAGTCGCGTCGCGGCCTGGCAGTACAGCGCTATAAAGGTCTG

MIXGA_DSM22758{T} CTGGAGTGGCTGGTGAAAGAGTCACGTCGTGGCCTGTCGGTACAGCGCTATAAAGGTCTG

MIXAL_LTYR-11Z{T} CTGGAGTGGCTGGTAAAAGAGTCGCGTCGCGGTCTGTCAGTACAGCGTTATAAAGGTCTG

MIXTH_QC88-366{T} CTGGAGTGGCTGGTGAAAGAGTCGCGTCGCGGCCTGTCGGTACAGCGTTATAAAGGTCTG

PANRO_LMG26273{T} ATTGAGTGGCTGGTGAAAGAGTCGCGCCGTGGTCTGTCAGTCCAGCGCTATAAAGGTCTG

PANRW_LMG26275{T} ATTGAGTGGCTGGTGAAAGAGTCGCGTCGTGGTTTGTCAGTCCAGCGTTACAAAGGTCTG

PANCY_LMG2657{T} ATTGAATGGCTGGTGAAAGAGTCGCGTCGCGGTCTGTCAGTACAGCGTTACAAAGGTCTG

PANEU_LMG5346{T} ATTGAGTGGCTGGTGAAAGAGTCGCGCCGTGGTCTCTCCATTCAGCGCTATAAAGGGCTG

PANWA_LMG26277{T} ATTGAGTGGCTGGTGAAGGAGTCCACGCGCGGCCTGTCAGTACAGCGTTATAAAGGTCTG

PANDI_CCUG25232{T} ATTGACTGGCTGGTGAAAGAGTCGCGCCGCGGTCTGTCAGTGCAGCGCTATAAAGGTCTG

PANSE_LMG5345{T} ATCGAATGGCTGGTGAAAGAGTCGCGTCGCGGTCTGTCGGTACAGCGTTATAAAGGTCTG

PANBR_LMG5343{T} ATTGAGTGGCTGGTGAAAGAGTCGCGTCGCGGCCTCTCGGTACAGCGCTACAAAGGTCTG

PANCO_LMG24534{T} ATTGAGTGGCTGGTTAAAGAGTCACGTCGTGGCCTCTCGGTACAGCGCTACAAAGGTCTG

PANAN_LMG2558{T} ATTGAGTGGCTGGTTAAAGAGTCGCGTCGCGGCCTCTCTGTTCAGCGCTACAAAGGTCTG

PANDE_LMG24200{T} ATCGAGTGGCTGGTTAAAGAGTCGCGTCGCGGTCTCTCCGTTCAGCGCTATAAAGGTCTG

PANVA_LMG24199{T} ATTGAGTGGCTGGTGAAAGAGTCGCGTCGCGGCCTGTCGGTCCAGCGCTATAAAGGTCTG

PANEU_LMG24197{T} ATTGAGTGGCTGGTGAAAGAGTCGCGTCGCGGCCTCTCCGTCCAGCGCTACAAAGGTCTG

PANAG_DSM3493{T} ATTGAGTGGCTGATTAAAGAGTCGCGTCGCGGCCTCTCGGTCCAGCGCTACAAAGGTCTG

PANAG_CFBP13505 ATTGAGTGGCTGATTAAAGAGTCGCGTCGCGGCCTCTCGGTCCAGCGTTACAAAGGTCTG

TATCI_DSM13699{T} GTTGAATGGCTGGTGAAAGAATCCCGCCGTGGTCTGTCGGTTCAGCGCTACAAAGGATTA

TATMO_LMG23360{T} GTGGAATGGCTGGTGAAAGAATCCCGTCGAGGCCTGTCGGTCCAGCGCTACAAAGGATTA

TATPT_ATCC33301{T} GTGGAGTGGCTGGTGAAAGAATCCAGACGTGGCCTGTCAGTACAGCGCTATAAAGGTCTG

TATSA_NML06-3099{T} GTTGAATGGCTGGTGAAAGAGTCTCGCCGTGGCCTGTCAGTACAGCGTTATAAAGGTCTG

* ** ****** ** ** ** ** ** * * * ***** ** ***** *

PANST_CCUG26359{T} GGTGAGATGAACCCGGATCAGTTGTGGGAAACGACAATGGACCCAGACAGCCGTCGCATG

PANST_LMG2632{PT} GGTGAGATGAACCCGGATCAGTTGTGGGAAACGACAATGGATCCAGACAGCCGTCGCATG

PANAL_LMG24248{T} GGCGAGATGAACCCGGAGCAGCTGTGGGAAACGACCATGGACCCGGACAGCCGCCGTATG

PANAN_LMG2665{T} GGCGAAATGAACCCAGAGCAGCTGTGGGAAACCACCATGGATCCGGACAGCCGCCGTATG

PANAN_97-1 GGCGAAATGAACCCAGAGCAGCTGTGGGAAACCACCATGGATCCGGACAGCCGCCGTATG

PANAN_LMG5342 GGCGAAATGAACCCAGAGCAGCTGTGGGAAACCACCATGGATCCGGACAGCCGCCGTATG

PANAN_LMG20103 GGCGAAATGAACCCAGAGCAGCTGTGGGAAACCACCATGGATCCGGACAGCCGCCGTATG

PANAN_NN08200 GGCGAAATGAACCCAGAGCAGCTGTGGGAAACCACCATGGATCCGGACAGCCGCCGTATG

PANAN_ARC311 GGCGAAATGAACCCAGAGCAGCTGTGGGAAACCACCATGGATCCGGACAGCCGCCGTATG

PANAN_RSA47 GGCGAAATGAACCCAGATCAGCTGTGGGAAACCACCATGGATCCGGACAGCCGCCGTATG

PANAN_SGAir0210 GGCGAAATGAACCCAGATCAGCTGTGGGAAACCACCATGGATCCGGACAGCCGCCGTATG

MIXCA_DSM22759{T} GGTGAGATGAACCCGGAACAGCTGTGGGAAACCACTATGGACCCGGACAGCCGTCGCATG

MIXGA_DSM22758{T} GGTGAGATGAACCCGGAACAGCTGTGGGAAACCACCATGGATCCGGACAGCCGCCGTATG

MIXAL_LTYR-11Z{T} GGCGAGATGAACCCGGAGCAGCTGTGGGAAACCACTATGGATCCGGATAGCCGTCGCATG

MIXTH_QC88-366{T} GGCGAGATGAACCCGGAGCAGCTGTGGGAAACCACTATGGATCCGGATAGCCGCCGTATG

PANRO_LMG26273{T} GGCGAGATGAACCCAGAGCAGCTGTGGGAAACCACCATGGATCCAGACAGTCGTCGCATG

PANRW_LMG26275{T} GGCGAAATGAACCCAGAGCAGCTGTGGGAAACCACCATGGATCCAGACAGCCGCCGCATG

PANCY_LMG2657{T} GGCGAGATGAACCCGGAGCAGCTGTGGGAAACCACCATGGACCCGGACAGCCGTCGTATG

PANEU_LMG5346{T} GGCGAGATGAACCCGGAGCAGCTGTGGGAAACCACGATGGATCCGGACAGCCGCCGTATG

PANWA_LMG26277{T} GGCGAGATGAACCCGGATCAGCTGTGGGAAACCACCATGGACCCGGACAGCCGCCGTATG

PANDI_CCUG25232{T} GGCGAGATGAACCCGGATCAGCTGTGGGAAACCACCATGGATCCGGACAGCCGCCGCATG

PANSE_LMG5345{T} GGCGAGATGAACGCCGAGCAGCTGTGGGAAACCACGATGGATCCGGACAGCCGTCGCATG

PANBR_LMG5343{T} GGCGAGATGAACCCTGAACAGCTGTGGGAAACCACCATGGATCCGGATAGCCGTCGTATG

PANCO_LMG24534{T} GGTGAGATGAACCCGGAGCAGCTGTGGGAAACCACCATGGACCCGGATAGCCGTCGTATG

PANAN_LMG2558{T} GGCGAGATGAACCCGGATCAGCTGTGGGAAACCACCATGGACCCGGACAGCCGTCGTATG

PANDE_LMG24200{T} GGCGAGATGAACCCGGATCAGCTGTGGGAAACCACCATGGATCCCGACAGCCGCCGTATG

PANVA_LMG24199{T} GGCGAAATGAACCCGGATCAGCTGTGGGAAACCACCATGGATCCAGACAGCCGTCGTATG

PANEU_LMG24197{T} GGTGAGATGAACCCGGATCAGCTGTGGGAAACCACCATGGATCCAGACAGCCGTCGTATG

PANAG_DSM3493{T} GGCGAGATGAACCCGGAGCAGCTGTGGGAAACCACCATGGATCCAGACAGCCGTCGCATG

PANAG_CFBP13505 GGCGAGATGAACCCGGAGCAGCTGTGGGAAACCACCATGGATCCAGACAGCCGTCGCATG

TATCI_DSM13699{T} GGCGAGATGAACCCTGAACAGTTGTGGGAAACCACAATGGATCCGGATAGCCGCCGTATG

TATMO_LMG23360{T} GGCGAGATGAACCCTGAGCAGTTGTGGGAAACCACAATGGATCCGGATAGCCGTCGTATG

TATPT_ATCC33301{T} GGTGAGATGAACCCGGAACAGCTCTGGGAAACGACAATGGATCCGGAAAGCCGCCGCATG

TATSA_NML06-3099{T} GGTGAGATGAACCCAGAACAGTTGTGGGAAACCACTATGGATCCGGAAAGCCGTCGTATG

** ** ****** * ** *** * ******** ** ***** ** ** ** ** ** ***

PANST_CCUG26359{T} CTACGTGTCACCATTAAAGATGCGATCGCCGCCGACCAGCTGTTTACAACCCTGATGGGG

PANST_LMG2632{PT} CTGCGTGTCACCATTAAAGATGCGATCGCCGCCGACCAGCTGTTTACAACCCTGATGGGG

PANAL_LMG24248{T} TTACGCGTGACCATCAAAGATGCGATTGCCGCCGATCAGCTGTTCACCACACTGATGGGC

PANAN_LMG2665{T} TTACGCGTGACCATCAAAGATGCGATTGCCGCCGATCAGCTGTTCACCACGTTGATGGGT

PANAN_97-1 TTACGCGTGACCATCAAAGATGCGATTGCCGCCGATCAGCTGTTCACCACGTTGATGGGT

PANAN_LMG5342 TTACGCGTGACCATCAAAGATGCGATTGCCGCCGATCAGCTGTTCACCACGTTGATGGGT

PANAN_LMG20103 TTACGCGTGACCATCAAAGATGCGATTGCCGCCGATCAGCTGTTCACCACGTTGATGGGT

PANAN_NN08200 TTACGCGTGACCATCAAAGATGCGATTGCCGCCGATCAGCTGTTCACCACGTTGATGGGT

PANAN_ARC311 TTACGCGTGACCATCAAAGATGCGATTGCCGCCGATCAGCTGTTCACCACGTTGATGGGT

PANAN_RSA47 TTACGCGTGACCATCAAAGATGCGATTGCCGCCGATCAGCTGTTCACCACGTTGATGGGT

PANAN_SGAir0210 TTACGCGTGACCATCAAAGATGCGATTGCCGCCGATCAGCTGTTCACCACGTTGATGGGT

MIXCA_DSM22759{T} CTGCGTGTTACGATTAAAGATGCGATCGCCGCCGATCAGCTGTTTACCACGCTGATGGGC

MIXGA_DSM22758{T} CTGCGCGTCACCATCAAAGATGCCATCGCCGCCGACCAGCTGTTCACCACGCTAATGGGC

MIXAL_LTYR-11Z{T} CTGCGCGTAACCATTAAAGACGCGATTGCCGCCGATCAGCTGTTTACCACGCTGATGGGC

MIXTH_QC88-366{T} CTGCGCGTAACGATTAAAGATGCGATTGCTGCCGATCAGCTGTTTACCACGCTGATGGGC

PANRO_LMG26273{T} CTGCGCGTCACCATCAAAGATGCCATCGCTGCTGACCAGCTGTTCACCACGCTGATGGGC

PANRW_LMG26275{T} CTGCGCGTCACCATTAAAGATGCCATCGCGGCTGACCAGCTGTTCACCACGCTGATGGGC

PANCY_LMG2657{T} CTGCGCGTCACCATCAAAGACGCCATTGCTGCCGATCAGCTGTTCACCACGCTGATGGGC

PANEU_LMG5346{T} CTGCGCGTTACCATTAAGGATGCGATTGCTGCCGATCAGCTCTTTACCACGCTGATGGGC

PANWA_LMG26277{T} CTGCGCGTCACCATCAAGGACGCGATTGCTGCTGACCAGCTGTTTACTACGCTGATGGGC

PANDI_CCUG25232{T} CTGCGCGTCACCATCAAAGACGCCATCGCCGCCGACCAGCTGTTCACCACGCTGATGGGC

PANSE_LMG5345{T} TTGCGCGTCACCATCAAGGACGCCATTGCCGCCGATCAGCTCTTCACTACGCTGATGGGC

PANBR_LMG5343{T} CTGCGCGTTACCATTAAAGATGCGATTGGCGCTGACCAGCTATTTACTACCCTGATGGGC

PANCO_LMG24534{T} CTGCGCGTGACCATTAAAGATGCGATTGGCGCGGATCAGCTGTTTACTACCCTGATGGGC

PANAN_LMG2558{T} CTGCGCGTCACCATCAAAGATGCCATTGGTGCTGACCAGCTGTTCACTACGCTGATGGGC

PANDE_LMG24200{T} CTGCGCGTCACCATTAAAGATGCGATCGGTGCCGATCAGCTCTTCACGACCCTGATGGGC

PANVA_LMG24199{T} CTGCGCGTCACCATTAAAGATGCGATTGGTGCTGACCAGCTGTTCACGACCCTGATGGGC

PANEU_LMG24197{T} CTGCGCGTCACCATTAAAGATGCGATTGGCGCTGACCAGCTGTTCACTACGTTGATGGGC

PANAG_DSM3493{T} CTGCGCGTTACCATTAAAGATGCGATTGGCGCTGATCAGTTGTTCACTACCCTGATGGGC

PANAG_CFBP13505 CTGCGCGTCACCATTAAAGATGCGATTGGCGCTGATCAGCTGTTCACTACCCTGATGGGC

TATCI_DSM13699{T} TTGCGGGTTACCATTAAAGATGCGATTGCAGCCGATCAGTTGTTCACCACCCTGATGGGA

TATMO_LMG23360{T} CTGCGGGTTACCATTAAAGATGCGATTGCCGCCGATCAGCTGTTCACCACCCTGATGGGC

TATPT_ATCC33301{T} ATGCGTGTGACTATAAAAGATGCCATTGCCGCCGATCAGCTGTTTACCACGCTGATGGGG

TATSA_NML06-3099{T} ATGCGCGTTACCATCAAAGATGCTATCGCCGCAGATCAGCTGTTTACCACCTTAATGGGG

* ** ** ** ** ** ** ** ** * ** ** *** * ** ** ** * *****

PANST_CCUG26359{T} GATGCCGTTGAACCACGCCGCGCCTTCATCGAAGAGAATGCGCTGAAAGCCGCTAACATC

PANST_LMG2632{PT} GATGCCGTTGAACCACGCCGCGCCTTCATCGAAGAGAATGCGTTGAAAGCCGCAAACATC

PANAL_LMG24248{T} GACGCGGTTGAACCACGCCGCGCCTTCATAGAAGAGAATGCGCTGAAAGCCGCAAATATT

PANAN_LMG2665{T} GACGCGGTTGAACCGCGCCGCGCCTTCATTGAAGAGAATGCGCTGAAAGCCGCAAATATT

PANAN_97-1 GACGCGGTTGAACCGCGCCGCGCCTTCATTGAAGAGAATGCGCTGAAAGCCGCAAATATT

PANAN_LMG5342 GACGCGGTTGAACCGCGCCGCGCGTTCATTGAAGAGAATGCGCTGAAAGCCGCAAATATT

PANAN_LMG20103 GACGCGGTTGAACCGCGCCGCGCGTTCATTGAAGAGAATGCGCTGAAAGCCGCAAATATT

PANAN_NN08200 GACGCGGTTGAACCGCGCCGCGCGTTCATTGAAGAGAATGCGCTGAAAGCCGCAAATATT

PANAN_ARC311 GACGCGGTTGAACCGCGCCGCGCCTTCATTGAAGAGAATGCGCTGAAAGCCGCAAATATT

PANAN_RSA47 GACGCGGTTGAACCGCGCCGCGCCTTCATTGAAGAGAATGCGCTGAAAGCCGCAAATATT

PANAN_SGAir0210 GACGCGGTTGAACCGCGCCGCGCCTTCATTGAAGAGAATGCGCTGAAAGCCGCAAATATT

MIXCA_DSM22759{T} GATGCGGTTGAACCGCGCCGCGCCTTTATCGAAGAGAACGCCCTGAAAGCGGCGAATATC

MIXGA_DSM22758{T} GATGCGGTTGAGCCGCGCCGCGCCTTTATCGAAGAGAACGCCCTGAAAGCCGCTAACATC

MIXAL_LTYR-11Z{T} GATGCGGTTGAGCCGCGCCGCGCCTTTATTGAAGAAAACGCCCTGAAAGCGGCCAATATC

MIXTH_QC88-366{T} GATGCGGTAGAACCACGCCGCGCCTTTATCGAAGAGAACGCCCTGAAAGCGGCCAATATC

PANRO_LMG26273{T} GATGCGGTAGAACCGCGCCGCGCCTTCATCGAAGAGAACGCGCTGAAAGCCGCTAACATC

PANRW_LMG26275{T} GATGCGGTTGAACCACGCCGTGCCTTCATTGAAGAGAACGCGCTGAAAGCGGCCAACATC

PANCY_LMG2657{T} GATGCGGTAGAACCGCGCCGCGCCTTCATCGAAGAGAACGCGCTGAAAGCGGCCAATATC

PANEU_LMG5346{T} GATGCGGTGGAGCCGCGTCGCGCCTTTATCGAAGAGAACGCGCTGAAAGCCGCAAATATC

PANWA_LMG26277{T} GATGCGGTTGAGCCGCGCCGCGCCTTCATCGAAGAGAATGCGCTGAAAGCTGCCAACATC

PANDI_CCUG25232{T} GATGCGGTTGAACCGCGCCGCGCCTTCATCGAAGAGAACGCGCTGAAAGCCGCCAACATC

PANSE_LMG5345{T} GATGCGGTTGAGCCGCGCCGCGCCTTTATCGAAGAGAACGCCCTGAAAGCGGCGAACATC

PANBR_LMG5343{T} GATGCGGTTGAACCGCGCCGTGCCTTCATCGAAGAGAATGCGCTGAAAGCCGCCAATATC

PANCO_LMG24534{T} GATGCGGTTGAACCGCGCCGCGCCTTCATCGAAGAGAATGCGCTGAAAGCCGCCAACATC

PANAN_LMG2558{T} GATGCGGTTGAACCGCGCCGCGCCTTCATCGAAGAGAATGCGCTGAAAGCAGCCAATATC

PANDE_LMG24200{T} GATGCGGTCGAACCGCGCCGCGCCTTCATCGAAGAGAATGCGCTGAAAGCGGCTAACATC

PANVA_LMG24199{T} GATGCGGTCGAACCGCGTCGCGCCTTCATCGAAGAGAATGCGCTGAAAGCGGCCAATATC

PANEU_LMG24197{T} GATGCGGTGGAACCGCGTCGCGCCTTCATCGAAGAGAATGCGCTGAAAGCCGCCAATATC

PANAG_DSM3493{T} GATGCGGTTGAACCGCGCCGCGCCTTCATCGAAGAAAATGCGCTGAAAGCCGCCAATATC

PANAG_CFBP13505 GATGCGGTCGAACCGCGCCGCGCCTTCATCGAAGAAAATGCGCTGAAAGCCGCCAATATC

TATCI_DSM13699{T} GATGCGGTTGAACCACGCCGGGCCTTTATTGAAGAAAATGCCCTGAAAGCAGCCAATATC

TATMO_LMG23360{T} GACGCGGTTGAACCACGCAGAGCCTTTATCGAAGAGAATGCGCTGAAAGCAGCCAATATC

TATPT_ATCC33301{T} GATGCGGTTGAACCTCGTCGCGCCTTTATCGAAGAAAATGCCCTGAAAGCCGCGAATATC

TATSA_NML06-3099{T} GATGCTGTGGAACCACGTCGCGCCTTTATCGAAGAAAACGCCTTAAAAGCGGCGAATATC

** ** ** ** ** ** * ** ** ** ***** ** ** * ***** ** ** **

PANST_CCUG26359{T} GATATCTAA

PANST_LMG2632{PT} GATATCTAA

PANAL_LMG24248{T} GATATCTAA

PANAN_LMG2665{T} GATATCTAA

PANAN_97-1 GATATCTAA

PANAN_LMG5342 GATATCTAA

PANAN_LMG20103 GATATCTAA

PANAN_NN08200 GATATCTAA

PANAN_ARC311 GATATCTAA

PANAN_RSA47 GATATCTAA

PANAN_SGAir0210 GATATCTAA

MIXCA_DSM22759{T} GATATTTAA

MIXGA_DSM22758{T} GATATCTGA

MIXAL_LTYR-11Z{T} GATATTTAA

MIXTH_QC88-366{T} GATATTTAA

PANRO_LMG26273{T} GATATCTGA

PANRW_LMG26275{T} GATATCTAA

PANCY_LMG2657{T} GATATCTGA

PANEU_LMG5346{T} GATATCTGA

PANWA_LMG26277{T} GATATCTGA

PANDI_CCUG25232{T} GATATCTGA

PANSE_LMG5345{T} GATATTTAA

PANBR_LMG5343{T} GATATCTAA

PANCO_LMG24534{T} GATATTTAA

PANAN_LMG2558{T} GATATCTGA

PANDE_LMG24200{T} GACATTTAA

PANVA_LMG24199{T} GATATTTAA

PANEU_LMG24197{T} GATATCTAA

PANAG_DSM3493{T} GATATTTAA

PANAG_CFBP13505 GATATTTAA

TATCI_DSM13699{T} GATATTTAA

TATMO_LMG23360{T} GATATTTAA

TATPT_ATCC33301{T} GATATCTGA

TATSA_NML06-3099{T} GATATCTAA

** ** * *
