# Supplementary material for: Development of two loop-mediated isothermal amplification (LAMP) genomics-informed diagnostic protocols for rapid detection of Pantoea species on rice
Source: MethodsX. 2021 Jan 6;8:101216. doi: 10.1016/j.mex.2021.101216 (PMC8374213; doi:10.1016/j.mex.2021.101216)
Supplement: Supplementary file 2 [file mmc2.docx]

SPHME_DAPP-PG224{T} ATGGCAACCGCCGCAGACGACATCCGCCCGACCCAGTCGACGGGCAGCAGCAACACCACC

SPHPA_NCTC11030{T} ATGGCAACCGCCGCTGAGGACATCCGGCCGACCACGGC---------CACCAACAATGTC

PANAN_LMG2558{T} ATGGCAA---------------------------------------------------CT

PANBR_LMG5343{T} ATGGCAA---------------------------------------------------CT

PANCO_LMG24534{T} ATGGCAA---------------------------------------------------CT

PANDE_LMG24200{T} ATGGCAA---------------------------------------------------CT

PANVA_LMG24199{T} ATGGCAA---------------------------------------------------CT

PANEU_LMG24197{T} ATGGCAA---------------------------------------------------CT

PANAG_DSM3493{T} ATGGCAA---------------------------------------------------CT

PANAG_CFBP13505 ATGGCAA---------------------------------------------------CT

MIXAL_LTYR-11Z{T} ATGGCAA---------------------------------------------------CT

MIXTH_QC88-366{T} ATGGCAA---------------------------------------------------CT

MIXCA_DSM22759{T} ATGGCAA---------------------------------------------------CT

MIXGA_DSM22758{T} ATGGCAA---------------------------------------------------CT

PANSE_LMG5345{T} ATGGCAG---------------------------------------------------CT

PANEU_LMG5346{T} ATGGCAG---------------------------------------------------CT

PANDI_CCUG25232{T} ATGGCAG---------------------------------------------------CT

PANWA_LMG26277{T} ATGGCAG---------------------------------------------------CT

PANRO_LMG26273{T} ATGGCAG---------------------------------------------------CT

PANRW_LMG26275{T} ATGGCAG---------------------------------------------------CT

PANCY_LMG2657{T} ATGGCAG---------------------------------------------------CT

PANST_CCUG26359{T} ATGGCAG---------------------------------------------------CT

PANST_LMG2632{PT} ATGGCAG---------------------------------------------------CT

PANAL_LMG24248{T} ATGGCAG---------------------------------------------------CT

PANAN_LMG2665{T} ATGGCAG---------------------------------------------------CT

PANAN_RSA47 ATGGCAG---------------------------------------------------CT

TATSA_NML06-3099{T} ATGGCAG---------------------------------------------------CT

TATPT_ATCC33301{T} ATGGCAG---------------------------------------------------CT

TATCI_DSM13699{T} ATGGCAG---------------------------------------------------CT

TATMO_LMG23360{T} ATGGCAG---------------------------------------------------CT

BURGL_LMG2196{T} ATGAGTACTACTGCTTTGGTAG------------------------------------AA

XANOR_ATCC35933{T} ATGAGTC---------------------------------------------------AG

XANOR_CFBP2286{PT} ATGAGTC---------------------------------------------------AG

PSEFU_LMG2158{T} ATGAGTA---------------------------------------------------GC

PSEOR_KCTC32247 ATGAGTA---------------------------------------------------GC

***

SPHME_DAPP-PG224{T} GGCACGATCGCGCAGGTCATCGGCGCCGTCGTCGACGTCCACTTCCC---CGAAACGCTG

SPHPA_NCTC11030{T} GGCACGATCGCCCAGGTCATCGGCGCGGTCGTCGACGTTCACTTCCC-CGAGAACCTGC-

PANAN_LMG2558{T} GGAAAGATTGTCCAGATTATCGGCGCCGTTGTTGACGTCGAATTCCCTCAGGACGCGGTA

PANBR_LMG5343{T} GGAAAGATTGTCCAGATTATCGGCGCCGTTGTTGACGTCGAATTCCCTCAGGACGCGGTA

PANCO_LMG24534{T} GGAAAGATTGTCCAGATTATCGGCGCCGTTGTTGACGTCGAATTCCCTCAGGACGCGGTA

PANDE_LMG24200{T} GGAAAGATTGTCCAGATTATCGGCGCCGTTGTTGACGTCGAATTCCCTCAGGACGCGGTA

PANVA_LMG24199{T} GGAAAGATTGTCCAGATTATCGGCGCCGTTGTTGACGTCGAATTCCCTCAGGACGCGGTA

PANEU_LMG24197{T} GGAAAGATTGTCCAGATTATCGGCGCCGTTGTTGACGTCGAATTCCCTCAGGACGCGGTA

PANAG_DSM3493{T} GGAAAGATTGTCCAGATTATCGGCGCCGTTGTTGACGTCGAATTCCCTCAGGACGCGGTA

PANAG_CFBP13505 GGAAAGATTGTCCAGATTATCGGCGCCGTTGTTGACGTCGAATTCCCTCAGGACGCGGTA

MIXAL_LTYR-11Z{T} GGAAAGATTGTCCAGATCATCGGCGCCGTAGTTGACGTCGAGTTCCCTCAGGACGCTGTA

MIXTH_QC88-366{T} GGAAAGATTGTCCAGATCATCGGCGCCGTAGTTGACGTCGAGTTCCCTCAGGACGCTGTA

MIXCA_DSM22759{T} GGAAAGATTGTCCAGATCATCGGCGCCGTAGTTGACGTCGAGTTCCCTCAGGACGCTGTA

MIXGA_DSM22758{T} GGAAAGATTGTCCAGATCATCGGCGCCGTAGTTGACGTCGAATTCCCTCAGGACGCTGTA

PANSE_LMG5345{T} GGAAAGATTGTCCAGATTATCGGCGCCGTAGTTGACGTCGAATTCCCTCAGGACGCGGTA

PANEU_LMG5346{T} GGAAAGATTGTCCAGATTATCGGCGCCGTAGTTGACGTCGAATTCCCTCAGGACGCGGTA

PANDI_CCUG25232{T} GGAAAGATTGTCCAGATTATCGGCGCCGTAGTTGACGTCGAATTCCCTCAGGACGCGGTA

PANWA_LMG26277{T} GGAAAGATTGTCCAGATTATCGGCGCCGTAGTTGACGTCGAATTCCCTCAGGACGCAGTA

PANRO_LMG26273{T} GGAAAGATTGTCCAGATTATCGGCGCCGTAGTTGACGTCGAATTCCCTCAGGACGCGGTA

PANRW_LMG26275{T} GGAAAGATTGTCCAGATTATCGGCGCCGTAGTTGACGTCGAATTCCCTCAGGACGCGGTA

PANCY_LMG2657{T} GGAAAGATTGTCCAGATTATCGGCGCCGTAGTTGACGTCGAATTCCCTCAGGATGCAGTA

PANST_CCUG26359{T} GGAAAGATTGTCCAGATTATCGGCGCCGTAGTTGACGTCGAATTCCCTCAGGATGCAGTA

PANST_LMG2632{PT} GGAAAGATTGTCCAGATTATCGGCGCCGTAGTTGACGTCGAATTCCCTCAGGATGCAGTA

PANAL_LMG24248{T} GGAAAGATTGTCCAGATTATCGGCGCCGTAGTTGACGTCGAATTCCCTCAGGATGCAGTA

PANAN_LMG2665{T} GGAAAGATTGTCCAGATTATCGGCGCCGTAGTTGACGTCGAATTCCCTCAGGATGCAGTA

PANAN_RSA47 GGAAAGATTGTCCAGATTATCGGCGCCGTAGTTGACGTCGAATTCCCTCAGGATGCAGTA

TATSA_NML06-3099{T} GGAAAGATTGTCCAGATTATCGGCGCCGTAGTTGACGTCGAGTTCCCTCAGGACGCTGTA

TATPT_ATCC33301{T} GGAAAGATTGTCCAGATTATCGGCGCCGTAGTTGACGTCGAGTTCCCTCAGGACGCTGTA

TATCI_DSM13699{T} GGAAAGATTGTCCAGATTATCGGCGCCGTAGTTGACGTCGAGTTCCCTCAGGAAGCTGTA

TATMO_LMG23360{T} GGAAAGATTGTCCAGATTATCGGCGCCGTAGTTGACGTCGAGTTCCCTCAGGAAGCTGTA

BURGL_LMG2196{T} GGCAAGATCGTACAGTGCATCGGCGCCGTTATCGACGTGGAATTCCCGCGCGAAAGCATG

XANOR_ATCC35933{T} GGCAAGATCGTTCAGATCATCGGCGCGGTCGTCGACGTCGAATTCCCGCGCAATGAAGTG

XANOR_CFBP2286{PT} GGCAAGATCGTTCAGATCATCGGCGCGGTCGTCGACGTCGAATTCCCGCGCAATGAAGTG

PSEFU_LMG2158{T} GGACGTATCGTTCAAATCATCGGCGCCGTCATTGACGTGGAATTTCCACGCGACAGCGTA

PSEOR_KCTC32247 GGACGTATCGTTCAAATCATCGGCGCCGTTATCGACGTGGAATTCCCGCGCGATCAGGTG

** ** * ** ******** ** * ***** * ** ** *

SPHME_DAPP-PG224{T} CCGGCGATCCTGTCGGCGCTGGAAACGGACAACAACGGCACCAAGCTGGTGCTCGAGGTT

SPHPA_NCTC11030{T} CCGCGATTCTGTCG-GCGCTGGAGACGGACAATAACGGCACCCGCCTGGTGCTCGAAGTC

PANAN_LMG2558{T} CCGCAAGTGTACAGCGCCCTCGAGGTGATGAATGGTGATGCACGTCTGGTGCTGGAAGTT

PANBR_LMG5343{T} CCGCAAGTGTACAGTGCCCTCGAGGTTATGAATGGTGATGCGCGTCTGGTGCTGGAAGTT

PANCO_LMG24534{T} CCGCAAGTGTACAGTGCCCTCGAGGTTATGAATGGTGATGCGCGTCTGGTGCTGGAAGTT

PANDE_LMG24200{T} CCGCAAGTGTACAGCGCCCTCGAGGTGATGAATGGTGATGCGCGTCTGGTGCTGGAAGTT

PANVA_LMG24199{T} CCGCAAGTGTACAGCGCTCTCGAGGTGATGAATGGTGATGCGCGTCTGGTGCTGGAAGTA

PANEU_LMG24197{T} CCGCAAGTGTACAGCGCCCTCGAGGTTATGAATGGTGATGCGCGTCTGGTGCTGGAAGTA

PANAG_DSM3493{T} CCGCAAGTGTACAGCGCCCTCGAGGTTATGAATGGTGATGCGCGTCTGGTGCTGGAAGTT

PANAG_CFBP13505 CCGCAAGTGTACAGCGCCCTCGAGGTTATGAATGGTGATGCGCGTCTGGTGCTGGAAGTT

MIXAL_LTYR-11Z{T} CCGCAGGTGTACAACGCCCTTGAGGTTAAAAATGGTGATGCGCGTCTGGTGCTGGAAGTT

MIXTH_QC88-366{T} CCGCAGGTGTACAACGCCCTTGAGGTTAAAAATGGTGATGCGCGTCTGGTGCTGGAAGTA

MIXCA_DSM22759{T} CCGCAGGTGTACAACGCCCTTGAGGTTAAAAATGGTGATGCGCGTCTGGTGCTGGAAGTG

MIXGA_DSM22758{T} CCGCAGGTGTACAACGCCCTTGAGGTTAAAAATGGTGATGCGCGTCTGGTGCTGGAAGTG

PANSE_LMG5345{T} CCGCAAGTGTACAACGCCCTTGAGGTTCACAATGGTGATGCTCGTCTGGTGCTGGAAGTT

PANEU_LMG5346{T} CCACAGGTATACAGCGCTCTCGAGGTTAAGAATGGTGATACTCGTCTGGTGCTGGAAGTT

PANDI_CCUG25232{T} CCGCAGGTATACAGCGCTCTCGAGGTAAAAAATGGTGATGCTCGTCTGGTGCTGGAAGTA

PANWA_LMG26277{T} CCGCAGGTATATAGCGCTCTCGAGGTTAAGAATGGTGATGCTCGTCTGGTGCTGGAAGTT

PANRO_LMG26273{T} CCGCAGGTATATAGCGCGCTTGAGGTTCAAAATGGTGATGCTCGTCTGGTGCTGGAAGTT

PANRW_LMG26275{T} CCGCAGGTATATAGCGCGCTTGAGGTTCAAAATGGTGACGCTCGTCTGGTGCTGGAAGTT

PANCY_LMG2657{T} CCGCAAGTGTACAGCGCCCTTGAGGTTAAGAATGGTGATGCTCGTCTGGTGCTGGAAGTT

PANST_CCUG26359{T} CCGCAAGTGTACAGCGCTCTTGAGGTTAAGAATGGTGATGCTCGTCTGGTGCTTGAAGTT

PANST_LMG2632{PT} CCGCAAGTGTACAGCGCTCTTGAGGTTAAGAATGGTGATGCTCGTCTGGTGCTGGAAGTT

PANAL_LMG24248{T} CCGCAAGTGTACAGCGCTCTTGAGGTTATGAATGGTGATGCTCGTCTGGTGCTGGAAGTT

PANAN_LMG2665{T} CCGCAAGTGTACAGCGCTCTTGAGGTTAAGAATGGTGATGCTCGTCTGGTGCTGGAAGTT

PANAN_RSA47 CCGCAAGTGTACAGCGCTCTTGAGGTTAAGAATGGTGATGCTCGTCTGGTGCTGGAAGTT

TATSA_NML06-3099{T} CCACAGGTGTACAACGCTCTTGAGGTTGAAAATGGTGATACCCGTCTGGTGCTGGAAGTT

TATPT_ATCC33301{T} CCACAGGTGTACAACGCTCTTGAGGTTGAAAATGGTGATACCCGTCTGGTGCTGGAAGTT

TATCI_DSM13699{T} CCACAGGTGTACAATGCTCTTGAGGTTGAAAACGGTAATACCCCTCTGGTGCTGGAAGTT

TATMO_LMG23360{T} CCGCAGGTGTACAACGCTCTTGAGGTTGAAAATGGTAATACCCCTCTGGTGCTGGAAGTT

BURGL_LMG2196{T} CCGAAGATCTACGACGCGCTGACGCTGGAAGGTACGGAG------CTGACGCTCGAAGTC

XANOR_ATCC35933{T} CCGAAGGTGTATCACGCGCTGAAGGTCGAAGGCACCGAAATCACCCTG------GAAGTG

XANOR_CFBP2286{PT} CCGAAGGTGTATCACGCGCTGAAGGTCGAAGGCACCGAAATCACCCTG------GAAGTG

PSEFU_LMG2158{T} CCGAGCATCTACGACGCCTTGAAGGTTCAAGGCGCCGAAACCACTCTG------GAAGTT

PSEOR_KCTC32247 CCGAACGTCTATGACGCGCTGAAGGTAGAAGGCGCGGCCACCACCCTG------GAAGTC

** * ** * *** ** **

SPHME_DAPP-PG224{T} GCGCAGCACCTGGGCGAGAACACCGTCCGCACGATCGCGATGGACGCGACCGAGGGCCTG

SPHPA_NCTC11030{T} GCGCAGCATCTGGGCGAAAACACCGTCCGCACGATCGCGATGGACACGACCGAGGGCCTG

PANAN_LMG2558{T} CAGCAGCAGCTGGGCGGCGGCGTTGTGCGTACCATCGCAATGGGTACGTCTGACGGCCTG

PANBR_LMG5343{T} CAGCAGCAGCTGGGCGGCGGCGTGGTCCGTACCATTGCAATGGGTACGTCTGACGGCCTG

PANCO_LMG24534{T} CAGCAGCAGCTGGGCGGCGGCGTGGTCCGTACCATTGCAATGGGTACGTCTGACGGCCTG

PANDE_LMG24200{T} CAGCAGCAGCTGGGCGGCGGCGTAGTGCGTACCATTGCAATGGGTACGTCTGACGGCCTG

PANVA_LMG24199{T} CAGCAGCAGCTCGGCGGCGGCGTAGTTCGTACCATCGCAATGGGTACGTCTGACGGCCTG

PANEU_LMG24197{T} CAGCAGCAGCTGGGCGGCGGCGTAGTACGTACCATCGCAATGGGTACGTCTGACGGCCTG

PANAG_DSM3493{T} CAGCAGCAGCTCGGCGGCGGCGTAGTACGTACCATCGCAATGGGTACGTCTGACGGCCTG

PANAG_CFBP13505 CAGCAACAGCTCGGCGGCGGCGTAGTACGTACCATCGCAATGGGTACGTCTGACGGCCTG

MIXAL_LTYR-11Z{T} CAACAGCAGCTGGGCGGCGGCGTGGTTCGTACCATCGCTATGGGTACTTCTGACGGCCTG

MIXTH_QC88-366{T} CAACAGCAGCTGGGCGGCGGCGTGGTTCGTACCATCGCTATGGGTACTTCTGACGGCCTG

MIXCA_DSM22759{T} CAGCAGCAGCTGGGCGGCGGCGTAGTACGTACCATCGCAATGGGTACGTCTGACGGCCTG

MIXGA_DSM22758{T} CAGCAGCAGCTGGGCGGCGGCGTGGTTCGTACCATCGCGATGGGTACGTCTGACGGCCTG

PANSE_LMG5345{T} CAGCAGCAGCTGGGCGGCGGCGTGGTTCGTACCATCGCCATGGGTTCTTCTGACGGCCTG

PANEU_LMG5346{T} CAGCAGCAGCTGGGTGGTGGCGTAGTACGTACTATCGCCATGGGTTCTTCTGACGGCCTG

PANDI_CCUG25232{T} CAGCAGCAGCTGGGCGGTGGCGTGGTGCGTACCATCGCCATGGGTTCTTCTGACGGCCTG

PANWA_LMG26277{T} CAGCAGCAGCTGGGCGGTGGCGTGGTGCGTACCATCGCCATGGGTACTTCTGACGGCCTG

PANRO_LMG26273{T} CAGCAGCAGCTGGGCGGTGGCGTGGTACGTACCATCGCCATGGGTACTTCTGATGGCCTG

PANRW_LMG26275{T} CAGCAGCAGCTGGGTGGTGGCGTGGTACGTACCATCGCCATGGGTACTTCTGACGGCCTG

PANCY_LMG2657{T} CAGCAGCAGCTGGGTGGTGGCGTGGTTCGTACCATCGCCATGGGTACTTCTGACGGCCTG

PANST_CCUG26359{T} CAGCAGCAGCTGGGTGGTGGCGTAGTGCGTACTATCGCCATGGGTACTTCTGACGGCCTG

PANST_LMG2632{PT} CAGCAGCAGCTGGGTGGTGGCGTAGTGCGTACTATCGCCATGGGTACTTCTGACGGCCTG

PANAL_LMG24248{T} CAGCAGCAGCTGGGTGGTGGCGTGGTTCGTACCATCGCCATGGGTACTTCTGACGGCCTG

PANAN_LMG2665{T} CAGCAGCAGCTGGGTGGTGGCGTGGTTCGTACTATCGCCATGGGTACTTCTGACGGCCTG

PANAN_RSA47 CAGCAGCAGCTGGGTGGTGGCGTGGTTCGTACCATCGCTATGGGTACTTCTGACGGCCTG

TATSA_NML06-3099{T} CAGCAGCAGTTGGGCGGTGGTGTCGTTCGTGCGATTGCAATGGGTTCTTCTGACGGTCTG

TATPT_ATCC33301{T} CAGCAGCAGCTGGGCGGTGGTGTCGTTCGTACGATTGCAATGGGAACCTCTGACGGCCTG

TATCI_DSM13699{T} CAGCAGCAGCTGGGCGGTGGTGTTGTTCGTACGATTGCAATGGGTACTTCTGACGGCCTG

TATMO_LMG23360{T} CAGCAGCAGCTGGGCGGTGGTGTTGTTCGTACGATTGCAATGGGGACTTCTGACGGCCTG

BURGL_LMG2196{T} CAGCAGCAGCTCGGTGACGGCATCGTCCGCACCATCTGTTTGGGCGCCTCCGACGGCCTG

XANOR_ATCC35933{T} CAGCAGCAGCTCGGCGACGGCGTCGTGCGCACGATTGCGCTCGGCTCCACCGACGGCTTG

XANOR_CFBP2286{PT} CAGCAGCAGCTCGGCGACGGCGTCGTGCGCACGATTGCGCTCGGCTCCACCGACGGCTTG

PSEFU_LMG2158{T} CAGCAGCAGCTGGGCGACGGCGTGGTACGTACCATTGCGATGGGTTCCACCGAAGGCCTC

PSEOR_KCTC32247 CAGCAGCAGCTGGGCGACGGCGTGGTTCGTTCCATTGCGATGGGTTCCACCGAAGGCCTC

** ** * ** * ** ** * ** * * * * ** ** *

SPHME_DAPP-PG224{T} ACCCGCGGTCAGACCGTTCGCGACACCGGTTCGCAGATCAGCGTTCCCGTCGGTCCGGCG

SPHPA_NCTC11030{T} ACCCGCGGCCAGACCGTGACCGACACCGGCAACCAGATTCAGGTGCCGGTCGGCCCCGCG

PANAN_LMG2558{T} AAGCGTGGTCTGAACGTCAGCGACCTGCAGAAACCGATTCAGGTTCCAGTCGGTAAAGCG

PANBR_LMG5343{T} AAGCGTGGTCTGAGCGTCAACGACCTGCAGAAACCGATTCAGGTTCCGGTCGGTAAAGCG

PANCO_LMG24534{T} AAGCGTGGTCTGAGCGTCAACGACCTGCAGAAACCGATTCAGGTTCCGGTCGGTAAAGCG

PANDE_LMG24200{T} AAGCGTGGTCTGAGCGTCAACGACCTGCAGAAACCTATTCAGGTTCCCGTCGGTAAAGCG

PANVA_LMG24199{T} AAGCGTGGTCTGAACGTCAACGACCTGCAGAAACCTATTCAGGTTCCGGTCGGTAAAGCG

PANEU_LMG24197{T} AAGCGTGGTCTGAACGTCAACGACCTGCAGAAACCGATTCAGGTACCGGTCGGTAAAGCG

PANAG_DSM3493{T} AAGCGTGGTCTGAGCGTCAACGACCTGCAGAAACCGATTCAGGTACCCGTCGGTAAAGCG

PANAG_CFBP13505 AAGCGTGGTCTGAGCGTCAACGACCTGCAGAAACCGATTCAGGTACCCGTCGGTAAAGCG

MIXAL_LTYR-11Z{T} AAACGTGGCCTGGAAGTCGCTGATCTTAAAAAGCCGATTCAGGTACCGGTGGGTAAAGCT

MIXTH_QC88-366{T} AAACGTGGCCTGGAAGTCACCGATCTTAAAAAGCCGATTCAGGTACCGGTGGGTAAAGCT

MIXCA_DSM22759{T} AAGCGTGGCCTGGAAGTGACCGACCTTAAAAAGCCGATTCAGGTGCCGGTAGGTAAAGCT

MIXGA_DSM22758{T} AAGCGTGGTCTGGAAGTAACCGACCTTAAAAAGCCGATTCAGGTACCGGTAGGTAAAGCG

PANSE_LMG5345{T} AAGCGCGGTCTGGAAGTTGCTGACCTGAAAAAACCGATCCAGGTTCCGGTGGGTAAAGCC

PANEU_LMG5346{T} AAGCGCGGTCTGGAAGTAATCGATCTGAAAAAACCTATTCAGGTTCCGGTTGGTAAAGCG

PANDI_CCUG25232{T} AAGCGCGGTCTGGAAGTCACCGACCTGAAAAAACCTATCCAGGTTCCGGTTGGTAAAGCA

PANWA_LMG26277{T} AAGCGCGGTCTGGAAGTTGCTGACCTGAAAAAACCGATCCAGGTTCCGGTTGGTAAAGCT

PANRO_LMG26273{T} AAGCGCGGCCTCGAAGTCAACGACCTGAAAAAACCGATCCAGGTACCGGTTGGTAAACCA

PANRW_LMG26275{T} AAGCGCGGTCTGCAAGTAGCCGACCTGAAAAAACCGATCCAGGTACCGGTTGGTAAACCA

PANCY_LMG2657{T} AAGCGCGGTCTGGAAGTCACCGACCTGAAAAAACCGATCCAGGTACCGGTTGGTAAAGCA

PANST_CCUG26359{T} AAGCGCGGTCTGGAAGTTGCCGACCTGAAAAAACCGATCCAGGTACCGGTTGGTAAAGCA

PANST_LMG2632{PT} AAGCGCGGTCTGGAAGTTGCCGACCTGAAAAAACCGATCCAGGTACCGGTTGGTAAAGCA

PANAL_LMG24248{T} AAGCGCGGTCTGAACGTCACCGACCTGAAAAAACCGATCCAGGTACCGGTTGGTAAAGCA

PANAN_LMG2665{T} AAGCGCGGTCTGGAAGTCACCGACCTGAAAAAACCGATCCAGGTACCGGTTGGTAAAGCA

PANAN_RSA47 AAGCGCGGTCTGGAAGTCACCGACCTGAAAAAACCGATCCAGGTACCGGTTGGTAAAGCA

TATSA_NML06-3099{T} AAACGTGGCCTTAAGGTGAACGATCTGCAAAAACCAATTCAGGTACCCGTCGGTAAAGCG

TATPT_ATCC33301{T} AAACGTGGCCTTAAGGTGACCGATCTGCAAAAACCGATTCAGGTACCGGTCGGTAAAGCG

TATCI_DSM13699{T} AAGCGTGGCCTTAAGGTGACCGATCTGCAGAAACCTATCCAGGTTCCGGTCGGTAAAGCG

TATMO_LMG23360{T} AAGCGTGGCCTTAAGGTGACCGATCTGCAAAAACCGATTCAGGTTCCGGTCGGTAAAGCG

BURGL_LMG2196{T} CGCCGCGGCGTGCTGGTGAAGAACACCGGCAAGCCGATCTCGGTGCCGGTCGGCAAGCCG

XANOR_ATCC35933{T} AAGCGCAACCTGCTGGCCACCAACACCGAGCGCGCCATCTCGGTGCCGGTCGGTGCCGGT

XANOR_CFBP2286{PT} AAGCGCAACCTGCTGGCCACCAACACCGAGCGCGCCATCTCGGTGCCGGTCGGTGCCGGT

PSEFU_LMG2158{T} AAGCGTGGCCTGGACGTGAACAACACTGGCGCAGCCATCTCCGTACCAGTCGGTAAAGCG

PSEOR_KCTC32247 AAGCGTGGCCTGAGCGTGAGCAACACCGGCGCCGGCATCGCCGTGCCGGTCGGCAAGGCC

** * * ** ** ** ** **

SPHME_DAPP-PG224{T} ACGCTCGGCCGCATCATGAACGTCGTCGGCGAGCCGATCGATGAGCGTGGCCCGGTCGCC

SPHPA_NCTC11030{T} ACGCTCGGCCGCATCCTGAACGTCGTCGGCGAGCCGATCGACGAGCGTGGTCCGGTCGCC

PANAN_LMG2558{T} ACCCTGGGCCGTATCATGAACGTTCTCGGCGAGCCAATCGATATGAAAGGCGAGCTGAAA

PANBR_LMG5343{T} AC-CTGGGCCGTATCATGAACGTTCTCGGCGAGCCAATCGACATGAAAGGCGAGCTGAAA

PANCO_LMG24534{T} ACCCTGGGCCGTATCATGAACGTTCTCGGCGAGCCAATCGACATGAAAGGCGAGCTGAAA

PANDE_LMG24200{T} ACCCTGGGCCGTATCATGAACGTTCTCGGCGAGCCAATCGATATGAAAGGCGACCTGAAA

PANVA_LMG24199{T} ACCCTGGGCCGTATCATGAACGTTCTCGGCGAGCCAATCGACATGAAAGGCGAGCTGAAA

PANEU_LMG24197{T} ACTCTGGGCCGTATCATGAACGTTCTCGGCGAGCCAATCGATATGAAAGGCGAGCTGAAA

PANAG_DSM3493{T} ACCCTGGGCCGTATCATGAACGTTCTCGGCGAGCCAATCGATATGAAAGGCGAGCTGAAA

PANAG_CFBP13505 ACCCTGGGCCGTATCATGAACGTTCTCGGCGAGCCAATCGATATGAAAGGCGAGCTGAAA

MIXAL_LTYR-11Z{T} ACCCTGGGCCGTATCATGAACGTGCTTGGCGAGCCGATCGATATGAAAGGCGAACTGAAA

MIXTH_QC88-366{T} ACCCTGGGCCGTATCATGAACGTGCTTGGCGAGCCGATCGATATGAAAGGCGAGCTGAAA

MIXCA_DSM22759{T} ACCCTGGGCCGTATCATGAACGTGCTGGGTGAGCCGATCGACATGAAAGGCGAGCTGAAA

MIXGA_DSM22758{T} ACCCTGGGTCGTATCATGAACGTGCTGGGCGAGCCGATCGACATGAAAGGCGAGCTGAAG

PANSE_LMG5345{T} ACCCTCGGCCGTATCATGAACGTGCTGGGCGAGCCGATCGATATGAAAGGCGACATCAAA

PANEU_LMG5346{T} ACCCTTGGCCGTATCATGAACGTGCTGGGCGAGCCTATCGACATGAAAGGCGACCTGACA

PANDI_CCUG25232{T} ACACTCGGCCGTATCATGAACGTGCTGGGTGAGCCAATCGACATGAAAGGCGACCTGAAA

PANWA_LMG26277{T} ACCCTCGGCCGTATCATGAACGTATTGGGTGAGCCAATCGACATGAAGGGCGACCTGCAG

PANRO_LMG26273{T} ACCCTCGGCCGTATCATGAACGTATTGGGCGAACCAATCGACATGAAAGGCGACCTGCAG

PANRW_LMG26275{T} ACCCTCGGCCGTATCATGAACGTATTGGGTGAACCAATCGACATGAAAGGCGACCTGCAG

PANCY_LMG2657{T} ACCCTCGGCCGTATCATGAACGTGCTGGGCGAGCCGATCGACATGAAAGGCGACCTGAAA

PANST_CCUG26359{T} ACCCTCGGCCGTATCATGAACGTGCTGGGCGAGCCTATCGACATGAAAGGCGACCTGAAA

PANST_LMG2632{PT} ACCCTCGGCCGTATCATGAACGTGCTGGGCGAGCCTATCGACATGAAAGGCGACCTGAAA

PANAL_LMG24248{T} ACCCTCGGCCGTATCATGAACGTGCTGGGCGAGCCTATCGACATGAAAGGCGACCTGAAA

PANAN_LMG2665{T} ACCCTCGGCCGTATCATGAACGTGCTGGGCGAGCCTATCGACATGAAAGGCGACCTGACA

PANAN_RSA47 ACCCTCGGCCGTATCATGAACGTGCTGGGCGAGCCTATCGACATGAAAGGCGACCTGACA

TATSA_NML06-3099{T} ACCCTGGGCCGTATCATGAACGTCTTGGGTCAGCCAATCGATATGAAAGGCGACCTGCAG

TATPT_ATCC33301{T} ACGCTGGGCCGTATCATGAACGTACTGGGTCAGCCAATCGATATGAAAGGCGACCTGAAG

TATCI_DSM13699{T} ACTCTGGGTCGTATCATGAACGTTCTGGGTCAGCCAATCGATATGAAAGGTGAACTGCAG

TATMO_LMG23360{T} ACTCTGGGCCGTATCATGAACGTCCTGGGTCAGCCAATCGATATGAAAGGCGAACTGCAG

BURGL_LMG2196{T} ACCCTCGGCCGCATCATGGACGTGCTCGGCCGTCCGATCGACGAGGCCGGCCCGATCGAG

XANOR_ATCC35933{T} ACGCTGGGCCGCATCATGGACGTGCTGGGTCGTCCGATCGACGAAGCCGGCGACGTGCAG

XANOR_CFBP2286{PT} ACGCTGGGCCGCATCATGGACGTGCTGGGTCGTCCGATCGACGAAGCCGGCGACGTGCAG

PSEFU_LMG2158{T} ACCCTGGGCCGGATCATGGACGTGCTGGGCAACCCGATCGACGAAGCCGGCCCGATCGGC

PSEOR_KCTC32247 ACCCTGGGCCGCATCATGGACGTGCTGGGCAACCCGATCGACGAAGCGGGCCCCATCGGT

** ** ** ** *** ** **** * ** ** ***** ** *

**F3**

**GCAGTAGAGATCGCCTCTA**

SPHME_DAPP-PG224{T} ACCGAC-----CTGCGC----------GCGCCGATCCACGCGAAGGCGCCGGAATTCGTC

SPHPA_NCTC11030{T} ACCGGAC----CTGCGC----------GCCCCGATCCACGCCAAGGCGCCCGAGTTCATC

PANAN_LMG2558{T} GAAGAAGATGGCAGCGCAGTAGAGATCGCCTCTATTCACCGCGCGGCACCTTCTTATGAA

PANBR_LMG5343{T} GAAGAAGATGGCAGCGCAGTAGAGATCGCCTCTATTCACCGCGCGGCACCTTCTTATGAA

PANCO_LMG24534{T} GAAGAAGATGGTAGCGCAGTAGAGATCGCCTCTATTCACCGCGCGGCACCTTCTTATGAA

PANDE_LMG24200{T} GAAGAAGATGGCAGCGCAGTAGAGATCGCCTCTATTCACCGCGCAGCGCCTTCTTATGAA

PANVA_LMG24199{T} GAAGAAGACGGCAGTGCAGTAGAGATCGCCTCTATTCACCGCGCAGCGCCTTCTTATGAA

PANEU_LMG24197{T} GAAGAAGATGGCAGCGCAGTAGAGATCGCCTCTATTCACCGCGCAGCGCCTTCTTATGAA

PANAG_DSM3493{T} GAAGAAGATGGCAGCGCAGTAGAGATCGCCTCTATTCACCGCGCAGCCCCTTCTTATGAA

PANAG_CFBP13505 GAAGAAGATGGCAGCGCAGTAGAGATCGCCTCTATTCACCGCGCAGCCCCTTCTTATGAA

MIXAL_LTYR-11Z{T} GATGAAGACGGTGGTGCCGTAGAGATCGCCTCTATTCATCGCGCAGCGCCTTCATACGAA

MIXTH_QC88-366{T} GATGAAGACGGCAACGCAGTAGAGATCGCCTCTATTCACCGCGCTGCGCCTTCTTACGAA

MIXCA_DSM22759{T} GAAGAAGACGGCAGCGCAGTAGAGATCGCCTCTATTCACCGCGCGGCGCCTTCTTACGAA

MIXGA_DSM22758{T} GATGAAGACGGCGGCGTAGTAGAGATCGCCTCTATTCACCGTGCGGCACCTTCTTACGAA

PANSE_LMG5345{T} GAAGAAGACGGCAGTGCAGTAGAGATCTCCTCTATTCACCGCGCGGCGCCTTCTTATGAA

PANEU_LMG5346{T} GAAGAAGATGGCAGCGCAGTAGAGGTCTCCTCTATTCACCGCGCCGCGCCTTCCTATGAA

PANDI_CCUG25232{T} GAAGAAGACGGCAGCGCTGTAGAGGTTTCCTCTATTCATCGCGCAGCGCCTTCTTATGAA

PANWA_LMG26277{T} AATGACGACGGCACCACAGTAGAGGTCTCCTCTATTCACCGTGCTGCGCCTTCTTATGAA

PANRO_LMG26273{T} AATGAAGACGGCAGCGTAGTAGAGGTTTCCTCTATTCACCGTGCAGCCCCTTCATATGAA

PANRW_LMG26275{T} AATGAAGACGGCAGTGTTGTAGAGGTTTCCTCTATTCACCGTGCAGCGCCTTCTTATGAA

PANCY_LMG2657{T} GAAGAAGACGGCAGCGCAGTAGAGGTTTCCTCTATTCACCGTGCAGCACCTTCTTATGAA

PANST_CCUG26359{T} GAAGAAGATGGCAGTGCAGTAGAGGTTTCCTCTATTCACCGCCCTGCGCCTTCTTATGAA

PANST_LMG2632{PT} GAAGAAGATGGCAGTGCAGTAGAGGTTTCCTCTATTCACCGCCCTGCGCCTTCTTATGAA

PANAL_LMG24248{T} GAAGAAGACGGCAGCGTCGTAGAGGTTTCCTCTATTCACCGCCCTGCGCCTTCTTATGAA

PANAN_LMG2665{T} GAAGAAGACGGCAGCGCAGTAGAGGTTTCCTCTATTCACCGCCCTGCGCCTTCTTATGAA

PANAN_RSA47 GAAGAAGACGGCAGCGCAGTAGAGGTTTCCTCTATTCACCGCCCTGCGCCTTCTTATGAA

TATSA_NML06-3099{T} AACGAAGATGGTAGCACTGTTGAGGTGAACTCTATTCACCGTGCAGCGCCAAGCTATGAA

TATPT_ATCC33301{T} AACGAAGATGGTAGCAATGTTGAGGTGAACTCTATTCACCGTGCAGCGCCAAGCTACGAA

TATCI_DSM13699{T} AACGAAGATGGTAGCACTGTTGAGGTTAACTCCATTCACCGTGCAGCACCAAGCTACGAA

TATMO_LMG23360{T} AACGAAGATGGTAGCACTGTTGAGGTTAACTCAATCCACCGTGCCGCACCAAGCTACGAA

BURGL_LMG2196{T} AGCGAGCAT--CAGCGC-------------TCGATCCACCAGAAGGCGCCCGCGTTCGAC

XANOR_ATCC35933{T} GCGTCGGACCATTGGG---------------AAATCCATCGCGGCGCACCGTCGTACGAA

XANOR_CFBP2286{PT} GCGTCGGACCATTGGG---------------AAATCCATCGCAGCGCACCGTCGTACGAG

PSEFU_LMG2158{T} GAAGAAGAGCGCTGGG---------------GTATCCACCGCGCCGCTCCTTCCTTCGCA

PSEOR_KCTC32247 GAGGAAGAGCGCTGGA---------------CCATCCACCGCGCTGCCCCGTCCTATGCC

** ** ** ** *

**F2** **F-Loop** **F1c**

**GTCTAACTCGCAGGAACTG GGCATCAAGGTTATCGACCT TGTGT**

SPHME_DAPP-PG224{T} GACCAGTCGACCGACAGCGCGATCCTGGTCACCGGCATCAAGGTCATCGACCTGCTCGCG

SPHPA_NCTC11030{T} GAGCAGTCGACCGAGAGCGCGATTCTGGTCACCGGCATCAAGGTCATCGACCTTCTCGCG

PANAN_LMG2558{T} GATCAGTCTAACTCGCAGGAACTGCTGGAAACCGGCATCAAGGTTATCGACCTGATGTGT

PANBR_LMG5343{T} GATCAGTCTAACTCGCAGGAACTGCTGGAAACCGGCATCAAGGTTATCGACCTGATGTGT

PANCO_LMG24534{T} GATCAGTCTAACTCGCAGGAACTGCTGGAAACCGGCATCAAGGTTATCGACCTGATGTGT

PANDE_LMG24200{T} GAACAGTCTAACTCGCAGGAACTGCTGGAAACCGGCATCAAGGTTATCGACCTGATGTGT

PANVA_LMG24199{T} GATCAGTCTAACTCGCAGGAACTGCTGGAAACCGGCATCAAGGTTATCGACCTGATGTGT

PANEU_LMG24197{T} GATCAGTCTAACTCGCAGGAACTGCTGGAAACCGGCATCAAGGTTATCGACCTGATGTGT

PANAG_DSM3493{T} GATCAGTCTAACTCGCAGGAACTGCTGGAAACCGGCATCAAGGTTATCGACCTGATGTGT

PANAG_CFBP13505 GATCAGTCTAACTCGCAGGAACTGCTGGAAACCGGCATCAAGGTTATCGACCTGATGTGT

MIXAL_LTYR-11Z{T} GATCAGTCAAACTCGCAGGAACTGCTGGAAACCGGCATCAAGGTTATCGACCTGATGTGT

MIXTH_QC88-366{T} GATCAGTCAAACTCGCAGGAACTGCTGGAAACCGGCATCAAGGTTATCGACCTGATGTGT

MIXCA_DSM22759{T} GATCAGTCCAACTCCCAGGAACTGCTGGAAACCGGCATCAAGGTTATCGACCTGATGTGT

MIXGA_DSM22758{T} GATCAGTCAAACTCCCAGGAACTGCTGGAAACCGGCATCAAGGTTATCGACCTGATGTGT

PANSE_LMG5345{T} GACCAGTCTAACTCGCAGGAACTGCTGGAAACCGGCATCAAGGTTATCGACCTGATGTGT

PANEU_LMG5346{T} GATCAGTCAAACTCGCAGGAACTGCTGGAAACCGGCATCAAGGTTATCGACCTGATGTGT

PANDI_CCUG25232{T} GATCAGTCAAACTCGCAGGAACTGCTGGAAACCGGCATCAAGGTTATCGACCTGATGTGT

PANWA_LMG26277{T} GATCAGTCTAACTCGCAGGAACTGCTGGAGACCGGCATCAAGGTTATCGACCTGATGTGT

PANRO_LMG26273{T} GATCAGTCTAACTCGCAAGAGCTGCTGGAAACCGGCATCAAGGTTATCGACCTGATGTGT

PANRW_LMG26275{T} GATCAGTCTAACTCGCAAGAGCTGCTGGAAACCGGCATCAAGGTTATCGACCTGATGTGT

PANCY_LMG2657{T} GAACAGTCTAACTCGCAGGAACTGCTGGAAACCGGCATCAAGGTTATCGACCTGATGTGT

PANST_CCUG26359{T} GAGCAGTCTAACTCGCAGGAACTGCTGGAAACCGGCATCAAGGTTATCGACCTGATGTGT

PANST_LMG2632{PT} GAGCAGTCTAACTCGCAGGAACTGCTGGAAACCGGCATCAAGGTTATCGACCTGATGTGT

PANAL_LMG24248{T} GATCAGTCTAACTCGCAGGAACTGCTGGAAACCGGCATCAAGGTTATCGACCTGATGTGT

PANAN_LMG2665{T} GATCAGTCTAACTCGCAGGAACTGCTGGAAACCGGCATCAAGGTTATCGACCTGATGTGT

PANAN_RSA47 GATCAGTCTAACTCGCAGGAACTGCTGGAAACCGGCATCAAGGTTATCGACCTGATGTGT

TATSA_NML06-3099{T} GAACTGGCGAGCTCTACTGAACTGCTGGAAACTGGCATCAAGGTTATTGACCTGATCTGT

TATPT_ATCC33301{T} GAACTGGCTAACTCTACTGAGCTGCTGGAAACGGGTATCAAGGTTATCGACCTGATCTGT

TATCI_DSM13699{T} GAACTGGCTAACTCCACTGAACTGCTGGAAACCGGCATCAAGGTTATCGACCTGATCTGT

TATMO_LMG23360{T} GAACTGGCTAACTCCACTGAACTGCTGGAAACCGGCATCAAGGTTATCGACCTGATCTGT

BURGL_LMG2196{T} GAGCTGTCGCCGTCGACCGAGCTGCTCGAAACGGGCATCAAGGTGATCGACCTGGTCTGC

XANOR_ATCC35933{T} GACCAGTCCTCCAGCACCGAATTGCTGGAAACCGGCATCAAGGTCATCGACCTGATGTGC

XANOR_CFBP2286{PT} GACCAGTCCTCCAGCACCGAATTGCTGGAAACCGGCATCAAGGTCATCGACCTGATGTGC

PSEFU_LMG2158{T} GAACAGGCAGGCGGCAACGAACTGCTGGAAACCGGCATCAAGGTTATCGACCTGGTTTGC

PSEOR_KCTC32247 GAGCAGGCTGGCGGCAACGAGCTGCTGGAAACCGGCATCAAGGTGATCGACCTGGTCTGC

** * * * * * ** * ** ** ******** ** ***** *

**F1c** (cont’d) **B1c**

**CCGTTCGCCAAGG GTGGTGCGGGTGTAGGTAAA**

SPHME_DAPP-PG224{T} CCGTACGCGAAGGGCGGCAAGATCGGCCTGTTCGGCGGCGCCGGCGTCGGCAAGACCGTG

SPHPA_NCTC11030{T} CCGTACGCGAAGGGCGGCAAGATCGGCCTGTTCGGCGGCGCCGGCGTCGGCAAGACGGTT

PANAN_LMG2558{T} CCGTTTGCGAAAGGCGGTAAAGTCGGTCTGTTCGGTGGTGCGGGTGTGGGTAAAACCGTC

PANBR_LMG5343{T} CCGTTCGCCAAGGGCGGTAAAGTGGGTCTGTTCGGTGGCGCGGGTGTAGGTAAAACCGTC

PANCO_LMG24534{T} CCGTTCGCCAAGGGCGGTAAAGTCGGTCTGTTCGGTGGTGCGGGTGTGGGTAAAACCGTC

PANDE_LMG24200{T} CCGTTTGCCAAAGGCGGTAAAGTGGGTCTGTTCGGTGGTGCGGGTGTGGGTAAAACCGTC

PANVA_LMG24199{T} CCGTTTGCCAAAGGCGGTAAAGTGGGTCTGTTCGGTGGTGCGGGTGTCGGTAAAACCGTC

PANEU_LMG24197{T} CCGTTTGCCAAAGGCGGTAAAGTGGGTCTGTTCGGTGGTGCGGGTGTAGGTAAAACCGTC

PANAG_DSM3493{T} CCGTTTGCTAAAGGCGGTAAAGTCGGTCTGTTCGGTGGTGCGGGTGTAGGTAAAACCGTC

PANAG_CFBP13505 CCGTTTGCTAAAGGCGGTAAAGTCGGTCTGTTCGGTGGTGCGGGTGTAGGTAAAACCGTC

MIXAL_LTYR-11Z{T} CCGTTCGCTAAGGGCGGTAAAGTGGGTCTGTTCGGTGGTGCGGGCGTAGGTAAAACCGTA

MIXTH_QC88-366{T} CCGTTCGCTAAAGGCGGTAAAGTCGGTCTGTTCGGTGGTGCGGGCGTAGGTAAAACCGTA

MIXCA_DSM22759{T} CCGTTCGCCAAGGGCGGTAAAGTCGGTCTGTTCGGCGGCGCGGGCGTTGGTAAAACCGTA

MIXGA_DSM22758{T} CCGTTCGCCAAGGGCGGTAAAGTCGGCCTGTTCGGCGGCGCGGGCGTAGGTAAAACCGTA

PANSE_LMG5345{T} CCGTTTGCCAAGGGCGGTAAAGTCGGCCTGTTCGGCGGCGCGGGCGTAGGTAAAACCGTA

PANEU_LMG5346{T} CCGTTCGCTAAGGGCGGTAAAGTCGGTCTGTTCGGTGGTGCGGGCGTAGGTAAAACCGTA

PANDI_CCUG25232{T} CCGTTCGCGAAGGGCGGTAAAGTCGGTCTGTTCGGTGGTGCGGGTGTAGGTAAAACCGTA

PANWA_LMG26277{T} CCGTTCGCTAAGGGCGGTAAAGTTGGTCTGTTCGGTGGTGCGGGTGTAGGTAAAACCGTA

PANRO_LMG26273{T} CCGTTCGCTAAGGGCGGTAAAGTCGGTTTGTTCGGTGGTGCGGGTGTAGGTAAAACCGTA

PANRW_LMG26275{T} CCGTTCGCTAAGGGCGGTAAAGTCGGTCTGTTCGGTGGTGCGGGTGTAGGTAAAACCGTA

PANCY_LMG2657{T} CCGTTCGCTAAGGGCGGTAAAGTCGGTCTGTTCGGTGGTGCGGGTGTAGGTAAAACCGTA

PANST_CCUG26359{T} CCGTTCGCGAAGGGCGGTAAAGTCGGTCTGTTCGGTGGTGCGGGTGTGGGTAAAACCGTA

PANST_LMG2632{PT} CCGTTCGCGAAGGGCGGTAAAGTCGGTCTGTTCGGTGGTGCGGGTGTGGGTAAAACCGTA

PANAL_LMG24248{T} CCGTTCGCTAAGGGCGGTAAAGTCGGTCTGTTCGGTGGTGCGGGTGTAGGTAAAACCGTA

PANAN_LMG2665{T} CCGTTCGCTAAGGGCGGTAAAGTCGGTCTGTTCGGTGGTGCGGGTGTAGGTAAAACCGTA

PANAN_RSA47 CCGTTCGCTAAGGGCGGTAAAGTCGGTCTGTTCGGTGGTGCGGGTGTAGGTAAAACCGTA

TATSA_NML06-3099{T} CCGTTTGCGAAAGGTGGTAAAGTAGGTCTGTTCGGTGGTGCGGGTGTTGGTAAGACCGTT

TATPT_ATCC33301{T} CCGTTTGCAAAAGGCGGTAAAGTGGGTCTGTTCGGTGGTGCGGGTGTAGGTAAGACCGTC

TATCI_DSM13699{T} CCGTTTGCTAAAGGTGGTAAAGTTGGTCTGTTCGGTGGTGCGGGTGTTGGTAAGACCGTA

TATMO_LMG23360{T} CCGTTTGCTAAAGGTGGTAAAGTGGGTCTGTTCGGTGGTGCGGGTGTTGGTAAGACCGTA

BURGL_LMG2196{T} CCGTTCGCGAAGGGCGGCAAGGTCGGCCTGTTCGGCGGCGCCGGCGTCGGCAAGACCGTC

XANOR_ATCC35933{T} CCGTTCGCCAAGGGCGGCAAGGTCGGCCTGTTCGGCGGCGCCGGCGTCGGCAAGACCGTC

XANOR_CFBP2286{PT} CCGTTCGCCAAGGGCGGCAAGGTCGGCCTGTTCGGCGGCGCCGGCGTCGGCAAGACCGTC

PSEFU_LMG2158{T} CCGTTCGCCAAGGGTGGTAAGGTCGGTCTGTTCGGTGGTGCCGGTGTAGGCAAAACCGTA

PSEOR_KCTC32247 CCGTTCGCCAAGGGCGGCAAGGTCGGCCTGTTCGGCGGCGCCGGCGTGGGCAAGACCGTG

**** ** ** ** ** ** * ** ******* ** ** ** ** ** ** ** **

**B-Loop** **B2**

**AACATGATGGAACTGATCCGT CTGAACACTCAGGTTACTCG**

SPHME_DAPP-PG224{T} CTGATCCAGGAACTGATCAACAACATCGCCAAGGGCCACGGCGGCACCTCGGTGTTCGCG

SPHPA_NCTC11030{T} CTGATTCAGGAACTCATCAATAACATCGCGAAGGGGCACGGCGGCACCTCGGTCTTCGCG

PANAN_LMG2558{T} AACATGATGGAACTGATCCGTAACATCGCGGCTGAGCACTCAGGTTACTCTGTGTTTGCC

PANBR_LMG5343{T} AACATGATGGAACTGATCCGTAACATCGCGGCTGAACACTCAGGTTACTCAGTGTTTGCC

PANCO_LMG24534{T} AACATGATGGAGCTGATCCGTAACATCGCGGCTGAACACTCAGGTTACTCAGTGTTTGCC

PANDE_LMG24200{T} AACATGATGGAACTGATCCGTAACATCGCGGCTGAACACTCAGGTTACTCTGTGTTTGCC

PANVA_LMG24199{T} AACATGATGGAACTGATCCGTAACATCGCGGCTGAACACTCAGGTTACTCTGTGTTTGCC

PANEU_LMG24197{T} AACATGATGGAACTGATCCGTAACATCGCAGCTGAACACTCAGGTTACTCAGTGTTTGCT

PANAG_DSM3493{T} AACATGATGGAACTGATCCGTAACATCGCGGCTGAACACTCAGGTTACTCAGTGTTTGCC

PANAG_CFBP13505 AACATGATGGAACTGATCCGTAACATCGCGGCTGAACACTCAGGTTACTCAGTGTTTGCC

MIXAL_LTYR-11Z{T} AACATGATGGAGCTGATCCGTAACATCGCGGCTGAGCACTCAGGTTACTCGGTATTTGCC

MIXTH_QC88-366{T} AACATGATGGAGCTGATCCGTAACATCGCGGCTGAGCACTCAGGTTACTCGGTATTTGCC

MIXCA_DSM22759{T} AACATGATGGAGCTGATCCGTAACATCGCGGCTGAGCACTCAGGTTACTCGGTATTCGCC

MIXGA_DSM22758{T} AACATGATGGAGCTTATCCGTAACATCGCGGCTGAGCATTCAGGTTACTCGGTATTTGCC

PANSE_LMG5345{T} AACATGATGGAGCTGATCCGTAACATCGCGGCTGAGCACTCAGGTTACTCGGTATTTGCC

PANEU_LMG5346{T} AACATGATGGAGCTGATCCGTAACATCGCGGCTGAGCACTCAGGTTATTCGGTCTTTGCT

PANDI_CCUG25232{T} AACATGATGGAGCTGATCCGTAACATCGCGGCTGAGCACTCAGGTTATTCGGTCTTTGCC

PANWA_LMG26277{T} AACATGATGGAGCTGATCCGTAACATTGCGGCTGAGCACTCAGGTTACTCGGTATTTGCC

PANRO_LMG26273{T} AACATGATGGAGCTGATCCGTAACATCGCGGCTGAGCACTCAGGTTATTCGGTCTTTGCT

PANRW_LMG26275{T} AACATGATGGAGCTGATCCGTAACATCGCGGCTGAGCACTCAGGTTATTCGGTCTTTGCT

PANCY_LMG2657{T} AACATGATGGAGCTGATCCGTAACATCGCGGCTGAGCACTCAGGTTACTCGGTATTTGCC

PANST_CCUG26359{T} AACATGATGGAGCTGATCCGTAACATTGCGGCTGAGCACTCAGGTTACTCGGTATTTGCC

PANST_LMG2632{PT} AACATGATGGAGCTGATCCGTAACATTGCGGCTGAGCACTCAGGTTACTCGGTATTTGCC

PANAL_LMG24248{T} AACATGATGGAGCTTATCCGTAACATCGCGGCTGAGCACTCAGGTTATTCGGTCTTTGCT

PANAN_LMG2665{T} AACATGATGGAGCTGATCCGTAACATTGCGGCTGAGCACTCAGGTTACTCGGTATTTGCC

PANAN_RSA47 AACATGATGGAGCTGATCCGTAACATTGCGGCTGAGCACTCAGGTTACTCGGTATTTGCC

TATSA_NML06-3099{T} AACATGATGGAGCTTATCCGTAACATCGCTATCGAACACTCTGGTTACTCAGTATTCGCC

TATPT_ATCC33301{T} AACATGATGGAACTGATCCGTAACATCGCTATCGAGCACTCTGGTTACTCTGTATTTGCA

TATCI_DSM13699{T} AACATGATGGAACTGATCCGTAACATCGCGATCGAGCACTCAGGTTACTCTGTATTCGCA

TATMO_LMG23360{T} AACATGATGGAACTGATCCGTAACATCGCGATCGAACACTCCGGTTACTCTGTATTCGCA

BURGL_LMG2196{T} AACATGATGGAGCTGATCAACAACATCGCGAAGGAGCACGGCGGCTACTCCGTGTTCGCG

XANOR_ATCC35933{T} AACATGATGGAACTGATCAACAACATCGCCAAGGCGCACAGCGGCTTGTCCGTGTTCGCC

XANOR_CFBP2286{PT} AACATGATGGAACTGATCAACAACATCGCCAAGGCGCACAGCGGCTTGTCCGTGTTCGCC

PSEFU_LMG2158{T} AACATGATGGAACTGATCCGTAACATCGCCATCGAGCACAGCGGTTATTCCGTGTTCGCC

PSEOR_KCTC32247 AACATGATGGAGCTGATCCGCAACATCGCCATGGAGCACAGCGGTTACTCCGTGTTCGCC

** *** ** *** ***** ** * ** ** ** ** ** **

**B3**

**GACCGACTCCAAC**

SPHME_DAPP-PG224{T} GGCGTCGGCGAGCGCACCCGCGAGGGCAACGACCTCTACCACGAATTCCTCGACGCGGGC

SPHPA_NCTC11030{T} GGCGTCGGTGAGCGTACCCGCGAGGGCAACGACCTGTATCACGAATTCCTCGACGCGGGC

PANAN_LMG2558{T} GGTGTGGGTGAGCGTACTCGTGAGGGTAACGACTTCTACCACGAAATGACCGACTCTAAC

PANBR_LMG5343{T} GGTGTGGGTGAGCGTACTCGTGAGGGTAACGACTTCTACCACGAAATGACCGACTCCAAC

PANCO_LMG24534{T} GGTGTGGGTGAGCGTACTCGTGAGGGTAACGACTTCTACCACGAAATGACTGACTCTAAC

PANDE_LMG24200{T} GGTGTGGGTGAGCGTACTCGTGAGGGTAACGACTTCTACCACGAAATGACCGACTCAAAC

PANVA_LMG24199{T} GGTGTGGGTGAGCGTACTCGTGAGGGTAACGACTTCTACCACGAAATGACTGACTCAAAC

PANEU_LMG24197{T} GGTGTGGGTGAGCGTACTCGTGAGGGTAACGACTTCTACCACGAAATGACTGACTCAAAC

PANAG_DSM3493{T} GGTGTGGGTGAGCGTACTCGTGAGGGTAACGACTTCTACCACGAAATGACTGACTCTAAC

PANAG_CFBP13505 GGTGTGGGTGAGCGTACTCGTGAGGGTAACGACTTCTACCACGAAATGACTGACTCTAAC

MIXAL_LTYR-11Z{T} GGTGTGGGCGAGCGTACTCGTGAGGGTAACGACTTCTACCACGAAATGACCGACTCTAAC

MIXTH_QC88-366{T} GGCGTGGGTGAGCGTACTCGTGAGGGTAACGACTTCTACCACGAAATGACCGACTCTAAC

MIXCA_DSM22759{T} GGTGTGGGCGAGCGTACTCGTGAGGGTAACGACTTCTACCACGAAATGACCGACTCCAAC

MIXGA_DSM22758{T} GGCGTCGGTGAGCGTACGCGTGAGGGTAACGACTTCTACCACGAAATGACCGACTCCAAC

PANSE_LMG5345{T} GGCGTGGGCGAGCGTACTCGTGAGGGTAACGACTTCTACCACGAAATGACCGACTCCAAC

PANEU_LMG5346{T} GGTGTTGGTGAGCGTACTCGTGAGGGTAACGACTTCTATCACGAAATGACTGACTCCAAC

PANDI_CCUG25232{T} GGCGTGGGTGAGCGTACTCGTGAGGGTAACGACTTCTACCACGAAATGACGGACTCCAAC

PANWA_LMG26277{T} GGCGTGGGTGAGCGTACTCGTGAGGGTAACGACTTCTACCACGAAATGACTGACTCCAAC

PANRO_LMG26273{T} GGTGTAGGTGAGCGTACTCGTGAGGGTAACGACTTCTACCACGAAATGACTGACTCCAAC

PANRW_LMG26275{T} GGCGTGGGTGAACGTACTCGTGAGGGTAACGACTTCTACCACGAAATGACTGACTCCAAC

PANCY_LMG2657{T} GGTGTGGGTGAGCGTACTCGTGAGGGTAACGACTTCTACCACGAAATGACTGACTCCAAC

PANST_CCUG26359{T} GGCGTGGGTGAGCGTACTCGTGAGGGTAACGACTTCTACCACGAAATGACTGACTCCAAC

PANST_LMG2632{PT} GGCGTAGGTGAGCGTACTCGTGAGGGTAACGACTTCTACCACGAAATGACTGACTCCAAC

PANAL_LMG24248{T} GGCGTGGGTGAACGTACTCGTGAGGGTAACGACTTCTACCACGAAATGACTGACTCCAAC

PANAN_LMG2665{T} GGCGTGGGTGAGCGTACTCGTGAGGGTAACGACTTCTACCACGAAATGACTGACTCCAAC

PANAN_RSA47 GGCGTGGGTGAGCGTACTCGTGAGGGTAACGACTTCTACCACGAAATGACTGACTCCAAC

TATSA_NML06-3099{T} GGTGTAGGTGAGCGTACCCGTGAAGGTAACGACTTCTACCACGAAATGACCGAATCTAAC

TATPT_ATCC33301{T} GGGGTGGGTGAGCGTACCCGTGAAGGTAACGACTTCTACCACGAAATGACCGAGTCTAAC

TATCI_DSM13699{T} GGGGTCGGTGAGCGTACTCGTGAAGGTAACGACTTCTATCATGAAATGACCGAGTCTAAC

TATMO_LMG23360{T} GGGGTCGGTGAGCGTACTCGTGAAGGTAACGACTTCTATCATGAAATGACCGAGTCTAAC

BURGL_LMG2196{T} GGCGTGGGCGAGCGGACCCGTGAAGGGAACGACTTCTACCACGAAATGAAGGACTCCAAC

XANOR_ATCC35933{T} GGCGTGGGCGAGCGTACCCGCGAAGGCAACGACTTCTACCACGAGATGAAGGACTCCAAC

XANOR_CFBP2286{PT} GGCGTGGGCGAGCGTACCCGCGAAGGCAACGACTTCTACCACGAGATGAAGGACTCCAAC

PSEFU_LMG2158{T} GGTGTGGGTGAGCGTACTCGTGAGGGTAACGACTTCTACCACGAGATGAAGGACTCCAAC

PSEOR_KCTC32247 GGCGTGGGTGAGCGTACTCGTGAAGGTAACGACTTCTATCACGAGATGAAGGACTCCAAC

** ** ** ** ** ** ** ** ** ****** * ** ** ** * ** * *

**B3** (cont’d)

**GTTATC**

SPHME_DAPP-PG224{T} GTCATCGCCAAGGATGCCGAGGGCAATCCGATCAGCGAGGGATCGAAGGTCGCGCTGGTG

SPHPA_NCTC11030{T} GTGATCGCCAAGGACGCCGAGGGCAACGCGATCAGCGAGGGCTCGAAGGTCGCGCTGGTC

PANAN_LMG2558{T} GTTATCGAT------------------------------------AAAGTTGCGCTGGTC

PANBR_LMG5343{T} GTTATCGAC------------------------------------AAAGTTGCCCTGGTG

PANCO_LMG24534{T} GTTATCGAT------------------------------------AAAGTTGCGCTGGTC

PANDE_LMG24200{T} GTTATCGAT------------------------------------AAAGTTGCCCTGGTC

PANVA_LMG24199{T} GTTATCGAT------------------------------------AAAGTTGCGCTGGTC

PANEU_LMG24197{T} GTTATCGAC------------------------------------AAAGTTGCTCTGGTC

PANAG_DSM3493{T} GTTATCGAT------------------------------------AAAGTTGCACTGGTC

PANAG_CFBP13505 GTTATCGAT------------------------------------AAAGTTGCACTGGTC

MIXAL_LTYR-11Z{T} GTTATCGAT------------------------------------AAAGTTGCGCTGGTT

MIXTH_QC88-366{T} GTAATCGAC------------------------------------AAAGTTGCGCTGGTT

MIXCA_DSM22759{T} GTAATCGAC------------------------------------AAAGTTGCGCTGGTC

MIXGA_DSM22758{T} GTAATCGAC------------------------------------AAAGTTGCGCTGGTA

PANSE_LMG5345{T} GTAATCGAC------------------------------------AAAGTTGCGCTGGTG

PANEU_LMG5346{T} GTTATCGAT------------------------------------AAAGTAGCGCTGGTG

PANDI_CCUG25232{T} GTTATCGAT------------------------------------AAAGTAGCGCTGGTG

PANWA_LMG26277{T} GTTATCGAT------------------------------------AAAGTAGCGCTGGTG

PANRO_LMG26273{T} GTTATCGAT------------------------------------AAAGTAGCGCTGGTG

PANRW_LMG26275{T} GTTATCGAT------------------------------------AAAGTAGCGCTGGTG

PANCY_LMG2657{T} GTTATCGAT------------------------------------AAAGTAGCGCTGGTG

PANST_CCUG26359{T} GTTATCGAC------------------------------------AAAGTTGCGCTGGTG

PANST_LMG2632{PT} GTTATCGAC------------------------------------AAAGTTGCGCTGGTG

PANAL_LMG24248{T} GTTATCGAT------------------------------------AAAGTCGCGCTGGTG

PANAN_LMG2665{T} GTTATCGAT------------------------------------AAAGTTGCGCTGGTG

PANAN_RSA47 GTTATCGAT------------------------------------AAAGTCGCGCTGGTG

TATSA_NML06-3099{T} GTTCTGGAT------------------------------------AAAGTTGCACTGGTT

TATPT_ATCC33301{T} GTTCTGGAT------------------------------------AAAGTTGCTCTGGTT

TATCI_DSM13699{T} GTTCTGGAT------------------------------------AAAGTTGCTCTGGTT

TATMO_LMG23360{T} GTTCTGGAT------------------------------------AAAGTTGCTCTGGTT

BURGL_LMG2196{T} GTGCTCGAC------------------------------------AAGGTCGCGCTGGTG

XANOR_ATCC35933{T} GTTCTGGAC------------------------------------AAGGTCGCGATGGTG

XANOR_CFBP2286{PT} GTTCTGGAC------------------------------------AAGGTCGCGATGGTG

PSEFU_LMG2158{T} GTTCTGGAC------------------------------------AAAGTGGCACTGGTC

PSEOR_KCTC32247 GTTCTGGAC------------------------------------AAGGTCGCCCTGGTC

** * * ** ** ** ****

SPHME_DAPP-PG224{T} TTCGGCCAGATGAACGAGCCGCCGGGCGCCCGCGCCCGCGTCGCGCTGTCGGGCCTGACC

SPHPA_NCTC11030{T} TATGGCCAGATGAACGAGCCGCCGGGCGCCCGTGCGCGCGTCGCGCTGTCGGGTCTCGCT

PANAN_LMG2558{T} TATGGCCAGATGAACGAGCCGCCGGGTAACCGTCTGCGCGTCGCACTGACCGGTCTGACC

PANBR_LMG5343{T} TATGGCCAGATGAACGAGCCGCCGGGTAACCGTCTGCGCGTGGCACTGACCGGTCTGACC

PANCO_LMG24534{T} TATGGCCAGATGAACGAGCCGCCGGGTAACCGTCTGCGCGTAGCACTGACCGGTCTGACC

PANDE_LMG24200{T} TATGGCCAGATGAACGAGCCGCCGGGTAACCGTCTGCGCGTGGCACTGACCGGTCTGACC

PANVA_LMG24199{T} TATGGCCAGATGAACGAGCCGCCGGGTAACCGTCTGCGCGTTGCACTGACCGGTCTGACC

PANEU_LMG24197{T} TATGGCCAGATGAACGAGCCGCCGGGTAACCGTCTGCGCGTAGCCCTGACCGGTCTGACC

PANAG_DSM3493{T} TATGGCCAGATGAACGAGCCGCCGGGTAACCGTCTGCGCGTAGCACTGACCGGTCTGACC

PANAG_CFBP13505 TATGGCCAGATGAACGAGCCGCCGGGTAACCGTCTGCGCGTAGCACTGACCGGTCTGACC

MIXAL_LTYR-11Z{T} TATGGCCAGATGAACGAGCCGCCGGGCAACCGTCTGCGTGTAGCGCTGACCGGTCTGACC

MIXTH_QC88-366{T} TATGGCCAGATGAACGAGCCGCCGGGTAACCGTCTGCGCGTAGCACTGACCGGTCTGACC

MIXCA_DSM22759{T} TACGGCCAGATGAACGAGCCGCCGGGCAACCGTCTGCGCGTGGCGCTGACCGGCCTGACC

MIXGA_DSM22758{T} TACGGCCAGATGAACGAGCCGCCGGGCAACCGTCTGCGCGTGGCACTGACCGGTCTGACC

PANSE_LMG5345{T} TATGGCCAGATGAACGAGCCGCCGGGTAACCGTCTGCGCGTGGCACTGACCGGTCTGACC

PANEU_LMG5346{T} TATGGCCAGATGAACGAGCCGCCGGGTAACCGTCTGCGTGTAGCACTGACCGGTCTGACC

PANDI_CCUG25232{T} TATGGCCAGATGAACGAGCCGCCGGGTAACCGTCTGCGCGTAGCACTGACCGGTCTGACC

PANWA_LMG26277{T} TATGGCCAGATGAACGAGCCGCCGGGTAACCGTCTGCGCGTAGCACTGACCGGTCTGACC

PANRO_LMG26273{T} TATGGCCAGATGAACGAGCCGCCGGGTAACCGTCTGCGCGTAGCACTGACCGGTCTGACC

PANRW_LMG26275{T} TATGGCCAGATGAACGAGCCGCCGGGTAACCGTCTGCGCGTAGCACTGACCGGTCTGACC

PANCY_LMG2657{T} TATGGCCAGATGAACGAGCCGCCGGGTAACCGTCTGCGCGTCGCACTGACCGGTCTGACC

PANST_CCUG26359{T} TATGGCCAGATGAACGAGCCGCCGGGTAACCGTCTGCGCGTTGCACTGACCGGTCTGACC

PANST_LMG2632{PT} TATGGCCAGATGAACGAGCCGCCGGGTAACCGTCTGCGCGTTGCACTGACCGGTCTGACC

PANAL_LMG24248{T} TATGGCCAGATGAACGAGCCGCCGGGTAACCGTCTGCGCGTGGCGCTGACCGGTCTGACC

PANAN_LMG2665{T} TATGGCCAGATGAACGAGCCGCCGGGTAACCGTCTGCGCGTGGCACTGACCGGTCTGACC

PANAN_RSA47 TATGGCCAGATGAACGAGCCGCCGGGTAACCGTCTGCGCGTGGCGCTGACCGGTCTGACC

TATSA_NML06-3099{T} TATGGCCAGATGAACGAGCCACCAGGGAACCGTCTGCGTGTTGCGCTGACCGGTCTGACC

TATPT_ATCC33301{T} TATGGCCAGATGAACGAGCCACCAGGAAACCGTCTGCGCGTTGCGCTGACCGGTCTGACT

TATCI_DSM13699{T} TATGGCCAGATGAACGAGCCACCAGGAAACCGTCTGCGTGTTGCGCTGACTGGTCTGACT

TATMO_LMG23360{T} TATGGCCAGATGAACGAGCCACCAGGAAACCGTCTGCGTGTTGCGCTGACTGGACTGACT

BURGL_LMG2196{T} TACGGCCAGATGAACGAGCCGCCGGGCAACCGTCTGCGCGTCGCGCTGACCGGCCTGACG

XANOR_ATCC35933{T} TACGGCCAGATGAACGAGCCGCCGGGCAACCGTCTGCGCGTTGCGCTGACCGGCCTGACC

XANOR_CFBP2286{PT} TACGGCCAGATGAACGAGCCGCCGGGCAACCGTCTGCGCGTTGCGCTGACCGGCCTGACC

PSEFU_LMG2158{T} TACGGCCAGATGAACGAGCCGCCGGGAAACCGTCTGCGCGTAGCCCTGACCGGCCTGACC

PSEOR_KCTC32247 TACGGCCAGATGAACGAGCCGCCGGGCAACCGTCTGCGCGTCGCCCTGACCGGCCTGACC

* ***************** ** ** *** ** ** ** *** * ** ** *

SPHME_DAPP-PG224{T} ATCGCGGAATATTTCCGCGATCAGG---------------AAGGACAGGACGTGCTGTTC

SPHPA_NCTC11030{T} ATCGCGGAATATTTCCGCGACCAGG---------------AAGGCCAGGACGTGCTGTTC

PANAN_LMG2558{T} ATGGCGGAAAAATTCCGTGATG------------------AAGGCCGCGACGTTCTGCTG

PANBR_LMG5343{T} ATGGCGGAAAAATTCCGTGATG------------------AAGGCCGCGACGTTCTGCTG

PANCO_LMG24534{T} ATGGCGGAAAAATTCCGTGATG------------------AAGGCCGTGACGTTCTGCTG

PANDE_LMG24200{T} ATGGCGGAAAAATTCCGTGATG------------------AAGGCCGCGACGTTCTGCTG

PANVA_LMG24199{T} ATGGCGGAAAAATTCCGTGATG------------------AAGGCCGCGACGTTCTGCTG

PANEU_LMG24197{T} ATGGCGGAAAAATTCCGTGATG------------------AAGGCCGCGACGTTCTGCTG

PANAG_DSM3493{T} ATGGCGGAAAAATTCCGTGATG------------------AAGGTCGCGACGTTCTGCTG

PANAG_CFBP13505 ATGGCGGAAAAATTCCGTGATG------------------AAGGCCGCGACGTTCTGCTG

MIXAL_LTYR-11Z{T} ATGGCGGAGAAATTCCGTGATG------------------AAGGCCGTGACGTTCTGCTG

MIXTH_QC88-366{T} ATGGCGGAGAAATTCCGTGACG------------------AAGGCCGCGACGTTCTGCTG

MIXCA_DSM22759{T} ATGGCGGAAAAATTCCGTGATG------------------AAGGCCGCGACGTACTGCTG

MIXGA_DSM22758{T} ATGGCGGAAAAATTCCGTGATG------------------AAGGCCGCGACGTACTGCTG

PANSE_LMG5345{T} ATGGCGGAAAAATTCCGTGACG------------------AAGGCCGCGATGTTCTGCTG

PANEU_LMG5346{T} ATGGCGGAAAAATTCCGTGATG------------------AAGGTCGTGACGTTCTGCTG

PANDI_CCUG25232{T} ATGGCGGAAAAATTCCGTGATG------------------AAGGCCGTGACGTTCTGCTG

PANWA_LMG26277{T} ATGGCGGAAAAATTCCGTGATG------------------AAGGCCGTGACGTTCTGCTG

PANRO_LMG26273{T} ATGGCGGAAAAATTCCGTGATG------------------AAGGCCGTGACGTTCTGCTG

PANRW_LMG26275{T} ATGGCGGAGAAATTCCGTGATG------------------AAGGCCGTGACGTTCTGCTG

PANCY_LMG2657{T} ATGGCGGAAAAATTCCGTGATG------------------AAGGCCGTGACGTACTGCTG

PANST_CCUG26359{T} ATGGCGGAGAAATTCCGTGATG------------------AAGGCCGTGACGTTCTGCTG

PANST_LMG2632{PT} ATGGCGGAGAAATTCCGTGATG------------------AAGGCCGTGACGTTCTGCTG

PANAL_LMG24248{T} ATGGCGGAGAAATTCCGTGATG------------------AAGGCCGTGACGTTCTGCTG

PANAN_LMG2665{T} ATGGCGGAGAAATTCCGTGATG------------------AAGGCCGCGACGTTCTGCTG

PANAN_RSA47 ATGGCGGAGAAATTCCGTGATG------------------AAGGCCGTGACGTTCTGCTG

TATSA_NML06-3099{T} ATGGCTGAAAAATTCCGTGACG------------------AAGGTCGTGACGTATTGCTG

TATPT_ATCC33301{T} ATGGCTGAAAAATTCCGTGACG------------------AAGGCCGTGACGTACTGCTG

TATCI_DSM13699{T} ATGGCTGAGAAATTCCGTGACG------------------AAGGCCGTGATGTTCTGCTG

TATMO_LMG23360{T} ATGGCTGAGAAATTCCGTGACG------------------AAGGCCGTGATGTTCTGCTG

BURGL_LMG2196{T} ATGGCCGAGCACTTCCGTGACG------------------AAGGCCTCGACGTGCTGTTC

XANOR_ATCC35933{T} ATGGCCGAGTACTTCCGCGACGAGAAGGACGCCAACGGCAAGGGCAAGGACGTGCTGCTG

XANOR_CFBP2286{PT} ATGGCCGAGTACTTCCGCGACGAGAAGGACGCCAACGGCAAGGGCAAGGACGTGCTGCTG

PSEFU_LMG2158{T} ATGGCCGAGAAGTTCCGTGACG------------------AAGGTAACGACGTTCTGCTG

PSEOR_KCTC32247 ATGGCCGAGAAGTTCCGTGACG------------------AAGGCCGCGACGTTCTGCTG

** ** ** * ***** ** * ** ** ** ** *

SPHME_DAPP-PG224{T} TTCGTCGACAACATCTTCCGCTTCACGCAGGCGGGCGCGGAGGTGTCGGCACTGCTCGGC

SPHPA_NCTC11030{T} TTCGTCGACAACATCTTCCGCTTCACCCAGGCGGGTGCGGAAGTGTCGGCGCTGCTGGGC

PANAN_LMG2558{T} TTCATCGATAACATCTACCGTTATACACTGGCCGGTACAGAAGTTTCTGCACTGCTGGGT

PANBR_LMG5343{T} TTCATCGATAACATCTACCGTTATACCCTGGCCGGTACCGAAGTTTCTGCACTGCTGGGT

PANCO_LMG24534{T} TTCATCGATAACATCTACCGTTACACCCTGGCCGGTACCGAAGTGTCGGCACTGCTGGGT

PANDE_LMG24200{T} TTCATCGATAACATCTACCGTTACACCCTGGCCGGTACAGAAGTCTCTGCACTGCTGGGT

PANVA_LMG24199{T} TTCATCGATAACATCTACCGTTATACCCTGGCCGGTACAGAAGTTTCTGCACTGCTGGGT

PANEU_LMG24197{T} TTCATCGATAACATCTACCGTTATACCCTGGCCGGTACAGAAGTCTCTGCACTGCTGGGT

PANAG_DSM3493{T} TTCATCGATAACATCTACCGTTATACCCTGGCCGGTACAGAAGTTTCTGCACTGCTGGGT

PANAG_CFBP13505 TTCATCGATAACATCTACCGTTATACCCTGGCCGGTACAGAAGTTTCTGCACTGCTGGGT

MIXAL_LTYR-11Z{T} TTTATCGATAACATCTACCGTTACACCCTGGCCGGTACTGAAGTATCCGCGCTGCTGGGT

MIXTH_QC88-366{T} TTTATCGATAACATCTACCGTTACACCCTGGCCGGTACCGAAGTGTCCGCGCTGCTGGGT

MIXCA_DSM22759{T} TTCATCGATAACATCTACCGTTACACCCTGGCCGGTACCGAAGTCTCCGCGCTGCTGGGT

MIXGA_DSM22758{T} TTCATCGATAACATCTACCGTTACACCCTGGCCGGTACCGAAGTGTCCGCGCTGCTGGGT

PANSE_LMG5345{T} TTCATCGACAACATCTATCGTTACACCCTGGCCGGTACGGAAGTATCCGCGCTGCTGGGT

PANEU_LMG5346{T} TTCATCGACAACATCTACCGTTATACCCTGGCAGGTACTGAAGTATCTGCACTGCTGGGT

PANDI_CCUG25232{T} TTCATCGACAACATCTACCGTTACACCCTGGCCGGTACAGAGGTTTCTGCACTGCTGGGT

PANWA_LMG26277{T} TTCATCGACAACATCTACCGTTACACCCTGGCCGGTACTGAAGTATCAGCACTGCTGGGT

PANRO_LMG26273{T} TTCATCGACAACATCTATCGTTACACCCTGGCCGGTACTGAAGTATCAGCTCTGCTGGGT

PANRW_LMG26275{T} TTCATCGACAACATCTATCGTTACACCCTGGCCGGTACTGAAGTATCAGCACTGCTGGGT

PANCY_LMG2657{T} TTCATCGACAACATCTATCGTTACACCCTGGCCGGTACAGAAGTTTCTGCACTGCTGGGT

PANST_CCUG26359{T} TTCATCGACAACATCTACCGTTATACCCTGGCCGGTACAGAAGTCTCTGCACTGCTGGGT

PANST_LMG2632{PT} TTCATCGACAACATCTACCGTTATACCCTGGCCGGTACAGAAGTCTCTGCACTGCTGGGT

PANAL_LMG24248{T} TTCATCGACAACATCTACCGTTATACCCTGGCCGGTACGGAAGTATCAGCACTGCTGGGT

PANAN_LMG2665{T} TTCATCGACAACATCTACCGTTATACCCTGGCCGGTACGGAAGTATCCGCGCTGCTGGGT

PANAN_RSA47 TTCATCGACAACATCTACCGTTATACCCTGGCCGGTACGGAAGTATCAGCACTGCTGGGT

TATSA_NML06-3099{T} TTCGTCGATAACATTTACCGTTATACCCTGGCAGGTACTGAAGTTTCAGCACTGTTAGGC

TATPT_ATCC33301{T} TTCGTTGATAACATCTATCGTTATACCCTGGCCGGTACTGAAGTTTCAGCACTGCTGGGT

TATCI_DSM13699{T} TTCGTAGATAACATTTACCGTTATACCCTGGCCGGTACTGAAGTATCAGCTCTGCTGGGT

TATMO_LMG23360{T} TTTGTAGATAACATTTACCGTTATACCCTGGCCGGTACTGAAGTATCAGCTCTGCTGGGT

BURGL_LMG2196{T} TTCGTCGACAACATCTACCGTTTCACGCTGGCCGGGACCGAAGTGTCGGCCCTGCTGGGC

XANOR_ATCC35933{T} TTCGTGGACAACATTTACCGCTACACGCTGGCCGGTACCGAAGTGTCCGCGCTGCTCGGC

XANOR_CFBP2286{PT} TTCGTGGACAACATTTACCGCTACACGCTGGCCGGTACCGAAGTGTCCGCGCTGCTCGGC

PSEFU_LMG2158{T} TTCGTCGACAACATCTACCGTTACACCCTGGCCGGTACCGAAGTATCCGCACTGCTGGGC

PSEOR_KCTC32247 TTCGTCGACAACATCTACCGCTACACCCTGGCCGGTACCGAAGTGTCCGCGCTGCTGGGC

** * ** ***** * ** * ** * *** ** * ** ** ** ** *** * **

SPHME_DAPP-PG224{T} CGCATCCCGTCGGCGGTGGGCTACCAGCCGACGCTGTCGACCGACATGGGCGCGCTGCAG

SPHPA_NCTC11030{T} CGCATTCCGTCGGCCGTGGGCTATCAGCCGACCCTGTCGACCGACATGGGCGCGCTGCAG

PANAN_LMG2558{T} CGTATGCCATCTGCGGTAGGTTACCAGCCAACGCTGGCAGAAGAGATGGGTGTGTTGCAG

PANBR_LMG5343{T} CGTATGCCATCTGCGGTAGGTTACCAGCCAACGCTGGCCGAAGAGATGGGTGTGTTGCAG

PANCO_LMG24534{T} CGTATGCCATCTGCGGTAGGTTACCAGCCAACGCTGGCAGAAGAGATGGGTGTGTTGCAG

PANDE_LMG24200{T} CGTATGCCATCTGCAGTAGGTTATCAGCCAACGCTGGCCGAAGAGATGGGTGTGTTGCAG

PANVA_LMG24199{T} CGTATGCCATCTGCGGTAGGTTACCAGCCAACGCTGGCAGAAGAGATGGGCGTGTTGCAG

PANEU_LMG24197{T} CGTATGCCATCTGCGGTAGGTTACCAGCCAACGCTCGCAGAAGAGATGGGTGTGTTGCAG

PANAG_DSM3493{T} CGTATGCCATCTGCGGTAGGTTACCAGCCAACGCTGGCAGAAGAGATGGGTGTGTTGCAG

PANAG_CFBP13505 CGTATGCCATCTGCGGTAGGTTACCAGCCAACGCTGGCAGAAGAGATGGGTGTGTTGCAG

MIXAL_LTYR-11Z{T} CGTATGCCGTCTGCAGTAGGTTATCAGCCGACGCTGGCGGAAGAGATGGGTGTGTTGCAG

MIXTH_QC88-366{T} CGTATGCCGTCTGCAGTAGGCTATCAGCCGACGCTGGCGGAAGAGATGGGCGTATTGCAG

MIXCA_DSM22759{T} CGTATGCCGTCCGCAGTAGGCTATCAGCCGACGCTGGCGGAAGAGATGGGCGTGCTGCAG

MIXGA_DSM22758{T} CGTATGCCGTCTGCAGTAGGCTACCAGCCGACGCTGGCGGAAGAGATGGGCGTGCTGCAG

PANSE_LMG5345{T} CGTATGCCGTCAGCGGTAGGCTACCAGCCGACCCTGGCTGAAGAGATGGGCGTGCTGCAG

PANEU_LMG5346{T} CGTATGCCATCTGCGGTAGGTTATCAGCCAACGCTGGCTGAAGAGATGGGTGTGCTGCAG

PANDI_CCUG25232{T} CGTATGCCATCGGCGGTAGGTTATCAGCCAACGCTGGCTGAAGAGATGGGTGTGCTGCAG

PANWA_LMG26277{T} CGTATGCCATCTGCGGTAGGTTACCAGCCAACGCTGGCCGAAGAGATGGGTGTGTTGCAG

PANRO_LMG26273{T} CGTATGCCATCTGCGGTAGGCTACCAGCCAACGCTGGCAGAAGAGATGGGTGTGTTGCAG

PANRW_LMG26275{T} CGTATGCCATCTGCGGTAGGCTACCAGCCAACGCTGGCAGAAGAGATGGGTGTGTTGCAG

PANCY_LMG2657{T} CGTATGCCTTCAGCGGTAGGTTATCAGCCGACTCTGGCTGAAGAGATGGGTGTGTTGCAG

PANST_CCUG26359{T} CGTATGCCATCTGCGGTAGGTTATCAGCCAACGCTGGCAGAAGAGATGGGTGTGTTGCAG

PANST_LMG2632{PT} CGTATGCCATCTGCGGTAGGTTATCAGCCAACGCTGGCAGAAGAGATGGGTGTGTTGCAG

PANAL_LMG24248{T} CGTATGCCATCTGCGGTAGGTTATCAGCCAACGCTGGCAGAAGAGATGGGTGTGTTGCAG

PANAN_LMG2665{T} CGTATGCCATCTGCGGTAGGTTATCAGCCAACGCTGGCAGAAGAGATGGGTGTGTTGCAG

PANAN_RSA47 CGTATGCCATCTGCGGTAGGTTATCAGCCAACGCTGGCAGAAGAGATGGGTGTATTGCAG

TATSA_NML06-3099{T} CGTATGCCTTCTGCGGTAGGTTATCAGCCAACACTGGCTGAAGAGATGGGTGTCCTGCAG

TATPT_ATCC33301{T} CGTATGCCTTCTGCGGTAGGTTATCAGCCAACACTGGCCGAAGAAATGGGTGTTCTTCAG

TATCI_DSM13699{T} CGTATGCCTTCTGCGGTAGGTTATCAGCCAACACTGGCAGAAGAGATGGGTGTGCTGCAG

TATMO_LMG23360{T} CGTATGCCTTCTGCGGTAGGTTATCAGCCAACACTGGCAGAAGAGATGGGTGTGCTGCAG

BURGL_LMG2196{T} CGGATGCCGTCGGCAGTGGGCTATCAGCCGACGCTGGCCGAGGAAATGGGCAAGCTGCAG

XANOR_ATCC35933{T} CGTATGCCGTCGGCCGTGGGTTACCAGCCCACTCTGGCCGAAGAAATGGGCGTGCTGCAG

XANOR_CFBP2286{PT} CGTATGCCGTCGGCCGTGGGTTACCAGCCCACTCTGGCCGAAGAAATGGGCGTGCTGCAG

PSEFU_LMG2158{T} CGTATGCCTTCGGCAGTAGGTTACCAGCCGACCCTGGCTGAAGAGATGGGCGTGCTGCAA

PSEOR_KCTC32247 CGTATGCCGTCCGCGGTGGGTTACCAGCCGACCCTGGCCGAGGAAATGGGCGTTCTGCAG

** ** ** ** ** ** ** ** ***** ** ** * ** ***** * **

SPHME_DAPP-PG224{T} GAGCGCATCACCTCGACCAACAAGGGCTCGATCACCTCGGTGCAGGCCGTCTACGTCCCC

SPHPA_NCTC11030{T} GAGCGCATCACCTCGACCAACAAGGGCTCGATCACGAGCGTCCAGGCCGTGTACGTGCCC

PANAN_LMG2558{T} GAGCGTATTACCTCCACCAAGACCGGTTCAATCACCTCCGTACAGGCCGTTTACGTTCCT

PANBR_LMG5343{T} GAGCGTATTACCTCCACTAAGACCGGTTCAATCACTTCCGTACAGGCCGTTTACGTCCCT

PANCO_LMG24534{T} GAGCGTATTACCTCCACCAAGACCGGTTCAATCACTTCCGTACAGGCCGTTTACGTCCCT

PANDE_LMG24200{T} GAGCGTATTACCTCCACCAAGACCGGTTCAATCACCTCCGTACAGGCCGTTTACGTCCCT

PANVA_LMG24199{T} GAGCGTATTACCTCCACCAAGACTGGTTCAATCACCTCCGTACAGGCCGTTTACGTCCCT

PANEU_LMG24197{T} GAGCGTATTACCTCCACCAAGACCGGTTCAATCACTTCCGTACAGGCCGTTTACGTCCCT

PANAG_DSM3493{T} GAGCGTATTACCTCCACCAAGACCGGTTCAATCACCTCCGTACAGGCCGTTTACGTCCCT

PANAG_CFBP13505 GAGCGTATTACCTCCACCAAGACCGGTTCAATCACCTCCGTACAGGCCGTTTACGTCCCT

MIXAL_LTYR-11Z{T} GAGCGTATTACCTCCACCAAGACGGGTTCAATCACCTCTGTACAGGCCGTTTACGTACCT

MIXTH_QC88-366{T} GAGCGTATTACCTCCACCAAGACCGGTTCAATCACCTCCGTACAGGCCGTTTACGTTCCT

MIXCA_DSM22759{T} GAGCGTATTACCTCCACCAAGACCGGTTCAATCACCTCCGTACAGGCCGTATACGTCCCT

MIXGA_DSM22758{T} GAGCGTATTACCTCCACCAAGACCGGTTCAATCACCTCTGTACAGGCCGTATACGTCCCG

PANSE_LMG5345{T} GAGCGTATTACCTCCACCAAGACCGGTTCAATCACCTCCGTACAGGCCGTTTACGTCCCT

PANEU_LMG5346{T} GAGCGTATTACCTCCACCAAGACTGGTTCAATCACCTCCGTACAGGCCGTTTACGTTCCT

PANDI_CCUG25232{T} GAGCGTATTACCTCCACCAAGACCGGTTCAATCACCTCCGTACAGGCCGTTTACGTTCCT

PANWA_LMG26277{T} GAGCGTATTACCTCCACCAAAACCGGTTCAATCACCTCCGTACAGGCCGTTTACGTCCCT

PANRO_LMG26273{T} GAGCGTATTACCTCCACTAAGACCGGTTCAATCACCTCTGTTCAGGCCGTTTACGTTCCT

PANRW_LMG26275{T} GAGCGTATTACCTCCACCAAGACTGGTTCAATCACCTCTGTTCAGGCCGTTTACGTTCCT

PANCY_LMG2657{T} GAGCGTATTACCTCCACCAAGACCGGTTCAATCACTTCCGTACAGGCCGTTTACGTTCCT

PANST_CCUG26359{T} GAGCGTATTACCTCCACCAAAACCGGTTCAATCACCTCCGTACAGGCCGTTTACGTCCCT

PANST_LMG2632{PT} GAGCGTATTACCTCCACCAAAACCGGTTCAATCACCTCCGTACAGGCCGTTTACGTCCCT

PANAL_LMG24248{T} GAGCGTATTACCTCCACCAAGACAGGTTCAATCACCTCCGTACAGGCCGTTTACGTGCCA

PANAN_LMG2665{T} GAGCGTATTACCTCCACCAAGACAGGTTCAATCACCTCCGTACAGGCCGTATACGTGCCA

PANAN_RSA47 GAGCGTATTACCTCCACCAAGACAGGTTCAATCACCTCCGTACAGGCCGTATATGTGCCA

TATSA_NML06-3099{T} GAACGTATCACTTCAACCAAAACCGGTTCTATCACTTCCGTACAGGCCGTTTACGTCCCT

TATPT_ATCC33301{T} GAACGTATCACGTCAACCAAAACCGGTTCAATCACTTCCGTACAGGCAGTGTATGTACCT

TATCI_DSM13699{T} GAACGTATCACGTCAACCAAAACAGGTTCTATCACATCCGTACAGGCTGTGTATGTTCCT

TATMO_LMG23360{T} GAACGTATCACGTCAACCAAAACAGGTTCTATCACCTCCGTACAGGCGGTGTATGTTCCT

BURGL_LMG2196{T} GAGCGCATCACGTCGACCAAGACCGGCTCGATCACGTCCGTGCAGGCCGTGTACGTGCCG

XANOR_ATCC35933{T} GAACGCATCACCTCGACCAAGAGCGGTTCGATCACCTCGATCCAGGCCGTGTACGTGCCT

XANOR_CFBP2286{PT} GAACGCATCACCTCGACCAAGAGCGGTTCGATCACCTCGATCCAGGCGGTGTACGTGCCT

PSEFU_LMG2158{T} GAGCGCATCACTTCGACCAAGCAAGGTTCGATCACTTCGATCCAGGCCGTATACGTACCT

PSEOR_KCTC32247 GAGCGCATCACCTCCACCAAGAACGGCTCGATCACCTCGGTGCAGGCCGTCTACGTTCCC

** ** ** ** ** ** ** ** ** ***** * ***** ** ** ** **

SPHME_DAPP-PG224{T} GCGGACGATTTGACCGACCCGGCGCCGGCGACCTCGTTCGCCCACTTGGACGCAACGACC

SPHPA_NCTC11030{T} GCCGACGACTTGACCGACCCGGCGCCGGCCACCTCGTTCGCACACTTGGACGCGACGACC

PANAN_LMG2558{T} GCGGATGACCTGACTGACCCGTCACCAGCGACCACCTTCGCGCACCTGGACTCAACCGTT

PANBR_LMG5343{T} GCGGATGACCTGACTGACCCGTCACCAGCAACTACCTTTGCGCACTTAGACTCAACGGTA

PANCO_LMG24534{T} GCGGATGACCTGACTGACCCGTCTCCAGCCACCACCTTTGCGCACTTAGACTCAACGGTA

PANDE_LMG24200{T} GCGGATGACCTGACTGACCCGTCACCAGCAACCACCTTTGCGCACTTAGACTCAACGGTA

PANVA_LMG24199{T} GCGGATGACCTGACTGACCCATCACCAGCAACCACCTTTGCGCACTTAGACTCAACGGTA

PANEU_LMG24197{T} GCGGATGACCTGACTGACCCGTCACCAGCAACCACCTTTGCGCACTTAGACTCAACGGTA

PANAG_DSM3493{T} GCGGATGACCTGACTGACCCATCACCAGCAACTACCTTTGCGCACTTAGACTCAACGGTA

PANAG_CFBP13505 GCGGATGACCTGACTGACCCATCACCAGCAACTACCTTTGCGCACTTAGACTCAACGGTA

MIXAL_LTYR-11Z{T} GCGGATGACTTGACTGACCCGTCTCCGGCGACCACCTTCGCCCACCTTGACTCTACCGTT

MIXTH_QC88-366{T} GCGGATGACTTGACTGACCCGTCTCCGGCGACCACCTTCGCCCACCTTGATTCTACCGTT

MIXCA_DSM22759{T} GCGGATGACTTGACTGACCCGTCTCCGGCGACCACCTTCGCCCACCTCGACTCAACCGTT

MIXGA_DSM22758{T} GCGGATGACTTGACTGACCCGTCTCCGGCGACCACCTTCGCCCACCTCGACTCCACCGTT

PANSE_LMG5345{T} GCGGATGACTTGACTGACCCGTCTCCGGCGACCACCTTCGCCCACCTGGACTCAACCGTT

PANEU_LMG5346{T} GCGGATGACCTGACTGACCCGTCTCCGGCAACCACCTTTGCTCACTTAGACTCAACGGTT

PANDI_CCUG25232{T} GCGGATGACCTGACTGACCCGTCACCAGCAACCACCTTTGCTCACTTAGACTCAACCGTT

PANWA_LMG26277{T} GCGGATGACCTGACTGACCCGTCACCAGCAACTACCTTTGCTCACTTAGACTCAACCGTT

PANRO_LMG26273{T} GCGGATGACTTGACTGACCCGTCACCAGCAACCACCTTTGCTCACTTAGACTCAACCGTT

PANRW_LMG26275{T} GCGGATGACTTGACTGACCCGTCACCAGCAACCACCTTTGCTCACTTGGACTCAACCGTT

PANCY_LMG2657{T} GCGGATGACTTGACTGACCCGTCTCCGGCGACCACCTTCGCCCACCTGGACTCAACTGTA

PANST_CCUG26359{T} GCGGATGACCTGACTGACCCGTCACCGGCAACCACCTTTGCTCACTTAGACTCAACAGTC

PANST_LMG2632{PT} GCGGATGACCTGACTGACCCGTCACCGGCAACCACCTTTGCTCACTTAGACTCAACAGTC

PANAL_LMG24248{T} GCGGATGACTTGACTGACCCGTCACCAGCAACCACCTTTGCTCACTTAGACTCAACAGTC

PANAN_LMG2665{T} GCGGATGACTTGACTGACCCGTCACCAGCAACCACCTTTGCTCACTTAGACTCAACAGTT

PANAN_RSA47 GCGGATGACTTGACTGACCCGTCACCAGCAACCACCTTTGCTCACTTAGACTCAACAGTC

TATSA_NML06-3099{T} GCGGATGACTTGACTGACCCGTCTCCGGCAACCACCTTTGCTCACTTGGATTCCACGGTG

TATPT_ATCC33301{T} GCGGATGACCTGACTGACCCGTCTCCGGCAACAACCTTTGCTCACCTGGACTCAACAGTC

TATCI_DSM13699{T} GCGGATGACCTTACTGACCCGTCTCCGGCAACTACCTTTGCTCACCTGGACTCAACAGTT

TATMO_LMG23360{T} GCGGATGACCTGACTGACCCGTCTCCGGCAACTACCTTTGCTCACCTGGACTCAACCGTG

BURGL_LMG2196{T} GCGGATGACTTGACCGACCCGTCGCCCGCCACCACCTTCGGCCACCTGGACGCCACCGTC

XANOR_ATCC35933{T} GCGGACGACCTGACCGACCCGTCGCCGGCGACCACCTTCGCTCATTTGGACTCGACCGTC

XANOR_CFBP2286{PT} GCGGACGACCTGACCGACCCGTCGCCAGCGACCACCTTCGCCCACTTGGACTCGACCGTC

PSEFU_LMG2158{T} GCGGACGACCTGACCGACCCGTCGCCGGCGACCACCTTCGCCCACTTGGACGCCACCGTT

PSEOR_KCTC32247 GCGGACGACCTGACCGACCCGAGCCCGGCGACCACCTTCGCCCACCTCGACGCCACCGTG

** ** ** * ** ***** ** ** ** * ** * ** * ** * **

SPHME_DAPP-PG224{T} GTGCTCAACCGCGCGATCTCGGAACTTGGCATCTATCCGGCGGTCGATCCGCTCGATTCG

SPHPA_NCTC11030{T} GTGCTCAACCGCGCCATCTCGGAGCTGGGCATCTACCCGGCCGTCGACCCGCTCGACTCG

PANAN_LMG2558{T} ACGCTGAGCCGTCAGATTGCCTCTCTGGGTATCTACCCGGCCGTTGATCCGCTGGATTCT

PANBR_LMG5343{T} ACGCTGAGCCGTCAGATCGCCTCTCTGGGTATCTACCCGGCCGTTGACCCGCTGGATTCC

PANCO_LMG24534{T} ACGCTGAGCCGTCAGATCGCCTCTCTGGGTATTTACCCGGCTGTTGACCCGCTGGATTCC

PANDE_LMG24200{T} ACGCTGAGCCGTCAGATCGCCTCTCTGGGTATCTACCCGGCCGTTGACCCGCTGGATTCC

PANVA_LMG24199{T} ACGCTGAGCCGTCAGATCGCCTCTCTGGGTATCTACCCGGCCGTTGACCCGCTGGACTCT

PANEU_LMG24197{T} ACGCTGAGCCGTCAGATCGCATCTCTGGGTATCTACCCGGCCGTTGACCCACTGGACTCT

PANAG_DSM3493{T} ACGCTGAGCCGTCAGATCGCCTCTCTGGGTATCTACCCGGCCGTTGACCCGCTGGACTCT

PANAG_CFBP13505 ACGCTGAGCCGTCAGATCGCCTCTCTGGGTATCTACCCGGCCGTTGACCCGCTGGATTCT

MIXAL_LTYR-11Z{T} ACTCTGAGCCGTCAGATCGCGTCTCTGGGTATCTACCCGGCCGTTGACCCGCTGGATTCC

MIXTH_QC88-366{T} ACCCTGAGCCGTCAGATCGCGTCTCTGGGTATCTACCCGGCCGTTGACCCGCTGGACTCC

MIXCA_DSM22759{T} ACGCTGAGCCGTCAGATCGCGTCTCTGGGTATCTACCCGGCCGTTGACCCGCTGGACTCC

MIXGA_DSM22758{T} ACGCTGAGCCGTCAGATCGCGTCTCTGGGTATCTACCCGGCCGTTGACCCGCTGGATTCC

PANSE_LMG5345{T} ACCCTGAGCCGTCAGATCGCGTCTCTGGGTATCTACCCGGCCGTTGACCCGCTGGATTCC

PANEU_LMG5346{T} ACGCTGAGCCGTCAGATCGCCTCTCTGGGTATCTACCCGGCCGTTGACCCGCTGGACTCT

PANDI_CCUG25232{T} ACGCTGAGCCGTCAGATCGCGTCACTGGGTATCTACCCGGCCGTTGACCCGCTGGACTCT

PANWA_LMG26277{T} ACGCTGAGCCGTCAGATCGCCTCTCTGGGTATCTACCCGGCCGTTGACCCGCTGGATTCA

PANRO_LMG26273{T} ACGCTGAGCCGTCAGATCGCCTCTCTGGGTATCTACCCAGCCGTTGACCCGCTGGATTCA

PANRW_LMG26275{T} ACGCTGAGCCGTCAGATCGCCTCTCTGGGTATCTACCCAGCCGTTGACCCGCTGGATTCA

PANCY_LMG2657{T} ACCCTGAGCCGTCAGATCGCTTCTCTGGGTATCTACCCGGCCGTTGACCCGCTGGATTCC

PANST_CCUG26359{T} ACCCTGAGCCGTCAGATCGCCTCTCTGGGTATCTACCCAGCCGTTGATCCGCTGGACTCA

PANST_LMG2632{PT} ACCCTGAGCCGTCAGATCGCCTCTCTGGGTATCTACCCAGCCGTTGATCCGCTGGACTCA

PANAL_LMG24248{T} ACCCTGAGCCGTCAGATCGCCTCTCTGGGTATCTACCCGGCAGTTGATCCGCTGGACTCC

PANAN_LMG2665{T} ACCCTGAGCCGTCAGATCGCCTCGCTGGGTATCTACCCGGCCGTTGACCCGCTGGACTCC

PANAN_RSA47 ACCCTGAGCCGTCAGATCGCCTCTCTGGGTATCTACCCGGCCGTTGACCCGCTGGACTCC

TATSA_NML06-3099{T} ACTCTGAGCCGTCAGATCGCCTCTCTGGGTATCTACCCAGCCGTTGACCCGCTGGATTCC

TATPT_ATCC33301{T} ACCCTGAGCCGTCAGATTGCATCCCTGGGTATTTACCCGGCGGTTGACCCGCTGGATTCC

TATCI_DSM13699{T} ACTCTGAGCCGTCAGATTGCGTCACTGGGTATTTACCCGGCTGTTGACCCGCTGGATTCA

TATMO_LMG23360{T} ACTCTGAGCCGTCAGATTGCATCCCTGGGTATCTACCCGGCCGTTGACCCGCTGGATTCC

BURGL_LMG2196{T} GTGCTGTCGCGTGACATCGCCTCGCTGGGCATCTACCCGGCCGTCGATCCGCTCGATTCC

XANOR_ATCC35933{T} ACGCTGAGCCGTAACATCGCTTCGCTGGGTATCTACCCGGCTGTGGATCCGCTGGACTCC

XANOR_CFBP2286{PT} ACGCTGAGCCGTAACATCGCTTCGCTGGGTATCTACCCGGCTGTGGATCCGCTGGACTCC

PSEFU_LMG2158{T} GTACTGTCCCGTGACATCGCTTCCCTGGGTATCTACCCAGCGGTAGACCCACTGGACTCG

PSEOR_KCTC32247 GTACTGTCCCGTGACATCGCCTCCCTGGGTATCTACCCGGCCGTCGATCCGCTGGACTCC

** ** ** * ** ** ** ** ** ** ** ** ** ** ** **

SPHME_DAPP-PG224{T} ACCAGCCGCGTGCTTGAACCGCGTATCGTCGGCCAGGAGCATTACGACACGGCGCGCGCC

SPHPA_NCTC11030{T} ACCAGCCGCGTGCTGGAGCCGCGCATCGTCGGTCAGGAGCATTATGAGACGGCCCGTGCG

PANAN_LMG2558{T} ACCAGCCGTCAGCTGGATCCGCTGGTTGTGGGTCAGGAGCACTATGATGTTGCACGTGGC

PANBR_LMG5343{T} ACCAGCCGTCAGCTGGATCCACTGGTTGTGGGTCAGGAGCACTACGACGTAGCGCGTGGC

PANCO_LMG24534{T} ACCAGCCGTCAGCTGGATCCACTGGTTGTGGGTCAGGAGCACTACGACGTAGCCCGTGGC

PANDE_LMG24200{T} ACCAGCCGTCAGCTGGATCCACTGGTTGTGGGTCAGGAGCACTATGATGTTGCGCGTGGC

PANVA_LMG24199{T} ACCAGCCGTCAGCTGGATCCGCTGGTTGTCGGTCAGGAACACTATGATGTTGCGCGTGGC

PANEU_LMG24197{T} ACCAGCCGTCAGCTGGATCCGCTGGTTGTTGGTCAGGAACACTATGATGTTGCGCGTGGC

PANAG_DSM3493{T} ACCAGCCGTCAGCTGGATCCGCTGGTTGTCGGTCAGGAGCACTATGATGTTGCACGTGGC

PANAG_CFBP13505 ACCAGCCGTCAGCTGGATCCGCTGGTTGTCGGTCAGGAGCACTATGATGTTGCACGTGGC

MIXAL_LTYR-11Z{T} ACCAGCCGTCAGCTGGATCCGCTGGTTGTAGGTCAGGAGCACTACGATACGGCGCGTGGC

MIXTH_QC88-366{T} ACCAGCCGTCAGCTGGATCCGCTGGTTGTAGGTCAGGAGCATTACGATACGGCGCGTGGC

MIXCA_DSM22759{T} ACCAGCCGTCAGCTGGACCCGCTGGTTGTTGGTCAGGAGCACTACGACACCGCGCGTGGC

MIXGA_DSM22758{T} ACCAGCCGTCAGCTGGATCCGCTGGTTGTGGGTCAGGAACACTACGACACGGCGCGTGGC

PANSE_LMG5345{T} ACCAGCCGTCAGCTGGATCCGCTGGTGGTTGGCCAGGAGCACTACGACGTTGCGCGCGGC

PANEU_LMG5346{T} ACCAGCCGTCAGCTGGATCCACTGATCGTGGGTCAGGAGCACTATGACGTCGCACGTGGC

PANDI_CCUG25232{T} ACCAGCCGTCAGCTGGATCCGCTGATCGTCGGTCAGGAGCACTACGACGTCGCGCGTGGC

PANWA_LMG26277{T} ACCAGCCGTCAGCTGGATCCACTGATCGTGGGTCAGGAGCACTACGACGTTGCGCGTGGC

PANRO_LMG26273{T} ACCAGCCGTCAGCTGGATCCACTGATCGTGGGTCAGGAGCACTATGACGTTGCGCGTGGC

PANRW_LMG26275{T} ACCAGCCGTCAGCTGGATCCACTCATCGTGGGTCAGGAGCACTATGACGTTGCACGTGGC

PANCY_LMG2657{T} ACCAGCCGTCAGCTGGATCCGCTGGTTGTTGGTCAGGAGCACTACGATGTAGCGCGTGGC

PANST_CCUG26359{T} ACCAGCCGTCAGCTGGATCCACTGGTTGTGGGTCAGGAGCACTACGATGTTGCACGTGGC

PANST_LMG2632{PT} ACCAGCCGTCAGCTGGATCCACTGGTTGTGGGTCAGGAGCACTACGATGTTGCACGTGGC

PANAL_LMG24248{T} ACCAGCCGTCAGCTGGACCCATTAGTTGTGGGTCAGGAGCACTATGACGTTGCACGTGGC

PANAN_LMG2665{T} ACCAGCCGTCAGCTGGATCCACTGGTTGTGGGTCAGGAGCACTACGACGTTGCACGTGGC

PANAN_RSA47 ACCAGCCGTCAGCTGGATCCACTGGTTGTGGGTCAGGAGCACTATGACGTTGCACGTGGC

TATSA_NML06-3099{T} ACCAGCCGTCAGCTGGATCCAATGATCGTGGGTCAGGAGCACTATGATGTGGCTCGTGGC

TATPT_ATCC33301{T} ACCAGCCGTCAGCTGGATCCAATGGTTGTAGGTCAGGAGCACTATGATATCGCCCGTGGC

TATCI_DSM13699{T} ACCAGTCGTCAGCTGGACCCAATGGTGGTTGGCCAGGAGCACTACGATGTAGCTCGTGGC

TATMO_LMG23360{T} ACCAGCCGTCAGCTGGACCCAATGGTAGTTGGCCAGGAGCACTACGATGTAGCTCGTGGC

BURGL_LMG2196{T} ACCTCGCGCCAGATCGACCCGAACGTGATCGGCGAGGAGCACTACACGATCACGCGCGGC

XANOR_ATCC35933{T} ACCAGCCGCCAGATGGACCCGCTGGTGATCGGCCACGAGCATTACGACACCGCCCAGCGC

XANOR_CFBP2286{PT} ACCAGCCGCCAGATGGACCCGCTGGTGATCGGTCACGAGCATTACGACACCGCCCAGCGC

PSEFU_LMG2158{T} ACTTCGCGCCAGCTGGATCCGAACGTCATCGGCAACGAGCACTACGAAACCGCTCGCGGT

PSEOR_KCTC32247 ACCTCCCGCCAGCTGGATCCGCTGGTGATCGGCCAGGAGCACTACGACACCGCCCGCGGC

** ** * * ** ** * * ** * ** ** ** * *

SPHME_DAPP-PG224{T} GTCCAGTCGCTGCTCCAGCGCTACAAGTCGCTGCAGGACATCATCGCCATCCTCGGCATG

SPHPA_NCTC11030{T} GTCCAGTCGCTGCTCCAGCGCTACAAGGCGCTGCAGGACATCATCGCGATCCTGGGCATG

PANAN_LMG2558{T} GTTCAGTCACTGCTGCAGCGTTATCAGGAGCTGAAAGACATCATCGCCATCCTCGGTATG

PANBR_LMG5343{T} GTTCAGTCACTGCTGCAGCGTTACCAGGAGCTGAAAGACATCATCGCCATCCTCGGTATG

PANCO_LMG24534{T} GTTCAGTCACTGCTGCAGCGTTACCAGGAGCTGAAAGACATCATCGCCATCCTCGGTATG

PANDE_LMG24200{T} GTTCAGTCACTGCTGCAGCGTTATCAGGAGCTGAAAGACATCATCGCCATCCTCGGTATG

PANVA_LMG24199{T} GTTCAGTCACTGCTGCAGCGTTATCAGGAACTGAAAGACATCATCGCCATCCTCGGTATG

PANEU_LMG24197{T} GTTCAGTCACTGCTGCAGCGTTATCAGGAACTGAAAGACATCATCGCCATCCTCGGTATG

PANAG_DSM3493{T} GTTCAGTCACTGCTGCAGCGTTATCAGGAACTGAAAGACATCATCGCCATCCTCGGTATG

PANAG_CFBP13505 GTTCAGTCACTGCTGCAGCGTTATCAGGAACTGAAAGACATCATCGCCATCCTCGGTATG

MIXAL_LTYR-11Z{T} GTGCAGTCTATTCTGCAGCGTTACCAGGAACTGAAAGACATCATCGCCATCCTTGGTATG

MIXTH_QC88-366{T} GTGCAGTCTATTCTGCAGCGTTACCAGGAACTGAAAGACATCATCGCCATCCTCGGTATG

MIXCA_DSM22759{T} GTGCAGTCTATTCTGCAGCGTTACCAGGAACTGAAAGACATCATCGCCATCCTGGGTATG

MIXGA_DSM22758{T} GTGCAGTCTATTCTGCAGCGTTACCAGGAACTGAAAGACATCATCGCCATCCTCGGTATG

PANSE_LMG5345{T} GTGCAGTCTCTGCTGCAGCGTTACCAGGAACTGAAAGACATCATCGCCATCCTCGGTATG

PANEU_LMG5346{T} GTACAGTCACTGCTGCAGCGTTACCAGGAGCTGAAAGACATCATCGCCATCCTCGGTATG

PANDI_CCUG25232{T} GTTCAGTCGCTGCTGCAGCGTTACCAGGAGCTGAAAGACATCATCGCCATCCTCGGCATG

PANWA_LMG26277{T} GTGCAGTCTCTGCTGCAGCGTTACCAGGAACTGAAAGACATCATCGCCATCCTCGGTATG

PANRO_LMG26273{T} GTGCAGTCTCTGCTGCAGCGTTACCAGGAACTGAAAGACATCATCGCCATCCTTGGTATG

PANRW_LMG26275{T} GTGCAGTCTCTGCTGCAGCGTTACCAGGAACTGAAAGACATCATCGCCATCCTTGGTATG

PANCY_LMG2657{T} GTACAGTCACTGCTGCAACGTTACCAGGAACTGAAAGACATCATCGCCATCCTCGGTATG

PANST_CCUG26359{T} GTACAGTCACTGCTGCAGCGTTATCAGGAACTGAAAGACATTATCGCCATCCTGGGTATG

PANST_LMG2632{PT} GTACAGTCACTGCTGCAGCGTTATCAGGAACTGAAAGACATTATCGCCATCCTGGGTATG

PANAL_LMG24248{T} GTACAGTCACTGCTGCAGCGTTATCAGGAACTGAAAGACATCATCGCCATCCTGGGTATG

PANAN_LMG2665{T} GTACAGTCACTGCTGCAGCGTTATCAGGAACTGAAAGACATCATCGCCATCCTGGGTATG

PANAN_RSA47 GTACAGTCACTGCTGCAGCGTTATCAGGAACTGAAAGACATCATCGCCATCCTGGGTATG

TATSA_NML06-3099{T} GTTCAGTCCCTGTTACAGCGTTACCAGGAACTGAAAGACATCATCGCGATCCTGGGTATG

TATPT_ATCC33301{T} GTGCAGTCTATTCTGCAACGTTATCAGGAACTGAAAGATATCATTGCTATTCTGGGTATG

TATCI_DSM13699{T} GTACAGTCTCTGTTACAGCGTTATCAGGAACTGAAAGACATCATCGCGATTCTGGGTATG

TATMO_LMG23360{T} GTTCAGTCTCTGTTACAGCGTTATCAGGAACTGAAAGACATCATCGCGATTCTGGGTATG

BURGL_LMG2196{T} GTGCAGCAGACGCTGCAGCGCTACAAGGAACTGCGCGACATCATCGCGATCCTGGGCATG

XANOR_ATCC35933{T} GTCCAGCAGACCTTGCAGAAGTACAAGGAACTGAAGGACATCATCGCCATCCTGGGCATG

XANOR_CFBP2286{PT} GTCCAGCAGACCTTGCAGAAGTACAAGGAACTGAAGGACATCATCGCCATCCTGGGCATG

PSEFU_LMG2158{T} GTTCAGTACGTGCTGCAGCGCTACAAGGAACTGAAGGACATCATCGCGATCCTGGGTATG

PSEOR_KCTC32247 GTGCAGTACGTGCTGCAGCGCTACAAGGAGCTGAAGGACATCATCGCGATCCTCGGCATG

** *** * ** ** ** *** ** ** ** ** ** ** ** ***

SPHME_DAPP-PG224{T} GACGAACTGTCCGAAGAGGATAAGCTGACGGTGACCCGCGCGCGCAAGATCCAGCGCTTC

SPHPA_NCTC11030{T} GACGAGCTGTCGGAAGAGGACAAGCTGACCGTCCAGCGCGCCCGCAAGATCCAGCGCTTC

PANAN_LMG2558{T} GATGAGCTGTCTGAAGAAGATAAACTGCTGGTGGCACGTGCGCGTAAGATTCAGCGCTTC

PANBR_LMG5343{T} GACGAGCTGTCTGAAGAAGATAAACTGCTGGTGGCACGTGCGCGTAAGATTCAGCGTTTC

PANCO_LMG24534{T} GACGAGCTGTCTGAAGAAGATAAACTGCTGGTGGCACGTGCGCGTAAGATTCAGCGCTTC

PANDE_LMG24200{T} GATGAGCTGTCTGAAGAAGATAAACTGCTGGTGGCACGTGCGCGTAAGATTCAGCGCTTC

PANVA_LMG24199{T} GATGAGCTGTCTGAAGAAGACAAACTGCTGGTGGCACGTGCGCGTAAAATTCAGCGCTTC

PANEU_LMG24197{T} GATGAGCTGTCTGAAGAAGACAAACTGCTGGTGGCACGTGCGCGTAAGATTCAGCGCTTC

PANAG_DSM3493{T} GATGAGCTGTCTGAAGAAGACAAACTGCTGGTGGCACGTGCGCGTAAGATTCAGCGCTTC

PANAG_CFBP13505 GATGAGCTGTCTGAAGAAGACAAACTGCTGGTGGCACGTGCGCGTAAGATTCAGCGCTTC

MIXAL_LTYR-11Z{T} GATGAACTGTCTGAAGAAGACAAACTGCTGGTGGCTCGCGCACGTAAAATTCAGCGCTTC

MIXTH_QC88-366{T} GACGAACTGTCTGAAGACGACAAACTGCTGGTGGCTCGCGCGCGTAAAATTCAGCGCTTC

MIXCA_DSM22759{T} GACGAACTGTCTGAAGACGACAAACTGCTGGTGGCGCGTGCCCGTAAGATTCAGCGCTTC

MIXGA_DSM22758{T} GACGAGCTGTCTGAAGATGACAAACTGCTGGTGGCACGTGCGCGTAAGATCCAGCGCTTC

PANSE_LMG5345{T} GACGAGCTGTCTGAAGACGACAAACTGCTGGTGGCTCGCGCACGTAAGATTCAGCGCTTC

PANEU_LMG5346{T} GACGAGCTGTCTGAAGAAGATAAACTGCTGGTGGCACGTGCGCGTAAGATCCAGCGCTTC

PANDI_CCUG25232{T} GACGAGCTGTCTGAAGAAGACAAACTGCTGGTGGCACGTGCGCGTAAGATTCAGCGCTTC

PANWA_LMG26277{T} GACGAGCTGTCTGAAGAAGACAAACTGCTGGTGGCACGTGCGCGTAAGATTCAGCGCTTC

PANRO_LMG26273{T} GACGAGCTGTCTGAAGAAGACAAACTGTTGGTGGCACGTGCGCGTAAGATTCAGCGCTTC

PANRW_LMG26275{T} GACGAACTGTCTGAAGAAGACAAACTGTTGGTGGCACGTGCGCGTAAGATTCAGCGCTTC

PANCY_LMG2657{T} GACGAGCTGTCTGAAGATGACAAACTGCTGGTGGCACGTGCGCGTAAAATTCAGCGCTTC

PANST_CCUG26359{T} GACGAACTGTCTGAAGAAGACAAACTGCTGGTGGCACGTGCGCGTAAGATTCAGCGCTTC

PANST_LMG2632{PT} GACGAACTGTCTGAAGAAGACAAACTGCTGGTGGCACGTGCGCGTAAGATTCAGCGCTTC

PANAL_LMG24248{T} GACGAACTGTCTGAAGAAGACAAACTGCTGGTGGCACGTGCGCGTAAGATCCAGCGCTTC

PANAN_LMG2665{T} GACGAGCTGTCTGAAGAAGACAAACTGCTGGTGGCACGTGCGCGTAAGATTCAGCGCTTC

PANAN_RSA47 GACGAGCTGTCTGAAGAAGACAAACTGCTGGTGGCACGTGCGCGTAAGATTCAGCGCTTC

TATSA_NML06-3099{T} GACGAACTGTCTGAGGAAGACAAACTGCTGGTATCACGTGCGCGTAAAATCCAGCGTTTC

TATPT_ATCC33301{T} GACGAACTGTCTGAAGACGACAAACTGCTGGTTTCACGTGCACGTAAAATTCAGCGCTTC

TATCI_DSM13699{T} GATGAACTGTCTGAAGACGACAAACTGCTGGTGTCTCGTGCACGTAAAATTCAGCGTTTC

TATMO_LMG23360{T} GATGAACTGTCTGAAGACGACAAACTGCTGGTGTCTCGTGCACGTAAAATTCAGCGTTTC

BURGL_LMG2196{T} GACGAACTGTCGCCGGAAGACAAGCTGACGGTCGCGCGCGCGCGGAAGATCCAGCGTTTC

XANOR_ATCC35933{T} GACGAGCTGAGCGAAGAAGACAAGCAGTCGGTGTCGCGCGCACGCAAGATCGAGCGCTTC

XANOR_CFBP2286{PT} GACGAGCTGAGCGAAGAAGACAAGCAGTCGGTGTCGCGCGCACGCAAGATCGAGCGCTTC

PSEFU_LMG2158{T} GACGAGCTGTCGGAAACCGACAAGCAGTTGGTATCCCGCGCTCGTAAGATCCAGCGTTTC

PSEOR_KCTC32247 GACGAACTGTCCGAAGCCGACAAGCAGCTGGTGTCCCGCGCCCGTAAGATCCAGCGCTTC

** ** *** ** ** * * ** ** ** ** ** ** **** ***

SPHME_DAPP-PG224{T} CTCAGCCAGCCGTTCCACGTCGCCGAAGTCTTCACCGGCATCAGCGGCAAGTTCGTGCAG

SPHPA_NCTC11030{T} CTGTCGCAGCCCTTCCACGTCGCGGAAGTCTTCACCGGCATCTCGGGCAAGTTCGTCCAG

PANAN_LMG2558{T} CTGTCTCAGCCGTTCTTCGTTGCGGAAGTATTCACCGGTTCACCGGGTAAATACGTTTCC

PANBR_LMG5343{T} CTGTCTCAGCCGTTCTTCGTTGCGGAAGTCTTCACCGGTTCTCCGGGCAAGTACGTTACG

PANCO_LMG24534{T} CTGTCTCAGCCGTTCTTCGTTGCGGAAGTCTTCACCGGTTCTCCGGGCAAGTACGTTACG

PANDE_LMG24200{T} CTGTCTCAGCCGTTCTTCGTTGCGGAAGTATTCACCGGTTCACCGGGCAAATACGTTACG

PANVA_LMG24199{T} CTGTCTCAGCCGTTCTTCGTTGCGGAAGTATTCACCGGTTCACCGGGTAAATACGTTACG

PANEU_LMG24197{T} CTGTCTCAGCCGTTCTTCGTTGCGGAAGTATTCACCGGTTCACCGGGTAAATACGTTACG

PANAG_DSM3493{T} CTGTCTCAGCCGTTCTTCGTTGCAGAAGTATTCACCGGTTCACCGGGCAAATACGTGACG

PANAG_CFBP13505 CTGTCTCAGCCGTTCTTCGTTGCAGAAGTATTCACCGGTTCACCGGGCAAATACGTGACG

MIXAL_LTYR-11Z{T} CTGTCTCAGCCGTTCTTCGTGGCAGAAGTATTCACCGGTTCTCCGGGCAAATACGTTTCG

MIXTH_QC88-366{T} CTGTCTCAGCCGTTCTTCGTGGCAGAAGTATTCACCGGTTCTCCGGGCAAATACGTTTCG

MIXCA_DSM22759{T} CTGTCTCAGCCGTTCTTCGTGGCAGAAGTATTCACCGGTTCTCCGGGCAAATACGTTCCG

MIXGA_DSM22758{T} CTGTCTCAGCCGTTCTTCGTTGCGGAAGTCTTCACCGGTTCTCCGGGTAAATACGTTACG

PANSE_LMG5345{T} CTGTCTCAGCCGTTCTTCGTAGCGGAAGTCTTCACCGGTGCGCCAGGTAAATACGTTACG

PANEU_LMG5346{T} CTGTCTCAGCCATTCTTCGTTGCTGAAGTCTTCACCGGTTCTCCGGGCAAATACGTGACG

PANDI_CCUG25232{T} CTGTCTCAGCCGTTCTTCGTAGCAGAAGTCTTCACCGGTTCTCCGGGTAAATACGTGACG

PANWA_LMG26277{T} CTGTCCCAGCCGTTCTTCGTTGCTGAAGTCTTCACCGGTTCTCCGGGTAAATACGTGACG

PANRO_LMG26273{T} CTGTCTCAGCCGTTCTTCGTTGCTGAAGTCTTCACCGGTTCTCCAGGTAAGTACGTGACC

PANRW_LMG26275{T} CTGTCTCAGCCGTTCTTCGTTGCTGAAGTCTTCACCGGTTCTCCAGGTAAGTACGTGACC

PANCY_LMG2657{T} CTGTCTCAGCCGTTCTTCGTTGCTGAAGTGTTCACCGGTTCTCCAGGTAAGTACGTATCG

PANST_CCUG26359{T} CTGTCTCAGCCGTTCTTCGTAGCAGAAGTGTTCACCGGTTCTCCAGGTAAATACGTGACG

PANST_LMG2632{PT} CTGTCTCAGCCGTTCTTCGTAGCAGAAGTGTTCACCGGTTCTCCAGGTAAATACGTGACG

PANAL_LMG24248{T} CTGTCTCAGCCGTTCTTCGTAGCAGAAGTGTTCACCGGTTCTCCAGGTAAATACGTGACG

PANAN_LMG2665{T} CTGTCTCAGCCGTTCTTCGTAGCAGAAGTGTTCACCGGTTCTCCAGGTAAATACGTAACG

PANAN_RSA47 CTGTCCCAGCCGTTCTTCGTAGCAGAAGTGTTCACCGGTTCTCCAGGTAAATACGTGACG

TATSA_NML06-3099{T} TTGTCTCAGCCATTCTTCGTGGCAGAGATCTTTACCGGTGCACCAGGTAAATACGTTTCT

TATPT_ATCC33301{T} CTGTCTCAGCCATTCTTCGTTGCAGAGATCTTTACCGGTGCGCCTGGTAAGTACGTTTCT

TATCI_DSM13699{T} CTGTCTCAGCCATTCTTCGTCGCAGAGATCTTTACCGGTGCACCTGGTAAATACGTTTCT

TATMO_LMG23360{T} CTGTCTCAGCCATTCTTCGTTGCAGAGATCTTTACCGGTGCACCTGGTAAATACGTTTCT

BURGL_LMG2196{T} CTGTCGCAGCCGTTCCACGTCGCGGAAGTGTTCACGGGCGCGCCGGGCAAGTACGTGCCG

XANOR_ATCC35933{T} TTCAGCCAGCCCTTCCACGTGGCCGAAGTGTTCACCGGCTCCCCGGGCAAGTACGTCTCG

XANOR_CFBP2286{PT} TTCAGCCAGCCCTTCCACGTGGCCGAAGTGTTCACCGGCTCCCCGGGCAAGTACGTCTCG

PSEFU_LMG2158{T} CTGTCGCAGCCGTTCTTCGTGGCTGAAGTCTTCACCGGTGCCTCGGGTAAATACGTTTCC

PSEOR_KCTC32247 CTGTCCCAGCCGTTCTTCGTGGCCGAAGTGTTCACCGGTTCGCCGGGCAAGTACGTGCCG

* ***** *** *** ** ** * ** ** ** ** ** * ***

SPHME_DAPP-PG224{T} ATCGAAGACACGATCCGCTCGTTCAAGGCGGTCGTCGACGGCGAATATGATCACCTGCCG

SPHPA_NCTC11030{T} ATCGAAGACACGATCCGTTCGTTCAAGGCGGTGGTCGACGGCGAGTACGACCATCTGCCG

PANAN_LMG2558{T} CTGAAAGACACTATCCGTGGCTTTAAAGGCATCATGGAAGGTGAGTTTGACCACCTGCCA

PANBR_LMG5343{T} CTGAAAGACACTATCCGTGGCTTTAAAGGCATCATGGAAGGTGAGTTTGACCACCTGCCA

PANCO_LMG24534{T} CTGAAAGACACTATCCGTGGCTTTAAAGGCATCATGGAAGGTGAGTTTGACCACCTGCCA

PANDE_LMG24200{T} CTGAAAGACACTATCCGTGGCTTTAAAGGCATCATGGAAGGTGAGTTTGACCACCTGCCA

PANVA_LMG24199{T} CTGAAAGACACTATCCGTGGCTTTAAAGGCATCATGGAAGGTGAGTTTGACCACCTGCCA

PANEU_LMG24197{T} CTGAAAGACACTATCCGTGGCTTTAAAGGCATCATGGAAGGTGAGTTTGACCACCTGCCA

PANAG_DSM3493{T} CTGAAAGACACTATCCGTGGCTTTAAAGGCATCATGGAAGGTGAGTTTGACCACCTGCCA

PANAG_CFBP13505 CTGAAAGACACTATCCGTGGCTTTAAAGGCATCATGGAAGGTGAGTTTGACCACCTGCCA

MIXAL_LTYR-11Z{T} CTGAAAGACACTATCCGTGGCTTTAAAGGCATTATGGACGGTGAGTTCGATCACCTGCCA

MIXTH_QC88-366{T} CTGAAAGACACTATTCGTGGCTTTAAAGGCATTATGGAAGGTGAGTTCGATCATCTGCCA

MIXCA_DSM22759{T} CTGAAAGACACCATCCGTGGCTTTAAAGGCATTATGGAAGGTGAATTCGACCACCTGCCA

MIXGA_DSM22758{T} CTGAAAGACACCATCCGTGGCTTTAAAGGCATTATGGAAGGTGAGTTCGACCACCTGCCA

PANSE_LMG5345{T} CTGAAAGACACTATCCGTGGCTTTAAAGGCATTATGGACGGCGAGTTCGACCATCTGCCG

PANEU_LMG5346{T} CTGAAAGATACCATTCGTGGTTTCAAAGGCATCATGGAAGGCGAATTCGACCACCTGCCA

PANDI_CCUG25232{T} CTGAAAGACACCATCCGTGGCTTCAAAGGCATCATGGAAGGCGAATTCGACCACCTGCCA

PANWA_LMG26277{T} CTGAAAGACACCATCCGTGGCTTTAAAGGCATCATGGAAGGCGAATTCGACCACCTGCCA

PANRO_LMG26273{T} CTGAAAGACACGATTCGTGGCTTCAAAGGCATCATGGAAGGCGAATTCGACCACCTGCCA

PANRW_LMG26275{T} CTGAAAGACACGATTCGTGGCTTCAAAGGCATCATGGAAGGCGAATTCGACCACCTGCCA

PANCY_LMG2657{T} CTGAAAGACACCATCCGTGGCTTCAAAGGCATCATGGAAGGTGAATTCGACCATCTGCCA

PANST_CCUG26359{T} CTGAAAGACACCATCCGTGGCTTTAAAGGCATCATGGAAGGTGAATTTGACCACCTGCCA

PANST_LMG2632{PT} CTGAAAGACACCATCCGTGGCTTTAAAGGCATCATGGAAGGTGAATTTGACCACCTGCCA

PANAL_LMG24248{T} CTGAAAGACACCATCCGTGGCTTTAAAGGCATCATGGAAGGTGAATTTGACCACCTGCCA

PANAN_LMG2665{T} CTGAAAGACACCATCCGTGGCTTTAAAGGCATCATGGAAGGTGAATTTGACCACCTGCCA

PANAN_RSA47 CTGAAAGACACCATCCGTGGCTTTAAAGGCATCATGGAAGGTGAATTTGACCACCTGCCA

TATSA_NML06-3099{T} CTGAAAGAAACCATCCGAGGCTTCAAAGGTATTATGGAAGGTGAATTCGACCATATGCCA

TATPT_ATCC33301{T} CTGAAAGACACCATCCGTGGCTTCAAAGGTATTATGGACGGTGAGTTTGACCATATGCCT

TATCI_DSM13699{T} CTGAAAGACACCATCCGTGGCTTCAAAGGAATTATGGATGGTGAGTTTGACCATCTGCCT

TATMO_LMG23360{T} CTGAAAGACACCATCCGTGGCTTCAAAGGAATTATGGATGGCGAGTTTGACCATCTGCCT

BURGL_LMG2196{T} CTGAAGGAAACCATCCGCGGCTTCAAGATGATCGTCGACGGCGAGTGTGATCACCTGCCG

XANOR_ATCC35933{T} CTGAAGGACACCATCCGCGGCTTCAAGGCGATCTGCGACGGCGAATACGACCACCTACCG

XANOR_CFBP2286{PT} CTGAAGGACACCATCCGCGGCTTCAAGGCGATCTGCGACGGCGAATACGACCACCTGCCG

PSEFU_LMG2158{T} CTGAAAGACACCATTGCTGGCTTCAAAGGCATCCTCAACGGTGACTACGACCACCTGCCA

PSEOR_KCTC32247 CTGAAGGAAACCATCCGCGGCTTCTCCGGCATCCTCAATGGCGACTACGACCACCTGCCG

* * ** ** ** ** * * ** ** * ** ** * **

SPHME_DAPP-PG224{T} GAAGCAGCCTTCTACATGGTCGGCGGCATCGACGAAGTCGTCGCCAAGGCCGAAAAGATG

SPHPA_NCTC11030{T} GAAGCGGCCTTCTACATGGTCGGCGGCATCGACGAAGCCGTTGCCAAGGCCGAGAAGCTG

PANAN_LMG2558{T} GAGCAGGCCTTCTACATGGTTGGTGCCATCGAAGAAGCCGTGGAAAAAGCGAAGAAACT-

PANBR_LMG5343{T} GAGCAGGCCTTCTACATGGTTGGTGCCATCGAAGAAGCCGTGGAAAAAGCGAAGAAACT-

PANCO_LMG24534{T} GAGCAGGCCTTCTACATGGTTGGTGCCATCGAAGAAGCCGTGGAAAAAGCGAAGAAACT-

PANDE_LMG24200{T} GAGCAGGCCTTCTACATGGTTGGTGCCATCGAAGAAGCCGTGGAAAAAGCGAAGAAACT-

PANVA_LMG24199{T} GAGCAGGCCTTCTACATGGTTGGTGCCATCGAAGAAGCCGTGGAAAAAGCGAAGAAACT-

PANEU_LMG24197{T} GAGCAGGCCTTCTACATGGTTGGCGCCATCGAAGAAGCCGTGGAAAAAGCGAAGAAACT-

PANAG_DSM3493{T} GAGCAGGCCTTCTACATGGTTGGCGCCATCGAAGAAGCCGTGGAAAAAGCGAAGAAACT-

PANAG_CFBP13505 GAGCAGGCCTTCTACATGGTTGGCGCCATCGAAGAAGCCGTGGAAAAAGCGAAGAAACT-

MIXAL_LTYR-11Z{T} GAACAGGCCTTCTACATGGTTGGTGCCATCGAAGAAGCCGTGGAAAAAGCGAAGAAACT-

MIXTH_QC88-366{T} GAGCAGGCCTTTTACATGGTGGGTTCCATCGAAGAAGCCGTGGAAAAAGCGAAGAAACT-

MIXCA_DSM22759{T} GAGCAGGCCTTCTACATGGTTGGCGCCATCGAAGAAGCCGTGGAAAAAGCGAAGAAACT-

MIXGA_DSM22758{T} GAGCAGGCCTTCTACATGGTTGGCGCCATCGAAGAAGCCGTGGAAAAAGCGAAGAAACT-

PANSE_LMG5345{T} GAGCAGGCTTTCTACATGGTCGGCGCTATCGAAGAAGCCGTGGAAAAAGCGAAGAAACT-

PANEU_LMG5346{T} GAGCAGGCGTTCTACATGGTCGGCGCTATCGAAGAAGCCGTAGAAAAAGCGAAGAAACT-

PANDI_CCUG25232{T} GAGCAGGCGTTCTACATGGTCGGCGCGATCGAAGAAGCTGTCGAAAAAGCGAAGAAACT-

PANWA_LMG26277{T} GAGCAGGCGTTCTACATGGTCGGCGCTATCGAAGAAGCGGTAGAAAAAGCGAAGAAACT-

PANRO_LMG26273{T} GAGCAGGCGTTCTACATGGTCGGCGCTATCGAAGAAGCCGTAGAAAAAGCGAAGAAACT-

PANRW_LMG26275{T} GAGCAGGCGTTCTACATGGTCGGCGCTATCGAAGAAGCCGTAGAAAAAGCGAAGAAACT-

PANCY_LMG2657{T} GAGCAGGCTTTCTACATGGTTGGCGCGATCGACGAAGCTGTGGAAAAAGCGAAGAAACT-

PANST_CCUG26359{T} GAGCAGGCCTTCTACATGGTTGGTGCCATCGAAGAAGCGGTCGAAAAAGCGAAGAAACT-

PANST_LMG2632{PT} GAGCAGGCCTTCTACATGGTTGGTGCCATCGAAGAAGCGGTCGAAAAAGCGAAGAAACT-

PANAL_LMG24248{T} GAGCAGGCCTTCTACATGGTTGGCGCCATCGAAGAAGCGGTCGAAAAAGCGAAGAAACT-

PANAN_LMG2665{T} GAGCAGGCCTTCTACATGGTTGGCGCCATCGAAGAAGCGGTCGAAAAAGCGAAGAAACT-

PANAN_RSA47 GAGCAAGCCTTCTACATGGTTGGCGCCATCGAAGAAGCGGTCGAAAAAGCGAAGAAACT-

TATSA_NML06-3099{T} GAGCAGGCGTTCTACATGGTAGGTACCATCGACGAAGCGGTTGAAAAGGCGAAGAAACT-

TATPT_ATCC33301{T} GAGCAGGCTTTCTACATGGTTGGTTCTATTGACGAAGCGGTAGAAAAAGCTAAGAAACT-

TATCI_DSM13699{T} GAGCAAGCTTTCTACATGGTTGGTTCCATCGACGAAGCTGTAGAAAAAGCGAAGAAACT-

TATMO_LMG23360{T} GAGCAAGCTTTCTACATGGTTGGTTCCATCGACGAAGCTGTAGAAAAAGCGAAGAAACT-

BURGL_LMG2196{T} GAACAGGCGTTCTACATGGTCGGCACGATCGACGAAGCCTTCGAGAAGGCCAAGAAGATC

XANOR_ATCC35933{T} GAGCAGGCGTTCTACATGGTCGGCAGCATCGAAGAAGCCGTCGAGAAAGCCAACAAGATG

XANOR_CFBP2286{PT} GAGCAGGCGTTCTACATGGTCGGCAGCATCGAAGAAGCCGTCGAGAAAGCCAACAAGATG

PSEFU_LMG2158{T} GAACAAGCGTTCTACATGGTTGGCGGCATCGAAGAAGCGATCGAGAAAGCCAAGAAACT-

PSEOR_KCTC32247 GAGCAGGCGTTCTACATGGTCGGCAGCATCGACGAAGCCATCGAGAAGGCCAAGAAACT-

** ** ** ******** ** ** ** **** * * ** ** * ** *

SPHME_DAPP-PG224{T} GCCGCCGAGGCCTAA

SPHPA_NCTC11030{T} GCCCAGGAGGCCTGA

PANAN_LMG2558{T} -----------GTAA

PANBR_LMG5343{T} -----------GTAA

PANCO_LMG24534{T} -----------GTAA

PANDE_LMG24200{T} -----------GTAA

PANVA_LMG24199{T} -----------GTAA

PANEU_LMG24197{T} -----------GTAA

PANAG_DSM3493{T} -----------GTAA

PANAG_CFBP13505 -----------GTAA

MIXAL_LTYR-11Z{T} -----------GTAA

MIXTH_QC88-366{T} -----------GTAA

MIXCA_DSM22759{T} -----------GTAA

MIXGA_DSM22758{T} -----------GTAA

PANSE_LMG5345{T} -----------GTAA

PANEU_LMG5346{T} -----------GTAA

PANDI_CCUG25232{T} -----------GTAA

PANWA_LMG26277{T} -----------GTAA

PANRO_LMG26273{T} -----------GTAA

PANRW_LMG26275{T} -----------GTAA

PANCY_LMG2657{T} -----------GTAA

PANST_CCUG26359{T} -----------GTAA

PANST_LMG2632{PT} -----------GTAA

PANAL_LMG24248{T} -----------GTAA

PANAN_LMG2665{T} -----------GTAA

PANAN_RSA47 -----------GTAA

TATSA_NML06-3099{T} -----------GTAA

TATPT_ATCC33301{T} -----------GTAA

TATCI_DSM13699{T} -----------GTAA

TATMO_LMG23360{T} -----------GTAA

BURGL_LMG2196{T} CA---------GTAA

XANOR_ATCC35933{T} AGCGCCAAGGCGTAA

XANOR_CFBP2286{PT} AGCGCCAAGGCGTAA

PSEFU_LMG2158{T} -----------GTAA

PSEOR_KCTC32247 -----------GTAA

* *
